# Highly Divergent Mitochondrial ATP Synthase Complexes in *Tetrahymena thermophila*

**DOI:** 10.1371/journal.pbio.1000418

**Published:** 2010-07-13

**Authors:** Praveen Balabaskaran Nina, Natalya V. Dudkina, Lesley A. Kane, Jennifer E. van Eyk, Egbert J. Boekema, Michael W. Mather, Akhil B. Vaidya

**Affiliations:** 1 Center for Molecular Parasitology, Department of Microbiology and Immunology, Drexel University College of Medicine, Philadelphia, Pennsylvania, United States of America; 2 Department of Biophysical Chemistry, Groningen Biomolecular Sciences and Biotechnology Institute, University of Groningen, The Netherlands; 3 Department of Medicine, The Johns Hopkins University, Baltimore, Maryland, United States of America; 4 Department of Biological Chemistry, The Johns Hopkins University, Baltimore, Maryland, United States of America; University of California Davis, United States of America

## Abstract

The F-type ATP synthase complex is a rotary nano-motor driven by proton motive force to synthesize ATP. Its F_1_ sector catalyzes ATP synthesis, whereas the F_o_ sector conducts the protons and provides a stator for the rotary action of the complex. Components of both F_1_ and F_o_ sectors are highly conserved across prokaryotes and eukaryotes. Therefore, it was a surprise that genes encoding the a and b subunits as well as other components of the F_o_ sector were undetectable in the sequenced genomes of a variety of apicomplexan parasites. While the parasitic existence of these organisms could explain the apparent incomplete nature of ATP synthase in Apicomplexa, genes for these essential components were absent even in *Tetrahymena thermophila*, a free-living ciliate belonging to a sister clade of Apicomplexa, which demonstrates robust oxidative phosphorylation. This observation raises the possibility that the entire clade of Alveolata may have invented novel means to operate ATP synthase complexes. To assess this remarkable possibility, we have carried out an investigation of the ATP synthase from *T. thermophila*. Blue native polyacrylamide gel electrophoresis (BN-PAGE) revealed the ATP synthase to be present as a large complex. Structural study based on single particle electron microscopy analysis suggested the complex to be a dimer with several unique structures including an unusually large domain on the intermembrane side of the ATP synthase and novel domains flanking the c subunit rings. The two monomers were in a parallel configuration rather than the angled configuration previously observed in other organisms. Proteomic analyses of well-resolved ATP synthase complexes from 2-D BN/BN-PAGE identified orthologs of seven canonical ATP synthase subunits, and at least 13 novel proteins that constitute subunits apparently limited to the ciliate lineage. A mitochondrially encoded protein, Ymf66, with predicted eight transmembrane domains could be a substitute for the subunit a of the F_o_ sector. The absence of genes encoding orthologs of the novel subunits even in apicomplexans suggests that the *Tetrahymena* ATP synthase, despite core similarities, is a unique enzyme exhibiting dramatic differences compared to the conventional complexes found in metazoan, fungal, and plant mitochondria, as well as in prokaryotes. These findings have significant implications for the origins and evolution of a central player in bioenergetics.

## Abstract 2

Tetrahymena ATP synthase, an evolutionarily divergent protein complex, has a very unusual structure and protein composition including a unique Fo subunit a and at least 13 proteins with no orthologs outside of the ciliate lineage.

## Author Summary

Synthesis of ATP, the currency of the cellular energy economy, is carried out by a rotary nano-motor, the ATP synthase complex, which uses proton flow to drive the rotation of protein subunits so as to produce ATP. There are two main components in mitochondrial F-type ATP synthase complexes, each made up of a number of different proteins: F_1_ has the catalytic sites for ATP synthesis, and F_o_ forms channels for proton movement and provides a bearing and stator to contain the rotary action of the motor. The two parts of the complex have to interact with each other, and critical protein subunits of the enzyme are conserved from bacteria to higher eukaryotes. We were surprised that a group of unicellular organisms called alveolates (including ciliates, apicomplexa, and dinoflagellates) seemed to lack two critical proteins of the F_o_ component. We have isolated intact ATP synthase complexes from the ciliate *Tetrahymena thermophila* and examined their structure by electron microscopy and their protein composition by mass spectrometry. We found that the ATP synthase complex of this organism is quite different, both in its overall structure and in many of the associated protein subunits, from the ATP synthase in other organisms. At least 13 novel proteins are present within this complex that have no orthologs in any organism outside of the ciliates. Our results suggest significant divergence of a critical bioenergetic player within the alveolate group.

## Introduction

Mitochondrial F-type ATP synthase complexes are remarkable molecular machines that link proton-motive force generated by respiration to the synthesis of ATP, the currency of energy economy in biology. The eukaryotic enzyme is made up of two structural sectors, the F_o_ and the F_1_ (hence, the complex is often called the F_o_F_1_ or F_1_F_o_ complex; complex V is another common designation, referring to the fifth and final complex of the oxidative phosphorylation pathway). The membranous F_o_ sector consists of a subunit c oligomer, subunit a, the peripheral stalk subunits b, d, F_6_ (h), and OSCP, as well as additional associated subunits depending on the species. The globular catalytic sector F_1_ is made up of subunits α_3_, β_3_, and the central stalk subunits γ, δ, and ε [1],[2]. The movement of protons through a channel constituted by the a and c subunits provides the energy required for the clockwise rotation of the c ring, which in turn causes the central stalk to rotate because of its close contact with the c ring. The rotation of the central stalk subunit γ creates a conformational change in the catalytic subunits β and α, which are in contact with the upper portion of γ, leading to the synthesis of ATP from bound ADP and phosphate [1],[3]–[6]. When the central stalk rotates, it is critical that α_3_β_3_ subcomplex is held in position, and this is accomplished by the peripheral stalk that acts as a bearing and a stator [7],[8]. The origin of proton-driven ATP synthesis by the F_o_F_1_ complex can be traced to the Eubacteria. Because of the critical nature of interactions between the F_o_ and F_1_ sectors that underlie the functioning of this complex [2], the subunit proteins that form the essential core of the complex are highly conserved, and the genes encoding them are usually readily identified in complete genomic sequences of prokaryotes and eukaryotes.

When we searched the genome sequences of apicomplexan parasites [9]–[12], we were intrigued by the apparent absence of genes encoding the F_o_ sector subunits that form the peripheral stalk (except OSCP) as well as the subunit a of ATP synthase, although F_1_ sector subunits and the F_o_ subunit c were readily detected. Clearly, a functional ATP synthase complex cannot be assembled without these subunits. We initially reasoned that the parasitic existence of these organisms might underlie the loss of a functional ATP synthase, possibly through a greater reliance on hosts for energy generation. However, publication of the macronuclear genome sequence of the ciliate *T. thermophila* [13] revealed that the same set of proteins apparently missing in the apicomplexans was also undetectable in this ciliate. The ciliates and the apicomplexans (along with dinoflagellates) belong to a “crown group” of thousands of organisms called alveolates that is phylogenetically distant from metazoans, fungi, and plants [14]–[16]. It is possible that during evolution, these subunits may have diverged in these lineages beyond the point of identification using current bioinformatics tools, although such subunits are readily detectable by the same tools in evolutionarily more distant prokaryotic genomes. Alternatively, novel proteins may have been recruited to fulfill functions of the missing subunits. It is also possible that the retained ATP synthase subunits (i.e., those forming the F_1_ sector) may serve functions other than ATP synthesis in these organisms. However, several studies done in the 1970s showed *Tetrahymena* mitochondria to be capable of oxidative phosphorylation [17]–[19]. Therefore, it seemed more likely that novel or highly divergent subunits may have replaced the conventional a and peripheral stalk subunits in *T. thermophila*, leading to a unique but fully functional enzyme. Further, such novel subunits might be shared by the members of the whole clade of alveolates, if they were adopted by an early common ancestor of the ciliates, dinoflagellates, and apicomplexans.

The ease with which *Tetrahymena* can be grown, the size of the cells, the abundance of mitochondria in each cell, and availability of standardized techniques to isolate mitochondria made *Tetrahymena* an attractive model to study the ATP synthase of alveolates. Although *Tetrahymena* has served as a model eukaryote and has been the subject of many seminal studies that have resulted in numerous important insights in biology [20]–[22], its ATP synthase has not been investigated. We show in this report that *Tetrahymena*'s ATP synthase possesses an unusual structure, with similarity in the F_1_ headpiece morphology, but significant differences are seen in its dimer shape and in protein mass on the intermembrane side of the complex compared to previously studied ATP synthases from a variety of other organisms. In addition to readily identifiable F_1_ subunits, the enzyme appears to contain several subunits that have no known orthologs in other organisms. The absence of orthologs to these novel subunits even in apicomplexans and dinoflagellates suggests that the ciliate ATP synthase is truly unique.

## Results

### Oxidative Phosphorylation in *T. thermophila* Strain SB210

**Figure 1:**
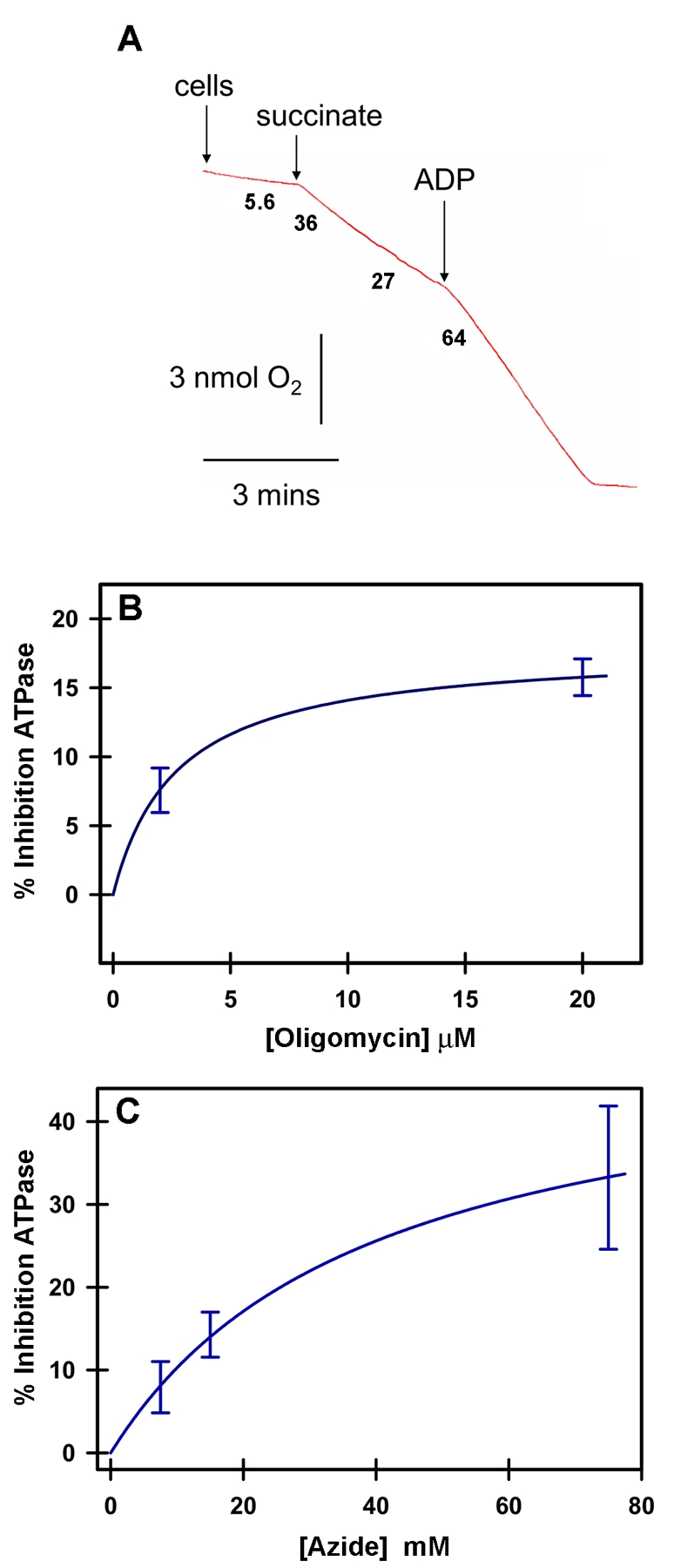
ADP-stimulated oxygen consumption and inhibitor insensitivity of ATPase activity in *T. thermophila* mitochondria. (A) Succinate-dependent oxygen consumption in digitonin-permeabilized *T. thermophila* cells is stimulated upon addition of ADP, consistent with oxidative phosphorylation by a mitochondrial electron transport chain and ATP synthase. Additions to the oxygen electrode reaction chamber are indicated by arrows. Final concentrations were 0.3 µg/µl cell protein, 5 mM succinate, 17 µM ADP. Numbers below the trace give the rate of oxygen consumption in nmoles O_2_/min/mg protein). (B) and (C) show the concentration dependence of the inhibition of the ATPase activity of isolated *T. thermophila* mitochondria by oligomycin and sodium azide, respectively. Error bars indicate standard deviation (*n* = 3). Yeast mitochondrial ATPase activity was inhibited 50% by 1 µM oligomycin and 91% by 1 mM sodium azide (not shown) under these assay conditions.

Previous studies on oxidative phosphorylation in *Tetrahymena* were carried out in the 1970s using strains that were not always defined. Since our goal was to take advantage of the sequenced *T. thermophila* genome to identify ATP synthase subunits, for all our studies we decided to use the same strain (SB210) for which the macronuclear genomic sequence has been published [13]. To confirm that mitochondria from this strain were comparable to those used in previous studies, we assessed the in situ capability of the mitochondria in digitonin-permeabilized *T. thermophila* cells to carry out oxidative phosphorylation, which is indicative of a functional ATP synthase and electron transport chain, in respirometry experiments. A typical oxygen consumption trace is shown in Figure 1A, in which respiration was dependent on the presence of mitochondrial substrate, succinate, and stimulated 2.4-fold by the addition of ADP. Similar results were obtained in earlier studies of mitochondria from various isolates of *T. pyriformis* [23]. Stimulation of the rate of respiration in this type of experiment is due to increased utilization of the proton gradient by the ATP synthase to drive the synthesis of ATP from the added ADP; the rate of respiration increases in response to the reduction of the proton gradient.

Under appropriate conditions, F_o_F_1_ ATP synthases are capable of the reverse reaction, i.e., ATP hydrolysis. Indeed, in a number of organisms the reverse reaction is important for maintenance of the proton electrochemical gradient under specific growth conditions or life stages [24]–[27]. A coupled spectrophotometric assay (see Materials and Methods) was used to assess the ATP hydrolase activity in *T. thermophila* mitochondrial preparations. Reaction traces (Figure S1) show that *T. thermophila* SB210 ATP hydrolase exhibits time- and ATP-dependent activation, as seen in other F_o_F_1_ ATP synthases/hydrolases [28]. *T. thermophila* mitochondria had a somewhat lower specific ATPase activity compared to yeast (unpublished data); however, it is possible that the measured activity represents only a fraction of the ATP synthase complexes present in the mitochondrial membranes, since isolated dimeric complexes exhibited negligible hydrolase activity (see below). The measurable ATPase activity also showed unusual resistance to the classical F_o_F_1_ ATP synthase inhibitors oligomycin and sodium azide (Figure 1B and 1C). Similar resistance to oligomycin, as well as other inhibitors, was previously reported in mitochondria from *T. pyriformis* [23],[29].

**Figure 2:**
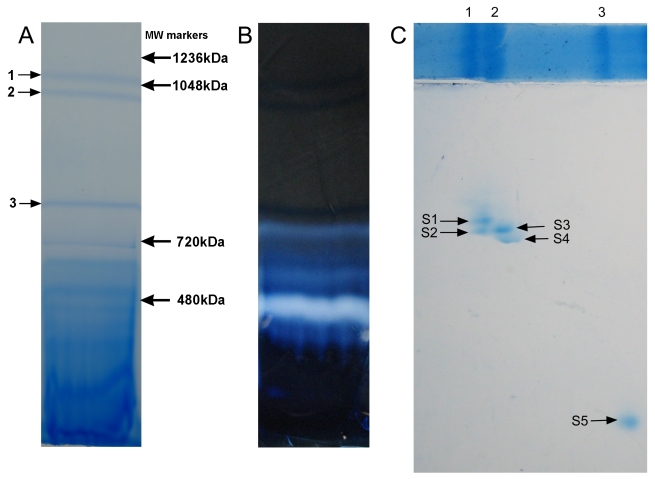
BN-PAGE of solubilized *T. thermophila* mitochondrial membranes. (A) BN gel (3%–10%) run with *T. thermophila* mitochondria (1 mg) solubilized with digitonin (5 µg of detergent/µg of protein), and stained with colloidal Coomassie blue. (B) In-gel ATPase staining of a BN gel strip of digitonin-solubilized *T. thermophila* mitochondria. The gel was incubated overnight (8–12 h) and was briefly (2 min) washed with 10% acetic acid to remove excess lead precipitate on the surface. (C) 2-D BN/BN-PAGE. The first dimension was completed as in (A) and a strip was excised and briefly soaked in cathode buffer containing 0.03% docecyl maltoside (the strip shown here above the 2-D gel is a second strip cut from the same 1-D BN-PAGE that was stained with Coomassie blue; the image of the strip was cropped below the position of band 3). The second dimension was a 4%–12% gradient BN-PAGE run with 0.03% dodecyl maltoside in the cathode buffer (see Materials and Methods). The band 1 (V_2_, I+III_2_, II_2_) separated into two spots designated as spot 1 and 2. The band 2 (V_2_, I+III_2_) separated into two spots designated as spot 3 and 4, while band 3 (III_2_) ran as a single spot, labeled as spot 5. The image of the 2-D gel was cropped on the right side so that most of the material running below band 3/spot 5 is not shown.

ATP synthase generally forms the second largest complex after complex I and runs as high molecular weight bands in blue native (BN) polyacrylamide gel electrophoresis (PAGE) [30]. *T. thermophila* mitochondria were solubilized with digitonin or dodecyl maltoside and separated on a 3%–10% gradient BN gel to resolve high molecular weightweight complexes (Figure 2A). We assessed the ATP hydrolase activity of the sample bands using an in-gel ATPase assay that generates a white precipitate. In digitonin-solubilized fractions, the principal regions of ATPase activity were found lower down in the gel (below band 3), and thus may be due to monomers and/or separate catalytic F_1_ head pieces (Figure 2B). Even after overnight incubation (8–12 h), we saw only a very limited amount of precipitate in the top two bands, which contain dimeric ATP synthase complexes on the basis of single particle electron microscopy results (see below), indicating very weak ATPase activity, in contrast to active dimeric and higher oligomeric forms of ATP synthase complexes previously reported in other species [30]–[33].

### Novel Structural Features of *Tetrahymena* ATP Synthase

**Figure 3:**
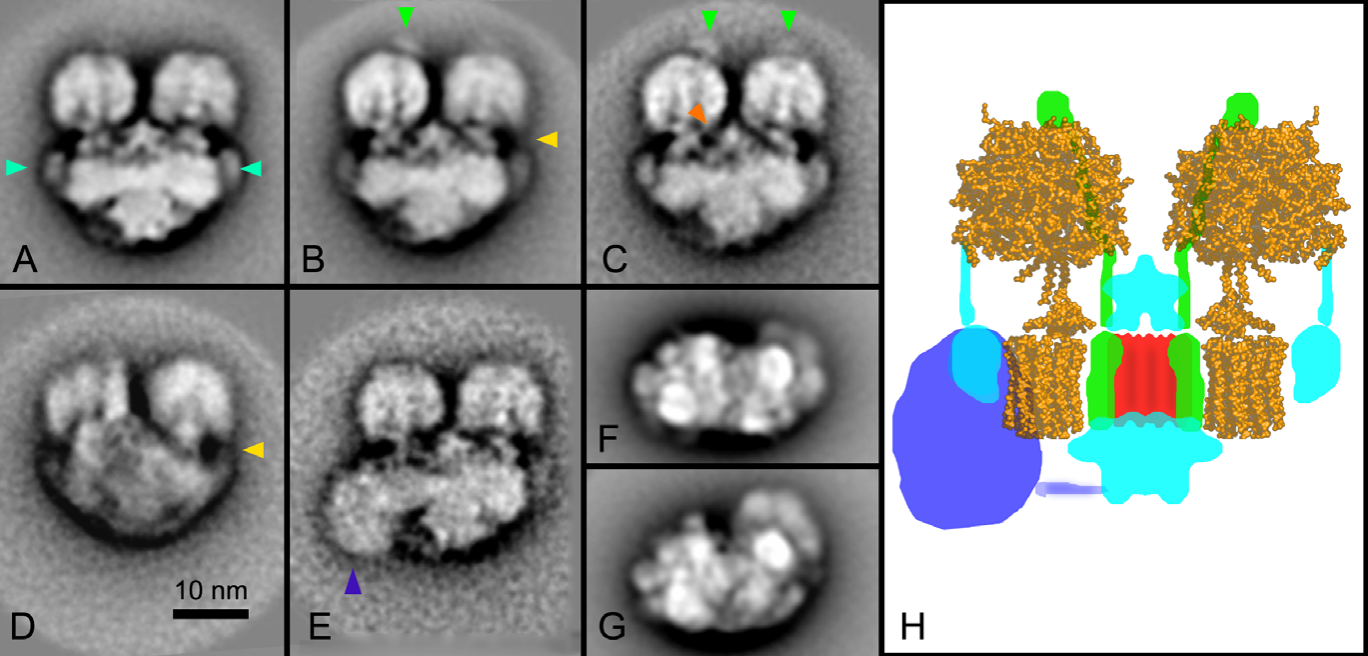
2-D projection maps of dimeric ATP synthase from *Tetrahymena thermophila*. (A–E) represent the side view, (F) top view, and (G) intermediate view. The Complexes were extracted either with digitonin (F, G), dodecyl maltoside (A, D, E), or the mixed dataset was used for image analysis (B, C). (E) Dimeric ATP synthase with an additional density next to the c subunit rotor of the left monomer (sum of 64 projections). (H) Interpretation of the projection (B, average of 3,254 projections) with the help of X-ray structure of yeast ATP synthase (PDB accession number 1QO1, [34]). Blue arrowheads (A) mark additional subunits on the extreme left and right positions of the c subunit rotors; green arrowheads (B and C), OSCP subunits; yellow arrowheads (B and D) point to an apparent connection between the domains seen at the extremities of the c rotor (cf., blue arrowheads in (A) and the F_1_ headpiece); the orange arrowhead (C), to a connection between the F_1_ part and the matrix exposed domain; and the dark blue arrowhead (E) points to an unknown large extra mass attached to F_o_. The bar represents 10 nm and applies to all frames.

The complexes from the highest molecular weight bands (Figure 2A, bands 1, 2, and 3) were electroeluted under gentle conditions that largely preserved their structure and analyzed by single particle electron microscopy. In the samples from bands 1 and 2, we observed structures resembling dimeric ATP synthase complexes (complex V_2_), as well as apparent supercomplexes of complex I (NADH dehydrogenase), and a dimer of complex III (complex I–III_2_). Band 3 appeared to contain complex III dimers (Figure S2). Since the electroeluted particles from these bands were quite uniform, without breakdown products, we were able to select homogenous datasets of 40,000 single particle images obtained after digitonin or dodecyl maltoside solubilization and used them to generate averaged 2-D projection maps. Analysis of the projections indicated that subsets of projections from digitonin and dodecyl maltoside comprised the same types of projections. Hence, we combined the data to improve the quality of final images. Side-view projection maps of *T. thermophila* dimeric ATP synthase showed particles attached in a parallel and flat position on the carbon support film (Figure 3A–3C) or in a slightly tilted position (Figure 3D). Some dimers appeared to have a large protein attached to the F_o_ sector (Figure 3E, blue arrowhead). As estimated from its surface area, the mass of this domain could be as much as 200 kDa. In addition, we also obtained top views of the dimers (Figure 3F and 3G). The best maps had a resolution of about 1.5 nm, which permitted recognition of specific known and novel features as depicted in a schematic model (Figure 3H). The projection maps indicated that the all structural elements of mammalian and *Escherichia coli* enzyme were present, including the F_1_ headpiece consisting of α_3_β_3_ subunits [34], the rotor composed of the subunit c ring, as well as the central stalk (rotor) consisting of the γ, δ, and ε subunits. In addition, OSCP, the uppermost stator component (Figure 3B and 3C, see green arrowheads) was present, although it was apparently lost from a substantial number of projections (Figure 3A). The headpieces are separated by at least 2 nm, and there is protein density present in the dimer interface region between the two F_o_ parts (marked red in H) that appears structurally similar to that previously observed in the alga and yeast ATP synthases [35],[36]. All other visible densities (marked blue) appear to be unique to *T. thermophila* dimeric ATP synthase since they have not been observed in any other species. There were two domains attached at the interface of the monomers. A large domain, estimated to be at least 100 kDa, was attached to the bottom side of the complex; another was at the matrix-exposed side close to the F_1_ head pieces and seemed to be connected to the catalytic F_1_ part (Figure 3C, orange arrowhead). The latter density could represent novel subunits that help the two monomers associate with each other. Interestingly, the dimer also had distinct novel membrane-bound densities at the extreme left and right position of the c subunit rotors (Figure 3A, blue arrowheads). Some dimers appeared to have a large protein attached to the F_o_ sector (Figure 3E, dark-blue arrowhead). As estimated from its surface area, the mass of this domain could be as much as 200 kDa. Furthermore, the two monomers appeared to be parallel to each other, rather than forming an acute angle as seen in the other species examined thus far. This finding was dramatically different from projections of the yeast, *Polytomella*, and bovine complexes [35]–[39].

A final question is the position of the two stators of the dimer. This question is difficult to answer because the stators are strongly overlapping with the F_1_ headpieces. One possibility is that they are at the extreme periphery. In some views there is a faint connection between the headpiece and the membrane (Figure 3B, yellow arrowhead). This connection becomes stronger upon tilting (Figure 3D). On the other side, there is ample space in the center of the dimer where an extensive structure resides in between the F_1_ headpieces. This structure is connected to the headpiece (Figure 3C, orange arrowhead) and may hide the stator. The latter position may be considered more likely by reason of structural homology. The yeast, *Polytomella*, and bovine ATP synthase complexes have one stator per monomer [35],[37]–[39], but fully lack the peripheral domains marked light blue (Figure 3C).

### Additional Supercomplexes in Bands 1–3

In addition to dimeric ATP synthase, bands 1 and 2 contained complex I–III_2_ supercomplex and projection maps of its side and top view were analyzed (Figure S2). These maps resemble their counterparts in *Arabidopsis* and other organisms [37],[40],[41]. Complex III_2_ was located at the tip of the membrane arm of complex I (Figure S2A, white arrowhead). A small number of complexes lacked a part of the hydrophilic arm (Figure S2C, black arrowhead), which has been observed in many complex I preparations. The assignment of the position of complex III_2_ in the supercomplex was confirmed by a structural analysis of single dimeric complex III, eluted from band 3 (Figure S2D). Features of the matrix-exposed domain, which are part of the subunits 1 and 2 of complex III, were similar in both types of particles (Figure S2A, D white arrowheads). Complex III_2_ from *T. thermophila* was structurally comparable to its counterpart in *Arabidopsis* (Figure S2E), but not identical [40]. Overall, the I–III_2_ supercomplex and the dimer of complex III were structurally similar to those of many other organisms, suggesting conservation of these respiratory complexes in *T. thermophila*.

### Conserved and Novel Subunits in ATP Synthase

The three high molecular weight bands identified by BN-PAGE were excised from gels for analysis by liquid chromatography-mass spectrometry-mass spectrometry (also known as liquid chromatography-tandem mass spectrometry or LC/MS/MS). Samples excised from gel runs were divided and separately digested with trypsin or chymotrypsin to improve the chances of detecting hydrophobic proteins. The digests were subjected to LC/MS/MS analyses as described in the Materials and Methods. Overall, peptides originating from 59 proteins were identified in band 1. The main annotated proteins in band 1 were subunits of respiratory complexes I, II, III, and V, as well as some additional proteins. In band 2 we detected peptides originating from 50 proteins, including subunits of complexes I, III, and V. In band 3, there were 21 protein hits, including subunits of complex III. There were also many unannotated proteins and some apparent contaminating proteins (i.e., proteins that are not known to be part of oxidative phosphorylation complexes) in each of the three bands. Data for all these peptides are summarized in Table S1; LC/MS/MS data for peptides detected in bands 1–3 are given in Table S2.

The presence of multiple complexes in the BN-PAGE bands made it difficult to assign any of the observed hypothetical proteins to specific complexes. To achieve further separation of the complexes, we carried out 2-D BN/BN-PAGE. The presence of 0.03% dodecyl maltoside in the cathode buffer of the second dimension BN-PAGE was strong enough to dissociate Band 1 and Band 2 of the first dimension BN-PAGE into two individual spots (Figure 2C, designated spot 1 and spot 2 from band 1, and spots 3 and 4 from band 2). Band 3 ran as a single spot, which was designated as spot 5 (Figure 2C). The dissociation pattern observed in 2-D BN/BN-PAGE was reproducible. Samples were excised from the central portion of each spot for analysis. A set of spots from one 2-D gel was digested with trypsin and a set from a second was digested with chymotrypsin for LC/MS/MS analysis. The results revealed that Spot 1, and to a lesser extent, spot 3 contained conventional ATP synthase subunits including α, β, γ, OSCP, and c (ATP9), whereas spots 2, 4, and 5 largely did not, but rather contained subunits normally found in complexes I and III, as well as other proteins. A summary of data from all five spots is given in Table S1, and the LC/MS/MS data for peptides detected in spots 1–5 are given in Table S3. In addition to the annotated ATP synthase subunits, spot 1 LC/MS/MS results included three additional proteins normally not associated with ATP synthases (branched-chain amino acid aminotransferase family protein, lipid A-disaccharide synthase, and peptidase M16 inactive domain-containing protein; however the latter two may be contaminants as described below), and 15 hypothetical or uncharacterized proteins that have no obvious homology to any other proteins in the database (Table 1). As noted in a previous study of the *T. thermophila* mitochondrial proteome by Smith et al. [42], many of the original gene models merit some corrections. In our analysis, we utilized appropriately corrected sequences for several of the proteins on the basis of data from Smith et al., extant EST data, or comparison with data from the related ciliate, *Paramecium tetraurelia*. Among the corrected uncharacterized proteins we detected low, but significant, similarity to two additional ATP synthase subunits—118355322 to F_1_ subunit δ and 118360532 to F_o_ subunit d (Table 2; evidence for these assignments is provided in Texts S1 and S2). On the basis of the prevalence of peptides from known ATP synthase subunits in spot 1 data (see Table 1, “Unique Peptides” column), their near absence from spots 2, 4, and 5, and the near absence in spot 1 of peptides from known subunits of other mitochondrial complexes (Tables S1 and S3), we considered it likely that the remaining uncharacterized proteins in Table 2 were authentic subunits of the complex V dimer. On the other hand, the peptides from two of the annotated proteins, peptidase M16 inactive domain-containing protein and lipid A-disaccharide synthase, were predominantly found in spot 5 and in spots 3 and 4, respectively (Tables 1 and S3). These proteins may thus represent contaminants present in spot 1 because of trailing in the gels.

**Table 1:** Assigned proteins detected in BN-PAGE Spot 1 by LC/MS/MS.

NCBI ID^a^	Description^b^	Unique Peptides^c^	Size^d^	TMH^e^
15027647	ATP synthase F_o_ subunit 9 (subunit c)	3/3	76	2,2,2
146182760	ATP synthase F_1_ delta subunit (OSCP)	3/3	219	0,0,0
118355000	ATP synthase F_1_ gamma subunit	4/4	299	0,0,0
146184059	ATP synthase F_1_ alpha subunit	17/19	546	0,0,0
146185860	ATP synthase F_1_ beta subunit	18/19	497	0,0,0
118384478	Branched-chain amino acid aminotransferase family protein	2/2	406	0,1,0
118397639	Lipid A-disaccharide synthase	2/14	516	0,0,0
118384179	Peptidase M16 inactive domain containing protein	3/27	482	0,0,0

a gi number of the deduced protein sequence in the NCBI databases.

b As maintained by NCBI (October 2009).

c Number of unique peptides from each protein detected in spot 1/total number detected in all five spots excised from 2-D BN-PAGE gels.

d Predicted length of each protein in amino acids.

e Number of transmembrane helices in each protein predicted by TMHMM v. 2.0, TOPCONS, and TMMOD servers, respectively.

**Table 2:** Unassigned proteins detected in BN-PAGE spot 1 by LC/MS/MS.

NCBI ID^a^	Description^b^/Proposed Description	Unique Peptides^c^	Size^d^	TMH^e^
118355322^f^	Hypothetical protein/putative F_1_ delta subunit	3/3	158^f^	0,0,0
118360532^f^	Hypothetical protein/putative F_o_ d subunit	3/3	234^f^	0,0,0
15027631	Ymf66/putative ATP synthase subunit a-like protein (see Results)	2/2	446	8,8,8
146175330^f^	Conserved hypothetical protein^g^	8/8	480^f^	0,0,0
146163301	Conserved hypothetical protein^h^	4/4	388	0,0,0
146185889	Hypothetical protein^i^	3/3	209	1,1,1
229594811	Hypothetical protein	2/2	232	0,0,0
146161614^f^	Hypothetical protein	7/7	149^f^	0,1,1
118399953^f^	Hypothetical protein	7/7	247^f^	0,0,0
118366175^f^	Hypothetical protein^i^	7/7	381^f^	1,2,2
118370910	Hypothetical protein	3/3	120	0,0,0
229594147	Hypothetical protein	9/10	273	0,0,0
146180703^f^	Hypothetical protein	2/2	221^f^	0,1,0
118398278	Hypothetical protein^i^	3/3	179	1,1,0
118398135^f^	Hypothetical protein	3/3	152^f^	0,1,0
146184052	Hypothetical protein	2/2	204	0,1,0

a gi number of the deduced protein sequence in the NCBI databases.

b As maintained by NCBI (October 2009).

c Number of unique peptides from each protein detected in spot 1/total number detected in all five spots excised from 2-D BN-PAGE gels.

d Predicted length of each protein in amino acids.

e Number of transmembrane helices in each protein predicted by TMHMM v. 2.0, TOPCONS, and TMMOD servers, respectively.

f Gene model and protein sequence revised, see Table S4.

g Similarity reported in NCBI Conserved Domains to COG1252, Ndh, NADH dehydrogenase, FAD-containing subunit (E = 4e–10) and COG0446, HcaD, uncharacterized NAD(FAD)-dependent dehydrogenases (E = 6e–9), both members of Superfamily cl11411: pyridine nucleotide-disulphide oxidoreductase.

h Similarity reported in NCBI Conserved Domains to COG0596, MhpC, predicted hydrolases or acyltransferases (alpha/beta hydrolase superfamily) (general function prediction only) (E = 2e–07) and pfam00561, abhydrolase_1, alpha/beta hydrolase fold (E = 1e–04), members of superfamily cl09107: esterase_lipase.

i See text in Results for description of subunit b-like characteristics of this protein.

### Subunits of the F_o_ Sector

**Figure 4:**
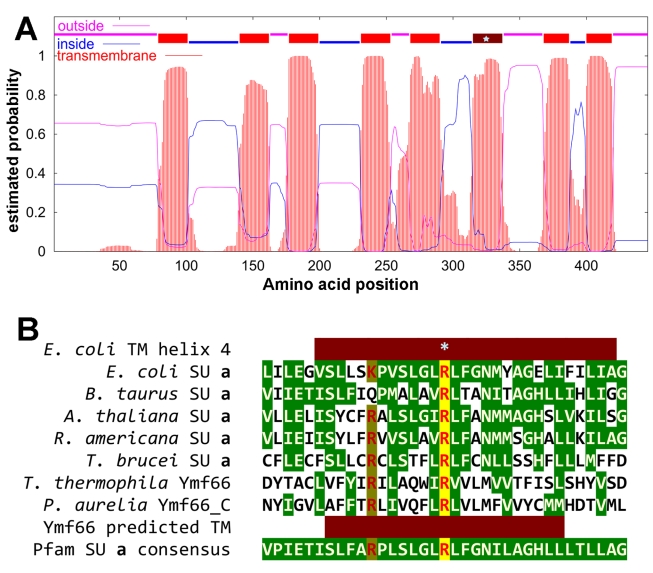
Position of arginines in putative TM6 of Ymf66 and possible alignment with the region of ATP synthase subunits a that contains the conserved Arg. (A) Prediction of transmembrane helices and topology by TMHMM (similar results are obtained with other algorithms). The predicted extent of transmembrane segments is indicated by red bars above the plotted probability scores; the sixth transmembrane segment containing two Arg residues is colored dark red with a star at the position of the second Arg. (B) An alignment of Ymf66 transmembrane helix 6 with the region of ATP synthase subunits a that contains an essential conserved Arg. Residues that are identical to or chemically similar to the consensus amino acid are shown with reverse green coloration; the conserved Arg is shown in red and highlighted with yellow background, and the second partially conserved Arg is shown in dark red with yellow-green background. The extent of known (*E. coli*) and predicted (Ymf66) transmembrane helices is indicated by dark red bars. Sequence data: *E. coli*, gi:16131606; *B. taurus*, gi:60101830; *A. thaliana*, gi:6851018, *R. americana*, gi:2258385; *T. brucei*, gi:343544; *T. thermophila*, gi:15027631; *P. Aurelia*, gi:8928578.

While putative F_o_ subunits c, d, and OSCP were detected, subunits with sequence similarity to the structurally and mechanistically critical integral membrane subunits a and b were not found. However, there were proteins among the LC/MS/MS results with appropriately positioned predicted transmembrane segments that could be evaluated as possible highly divergent subunits or novel functional replacements for these subunits. Ymf66 is an integral membrane protein with approximately eight predicted transmembrane helices. Interestingly, the corresponding region in *Paramecium* mitochondrial DNA (mtDNA) is split into two open reading frames (ORFs) [43]. Ymf66 has several features that are characteristic of F_o_ subunit a in a general fashion: (1) it is encoded by mtDNA (all known subunits a, with one exception, are mitochondrially encoded); (2) it is a multispan membrane protein (subunit a is the only F_o_ subunit with >>2 transmembrane helices); (3) it has a conserved arginine residue embedded in a predicted transmembrane helix in the C-terminal region of the protein (subunit a has a conserved and functionally essential arginine, located in the fourth transmembrane helix in the well-studied *E. coli* subunit (Figures 4 and S2); and (4) the same transmembrane helix also contains another arginine residue that is conserved at a similar position in most ATP synthase a subunits (except vertebrates where it is usually replaced by a glutamine). An examination of Ymf66 from five *Tetrahymena* species, as well as from *Paramecium*, revealed that all these features including appropriately placed arginines were absolutely conserved, providing additional support to our proposition that this protein substitutes for the ATP synthase a subunit in ciliates.

We found five proteins (146185889, 118398278, 118366175, 146161614, 146180703) that could be considered candidates to functionally replace subunit b, on the basis that they contain one to two hydrophobic regions in the N-terminal half of the protein followed by a more hydrophilic C terminus, and fall roughly within the size range of this subunit (predicted topologies of these proteins are given in Figure S4). Secondary structure predictions were consistent with this possibility in the case of the first three proteins (146185889, 118398278, and 118366175), which were predicted to have a predominance of alpha helical structure throughout the region C-terminal to the hydrophobic section (Figure S4). The known and predicted structure of this section of the bovine subunit b is almost entirely composed of an extended α-helix, allowing the matrix section of the subunit to reach from the membrane to near the top of the F_1_ subcomplex [8]. One or more of these candidate proteins could participate in forming the stator or be associated with one of the apparently novel membrane-associated domains observed in the *Tetrahymena* structure.

### Evolutionary Relationships of ATP Synthase Subunits

**Figure 5:**
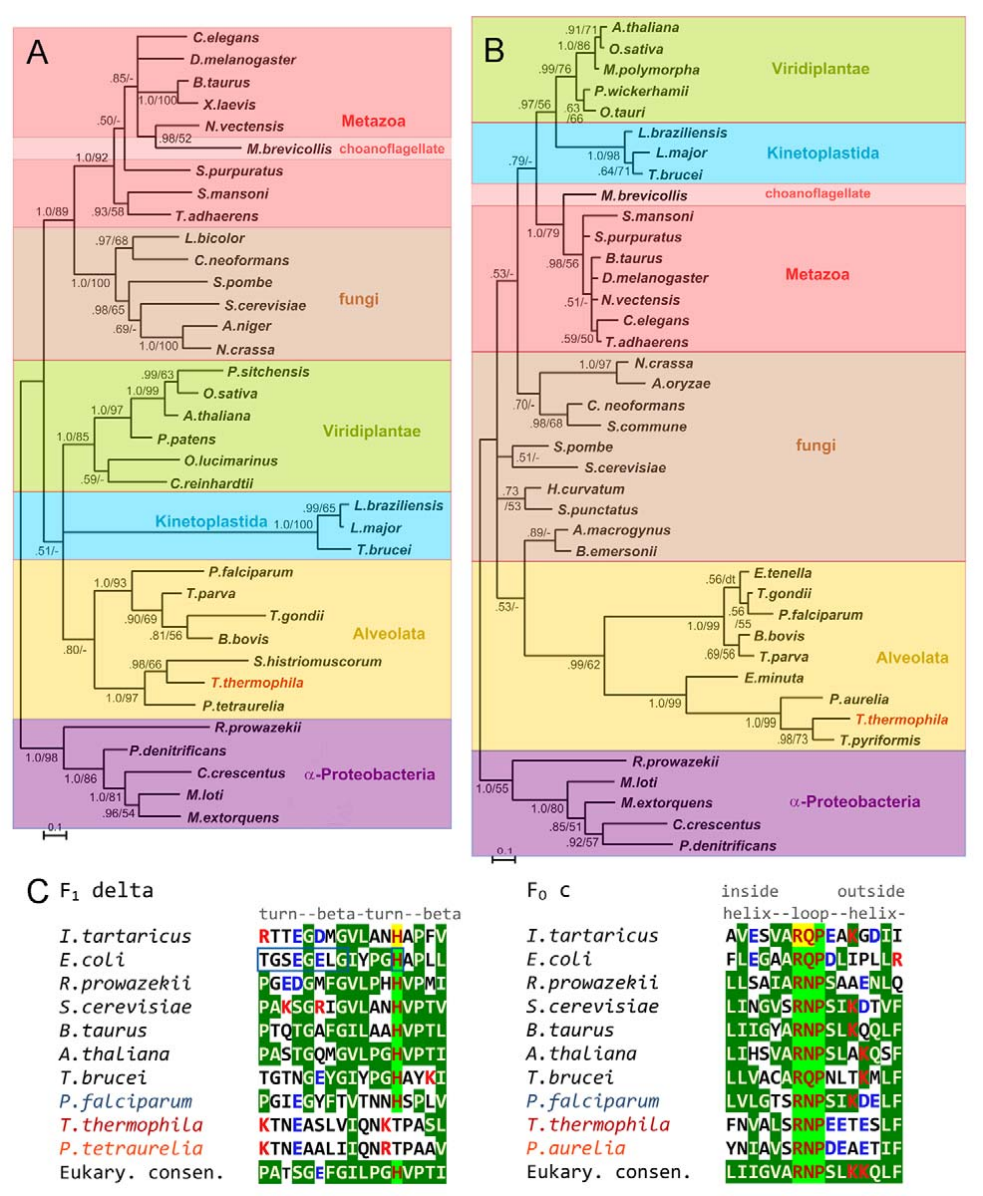
Phylogenetic trees inferred for subunits γ and c and alignment of the interface regions of subunits δ and c. (A and B) show trees inferred by Bayesian analysis (MrBayes [76], see Methods) for subunit γ and subunit c, respectively. Numbers near branch nodes indicate Bayesian posterior probabilities/maximum likelihood bootstrap support (200 replicates) (maximum likelihood analysis employed PhyML [80] [see Methods] indicates a maximum likelihood support of less than 50%; dt indicates a different branch topology was supported by the maximum likelihood analysis). Branches with less than 0.5 posterior probability have been collapsed to a common node. Sequences from α-proteobacterial spp. were included to provide a root, but turned out not to compose the most divergent clade in each analysis; the trees are nevertheless shown as rooted by the bacterial clade. Major taxonomic groups are indicated by color shading (it should be kept in mind, however, that gene product trees can differ from species trees via a number of biological mechanisms, as well as methodological and statistical error). The bar at the lower left provides the scale of substitutions per site. (C) compares the interface regions of subunits δ (denoted ε in prokaryotes) and c from selected prokaryotic and eukaryotic spp. Amino acid residues determined to be critical for interaction by site directed mutagenesis [49] are shown in dark red with a yellow background. Highly conserved residues are dark red on a light green background; those that are identical to or chemically similar to the consensus amino acid are shown with reverse coloration on a green background. (Consensus residues were calculated in Jalview 2.4 [96] using alignments of representative eukaryotic spp. from a broad range of taxa, but omitting ciliates. Consensus is not indicated for two positions of subunit δ that have a very low degree of conservation). Residues of subunit ε that were shown to be in proximity to the loop region of subunit c by cross-linking in the *E. coli* complex [48],[97] are enclosed in boxes. Basic (positive) residues are colored red, and acidic (negative) residues blue. Positions of secondary structural elements as found in high resolution structures of bacterial subunits [98],[99] are indicated above the alignment. Ciliate species names are shown with red-shaded lettering and *P. falciparum*, another alveolate, with blue lettering.

The structural and proteomic analyses seem to suggest unique evolutionary history for many subunits of *Tetrahymena* ATP synthase. Whereas some of the subunits were clearly recognizable as orthologs of ATP synthase subunits from other organisms, there were many others that seem to be limited to ciliates. To understand evolutionary provenance and relationships of the recognizable subunits, we carried out phylogenetic analyses of these subunits. Alignments of the *Tetrahymena* ATP synthase subunits β, γ, δ, and c with orthologs from a broad range of other species were constructed and used to calculate their apparent phylogenetic relationships (Figures 5 and S5; Text S1). The sequences of the catalytic β subunits are well-conserved among all species, with numerous sequence positions that exhibit total amino acid identity. The F_1_ rotor subunit γ is somewhat less conserved but still has a high degree of similarity among species. The phylogenetic reconstructions that included ciliate and apicomplexan β subunit (Figure S5) or γ subunit (Figure 5A) exhibit a similar relationship among the major groups (metazoa, fungi, Viridiplantae, alveolates) as that seen in many phylogenetic studies [44]–[46]. The relatively moderate branch lengths of the ciliate clade (Figure 5A) suggest a rate of genetic change similar to the average of other groups. The kinetoplastids, in contrast, exhibit very long branch lengths, suggesting they have experienced a period of rapid divergence. Proteomic analysis of *Trypanosoma brucei* ATP synthase has recently revealed divergence of this complex as well [47]. When we examined the less well-conserved F_1_ δ and F_o_ c subunits, we found a different pattern. The alveolates, especially the ciliates, exhibit very long branches indicating an accelerated rate of change (Figure 5B). For these smaller and more divergent subunits, the overall phylogeny of the major groups is less well reproduced, probably owing to their greater divergence and shorter length, i.e., there are a relatively small number of positions that can be reliably aligned, and in addition, a degree of saturation at some sites cannot be ruled out. Thus, the well-conserved subunits (α, β, γ) form a contiguous subset of the complex apparently undergoing modest evolutionary change in ciliates, while the δ and c subunits and the evidently even more divergent a-like subunit (Ymf66) may represent a subset coevolving at a much more rapid rate. We assessed one possible specific instance of coevolution by comparing sequences from the interface regions of the δ and c subunits. This interface has been characterized in bacterial ATP synthase and, along with the γ-c and δ-γ interactions, is critical for the transfer of the rotational movement of the c subunit ring to the central stalk [48],[49]. In a functionally essential protein–protein interface region, changes in one partner that affect the interface would normally be matched by compensating changes in the interacting partner that act to maintain function [50],[51]. The divergence of the interface regions of the δ and c subunits (Figure 5C) can be construed as a result of coevolution that probably required a series of compensatory changes. One evident hypothesis, which could potentially be tested experimentally, is the interaction of acidic residues acquired in ciliate subunits c adjacent to the conserved loop residues (RNP) with the basic residue acquired in ciliate subunits δ next to the position of the otherwise conserved histidine (Figure 5C).

## Discussion

### Unique Structural Features of *Tetrahymena* ATP Synthase

There are three types of multiprotein complexes that link ion movement across the membrane with rotational catalysis of ATP synthesis/hydrolysis: the archaeal A-type and bacterial/mitochondrial F-type ATP synthases use H^+^ (or sometimes Na^+^) ions to drive ATP synthesis, whereas the V-type ATPases hydrolyze ATP to pump H^+^ against its concentration gradient. Shared structural features of these molecular machines, such as distinct sectors that constitute the catalytic, ion transport, and stator functions, suggest a common evolutionary ancestry possibly dating back to the origin of cellular life. In general, the individual A-, F-, or V-type ATP synthases/hydrolases are highly conserved along vast evolutionary distances [52], although some species-specific features/subunits are also seen. In contrast, we have described here a very unusual F-type ATP synthase in *T. thermophila*. The overall structure of this complex determined by single particle electron microscopy projections is dramatically different from any other ATP synthase examined. In organisms as divergent as *E. coli*, *Saccharomyces cerevisiae*, *Polytomella*, and the cow *Bos taurus*, the overall structures of ATP synthases are very similar [37],[53]. The most obvious difference in *T. thermophila* is the parallel disposition of individual ATP synthase monomers compared to the angular arrangement seen in all other organisms. It has recently been suggested that the angular arrangement of ATP synthases in mitochondria may be important for the curvature of the cristae tips formed by the inner mitochondrial membrane [54]. *Tetrahymena* (as well as other alveolate) mitochondria, however, have tubular cristae that do not form the curved tips seen in mitochondria from other organisms [55]. We suggest it as a possibility that the parallel arrangements of the ATP synthase monomers might dictate tubular cristae arrangement in ciliate (and perhaps in all alveolate) mitochondria.

A second unusual structural feature of the ATP synthase dimer is the presence of novel additional membrane-embedded domains that flank the dimer, and could be connected to the F_1_ headpiece. Such structures have not been observed in any ATP synthase thus far. This is in contrast to all other F-type and V-type ATPases. All studied F-type ATP synthases have just one stator, attached to subunit a. V-type ATP synthases have two to three stators [56],[57], but they merge together at one point where they connect to the c subunits ring via subunit a.

A third unusual feature is the presence of a large domain attached to the intermembrane part of one of the monomers. Again, this has not been observed in any ATP synthase and the significance of which is unclear. Remarkably, prokaryotic ATP synthases are structurally more similar to their mitochondrial counterparts than is *T. thermophila* mitochondrial ATP synthase.

### Subunit Proteins of ATP Synthase

We were able to resolve the large mitochondrial complexes through 2-D BN/BN-PAGE, which permitted a proteomic cataloguing of the subunit proteins that constitute *T. thermophila* ATP synthase. While it is possible that the proteomic analysis may have missed some of the component proteins, those that we did detect could be assigned with a reasonable degree of confidence as being subunits of the complex. Of the 24 proteins present in spot 1, 22 are the likely constituents of the ATP synthase. Only six of these were annotated as subunits of ATP synthase. On the basis of our analysis, two hypothetical proteins could be assigned as subunit d and δ; that leaves 14 proteins with no assigned functions, one of which has homologues believed to be oxidoreductases in many other organisms. Thus, 13 proteins that seem to be part of mitochondrial ATP synthase complex in *T. thermophila* have no detectable orthologs in any organism other than ciliates. The proteomic data and even phylogenetic analyses of generally conserved subunits seem to confirm the notion from structural studies that ciliate ATP synthase is highly divergent from its mitochondrial or bacterial counterparts. This degree of divergence is also apparent when one examines ciliate mitochondrial DNA. The 47-kb mtDNA in *Tetrahymena* encodes 44 ORFs, 20 of which have no orthologs in any organisms other than ciliates and have no function assigned to them [43],[58],[59]. To put this in perspective, the protozoan *Reclinomonas americana* has 67 mitochondrial ORFs, the largest number known thus far, of which 66 have orthologs in other species with assigned functions [60]. This finding would suggest either that the unassigned ORFs in ciliates have undergone highly accelerated evolutionary divergence or that ciliate mtDNAs have acquired almost half of their genes from sources other than the α-proteobacterial ancestral endosymbiont that lies at the origin of all extant mitochondria. Our extensive sequence searches have failed to find homologous sequences to the unassigned ciliate mtDNA ORF proteins or the 12 nuclearly encoded subunits of *T. thermophila* ATP synthase in any of the currently available collections of ORFs, which include metagenomes as well as Genomic Encyclopedia of Bacteria and Archaea Genomes available at the Joint Genome Institute. Thus, the provenance of these ciliate-specific mitochondrial proteins remains obscure.

### Proposed Candidates for F_o_ Subunits a and b

A major motivation for our study was the apparent lack of a gene encoding the subunit a of the F_o_ sector in complete genomic sequences of any alveolate. Because this subunit, in association with the multimeric subunit c ring, forms the channel through which protons move and drive the catalytic rotation of the enzyme, its absence would be incompatible with proton motive force driven ATP synthesis. Through proteomic analysis of isolated ATP synthase complexes and careful sequence comparison we now propose that the function of subunit a could be served by the highly divergent or novel protein Ymf66 encoded by the mtDNA. This protein is predicted to have eight transmembrane helices, one of which has buried arginines in positions where they could form critical residues for the proton channel as reported in other ATP synthases. Other than this tenuous but potentially critical homology, Ymf66 bears no discernable similarities to any known subunit a from any organism, except for the fact that, like most other subunits a, it too is encoded by mtDNA and is predicted to be a polytopic membrane protein. Remarkably, as discussed above, Ymf66 has no discernable ortholog in any organism other than ciliates.

Genes encoding the F_o_ subunit b were also not detected in any alveolate. Subunit b forms a crucial part of the stator that extends from the membrane to near the top of the globular F_1_ sector. The role of the stator is to stabilize α_3_β_3_ from rotation caused by the centrally positioned γ stalk. Again, the absence of a stator would be incompatible with ATP synthase function. Single particle electron microscopy projections, however, revealed the presence of not one but two stator structures in *T. thermophila* ATP synthase. Taking into consideration the requirement that subunit b has its N-terminal sequence buried in the membrane and rest of its amino acids forming extended mostly hydrophilic α-helical structure, we have identified three proteins detected in *T. thermophila* ATP synthase as candidate substitutes for the b subunit. It is not uncommon to have the stator structure formed by homo- or heterodimers of b subunits. Again, it was not possible to detect homologues of these proteins in any organisms other than ciliates.

### Implications for Other Alveolates

Dinoflagellates and Apicomplexa are two sister clades of ciliates that form the crown group alveolates. Therefore, it is intriguing that these related organisms seem to lack any of the unassigned proteins that are part of the ATP synthase complex in *T. thermophila*. Mitochondrial evolution in alveolates, however, is complicated [61]. Unlike the ∼44 ORFs encoded by the ciliate mtDNA [43],[58],[59], dinoflagellate and apicomplexan mtDNAs encode just three proteins [61],[62]. The massive loss of ORFs is also accompanied by unusual structural arrangements of mtDNA and scrambling of rRNA genes; some apicomplexans have actually lost the mitochondrial genome altogether [63],[64]. However, all these organisms, including those without mtDNA, continue to encode at least α and β subunits of ATP synthase. It is not clear whether these proteins are assembled into a functional ATP synthase, but there are indications that mitochondria are capable of ATP synthesis in at least some of apicomplexans. The question as to what constitutes the functional ATP synthase in these organisms remains unanswered. If what we have reported here for the ciliates is an indication, answers to this question could prove interesting and important, for Apicomplexan pathogens extract an enormous toll from humanity. The unusual and highly divergent ATP synthases could form attractive targets for selective therapeutic approaches.

## Materials and Methods

### Growth of *T. thermophila* (SB 210) and Isolation of Mitochondria

*T. thermophila* SB 210 cells were grown in proteose peptone media and mitochondria were isolated as previously described [19],[65],[66]. Briefly, 500 ml cultures were harvested at late log phase of growth by centrifugation at 1,000*g* for 5 min. The cells were washed with mitochondria isolation buffer (MIB; 0.3 M sucrose, 1 mM EDTA, 0.1mM EGTA, and 12.5 mM HEPES (KOH [pH 7.4]); trehalose was substituted for sucrose on two occasions with no evident changes in properties of the mitochondrial preparation), and were resuspended in 5 volume of MIB. The suspension was homogenized in a 30 ml Kontes tight fitting glass hand homogenizer on ice until 80%–90% of the cells were broken. The whole homogenate was transferred to a 50-ml conical tube and centrifuged at 300*g* for 5 min at 4°C in an HS-4 Sorvall rotor. The supernatant was centrifuged at 7,000*g* for 10 min at 4°C. The resulting fraction consists of a hard brown pellet at the bottom followed by cream-colored layer of mitochondria and a loose whitish layer above it. The supernatant and most of the whitish layer was carefully removed. Five volume of MIB was added gently to the pellet and gently shaken to remove the creamy mitochondrial layer. The crude mitochondrial fraction was resuspended in 10 ml of MIB containing 10% percoll and was centrifuged at 5,300*g* for 5 min. The supernatant was removed and the pellet was washed with 10% percoll again. To remove Percoll, the pellet was washed with MIB and centrifuged at 5,300*g* for 5 min at 4°C. The resulting pellet was resuspended with 1.5 ml of MIB and was layered on top of a discontinuous sucrose gradient (3 ml of 30% [w/v], 3 ml of 45%, and 3 ml of 60% sucrose) and was centrifuged at 22,000 rpm for 2 h at 4°C in a Sorvall SW27 rotor. A cream-colored band formed at about the position of the 45%–60% sucrose junction and was collected as the purified mitochondrial fraction. This fraction was resuspended in 10 ml of MIB and was centrifuged at 5,300*g* for 5 min to remove excess sucrose. The step was repeated again, and the final pellet was resuspended in a small volume of MIB buffer. Protein concentration was estimated by Bradford assay.

### 1-D BN-PAGE, ATPase Activity, and Electroelution

Mitochondria (1 mg protein) were resuspended in water and pelleted by centrifugation at 10,000 rpm for 10 min at 4°C in a Sorvall SW 50.1 rotor. The pellet was resuspended in mitochondria solubilization buffer (50 mM Nacl, 50 mM Imidazole/HCl [pH 7.0], 2 mM 6-aminohexanoic acid, and 1 mM EDTA, at 4°C). Detergent concentrations were adjusted to 5 µg digitonin per µg of mitochondrial protein, or 1.5 µg dodecyl maltoside per µg of mitochondrial protein by addition of 20% stock solutions of the respective detergent. After incubation for 30 min on ice, the sample was centrifuged for 30 min at 30,000 rpm in the SW 50.1 Sorvall rotor. Coomassie dye from a 5% G-250 stock suspension was added to the supernatant to give a detergent/dye ratio of 8. The sample was loaded in a 3%–10% BN-PAGE gradient gel and the gel was run for 3–4 h with an initial constant voltage of 100 V, followed by a constant current of 15 mA, as described by Wittig et al. [67].

In-gel ATPase activity of the enzyme was measured by incubating the BN gel strips in a buffer containing 35 mM Tris.HCl (pH 8.4), 270 mM glycine, 14 mM MgSO_4_, 0.2% Pb(NO_3_)_2_, and 4 mM ATP at room temperature for overnight as described [68].

For electroelution, protein complexes from the bands were cut with scalpel and transferred into electroeluter chambers (D-Tube Dialyzer, Novagen). The dialyzer tubes were pretreated with 1% ethanolamine and rinsed with ultrapure H_2_O. Electroelution was done overnight in the electroelution buffer (25 mM tricine, 7.5 mM Bis-Tris, 1 mM phenylmethylsulfonyl fluoride [pH 7.0]) containing either 0.1% digitonin or 0.03% dodecyl maltoside at 150 V in 4°C as described [69].

### 2-D BN-PAGE

2-D BN/BN-PAGE was carried out as described by Sunderhaus et al. [70] with slight modifications. The 1-D gel strip was incubated with 0.03% dodecyl maltoside (Anatrace) for 10 min. After incubation, the gel strip was placed in between the glass plates and a 4%–12% gradient gel was poured. After polymerization, the space between the 1-D gel strip and 4%–12% gradient gel was filled with a 3.5% stacking gel. Dodecyl maltoside to a final concentration of 0.03% was added in the cathode buffer and the gel was run overnight at a constant current of 15 mA.

### Respirometry of Permeabilized *T. thermophila* Cells

The relative amount of digitonin required to permeabilize 99% of freshly harvested cells was determined immediately prior to the experiment by monitoring loss of trypan blue exclusion after a 5 min incubation of cells suspended in MIB plus digitonin, and was found to be 0.135 mg digitonin per mg cellular protein. Cells containing 315 mg protein were incubated for 5 min. with digitonin under the above conditions, then diluted 6-fold with MIB, recovered by centrifugation, washed once more with MIB, and resuspended at ∼15 mg/ml. Oxygen consumption by the permeabilized cells was measured with a microcathode oxygen electrode (number 1302, Strathkelvin Instruments) in a closed respirometry cell (MT200, Strathkelvin Instruments) with a 100-μl working volume maintained at 32°C. The system was calibrated the same day as each experiment per the manufacturer's instructions. The working solution was MIB containing 2 mM magnesium chloride and 2 mM potassium phosphate with additions as indicated in the figure caption.

### Spectrophotometric ATP Hydrolase Assay

ATPase activity was determined using a coupled assay modified from Pullman et al. [71], in which NADH oxidation is coupled to ATP hydrolysis using lactate dehydrogenase and pyruvate kinase. The assay was performed at 35°C in a stirred cuvette with a final volume of 1 ml containing 50 mM HEPES (KOH [pH 7.5]), 2 mM MgSO_4_, 3 mM phosphoenolpyruvate, 0.3 mM NADH, four units lactate dehydrogenase (Sigma), four units pyruvate kinase (Sigma), 0.6 mg/ml dodecylmaltoside, ∼200 µg protein of mitochondrial preparation and including inhibitors of adenylate kinase (10 µM P1,P5-di(adenosine-5′) pentaphosphate and 5 mM AMP), vacuolar ATPase (0.2 µM concanamycin A), complex IV (2 mM KCN), and complex I (34 µM rotenone). The oxidation of NADH was recorded with a modified SLM-AMINCO DW2C dual wavelength spectrophotometer (On-Line Instrument Systems, Inc.) in dual mode (341–401 nm). Dual wavelength spectroscopy ameliorates the effects of light scattering with turbid samples. The specific ATPase activity was quantitated by measuring the slopes of the linear postactivation (steepest) part of the assay traces.

### Single Particle Electron Microscopy and Image Analysis

Electroeluted complexes were applied on carbon coated copper grids and negatively stained with 2% uranyl acetate by droplet method. Images were recorded on a CM12 electron microscope (Philips) operated at 120 kV with slow scan 4 k×4 k CCD camera (Gatan) at 78,000 magnification and pixel size 3.8 Å at the level of specimen. Single particle analysis was performed with the Groningen Image Processing (GRIP) software package as described by Dudkina et al. [35],[40].

### Proteomic Analysis

Bands or spots were excised from BN-PAGE gels and processed with either trypsin or chymotrypsin according to the Coomassie stained gel protocol described by Gundry et al. [72]. All samples were desalted with C^18^ Omix tips (Varian) according to manufacture's protocol. Peptides were analyzed using the LTQ (ThermoFinnigan) in gradient mode with the following gradients; 8.5%–30% of 0.1% formic acid/90% acetonitrile (30 min), 60% of 0.1% formic acid/90% acetonitrile (18 min), and to 100% of 0.1% formic acid/90% acetonitrile (22 min) with a flow rate of 300 nl/min. The peptides were separated on a hand-packed 75-µm reversed phase column consisting of YMC ODS-AQ (5-µm particle size and 120-A pore size). Using an electrospray voltage of 2.2 kV, precursor scans were taken from m/z of 350–1,800 m/z and the top eight ions picked for MS/MS.

The acquired MS/MS data were searched with Sorcerer 2-Sequest (SageN Research Products), with postsearch analysis using Scaffold (Proteome Software). Peak extraction was performed using Sorcerer 2 SEQUEST default settings. Data were searched using all species in the Trembl and National Center for Biotechnology Information (NCBI) databases as well as in the custom Smith et al. database [42]. The following criteria were used: a full trypsin or full chymotrypsin digestion, all species, and the variable modifications of carbamidomethyl and oxidation (methionine). Peptide mass tolerance was set to 1.2 amu. All MS/MS spectra were manually examined using Scaffold and low quality spectra were removed. Protein redundancy was then removed by using the Blast tool to assess protein similarity.

### Phylogenetic Analysis

Sequences of representative species from a broad range of eukaryotic groups were collected from the NCBI refseq protein database for most of the ATP synthase subunits that were identified in *T. thermophila*. In a few cases, the sequence set was extended with one or two translations of complimentary DNA (cDNA)/expressed sequence tag data (see Table S5); in these instances, we verified that the sequences used matched the relevant genomic data or were highly similar to sequence data from closely related species. Identifications of sequences used for alignment are given in Table S5. Sequences were aligned using ClustalX [73], TCoffee (Expresso) [74], and MAFFT (L-INS and/or E-INS strategy) [75]; the alignments were compared and unambiguously aligned positions chosen for phylogenetic analyses. MrBayes [76] was used for Bayesian inference [77] simulations. The program was run with two chains for at least 1.2 million generations, sampled every 60 generations, and analysis continued if necessary until probable convergence was indicated by stability of the log likelihood values and the standard deviation of split frequencies for at least 0.6 million generations. A preliminary run using the “mixed” amino acid model was used to find the optimal amino acid model, which was the “WAG” model [78] with our datasets, and the final analyses were run using the WAG model and assuming invariable positions and a gamma-distributed substitution rate heterogeneity [79], the “WAG+Γ+I” model. Probable convergence was verified postsimulation by the randomness of the plot of log likelihood values and potential scale reduction factor (PSRF) values of 1.00. PhyML [80] was used for maximum likelihood phylogenetic analysis [81] using the WAG+Γ+I model and calculating support with 200 nonparametric bootstrap repeat calculations (using α and proportion invariant parameters fixed at the values optimized for the real data to minimize computation time). Phylogenetic tree output was viewed and arranged for presentation using the Tree Explorer module in the MEGA 4 package [82].

### Protein Sequence Searches and Analyses

To attempt to identify homologies for the unassigned protein sequences discovered in spot 1 from 2-D BN-PAGE by the LC/LC/MS analysis, comparative searches were conducted using multiple algorithms and protein databases: (1) BLAST search [83],[84] repeated with all three available BLOSUM amino acid matrices at NCBI databases; also repeated at the CAMERA metagenomic database [85] available at http://camera.calit2.net/. (2) SSEARCH at EBI (http://www.ebi.ac.uk/Tools/fasta33/index.html?program=SSEARCH), which conducts a rigorous Smith-Waterman search [86]. (3) Sequence search at Pfam database version 23 and 24 [87], which is based on the HMMER hidden Markov model program. (4) COMPASS, a generalized Psi-BLAST alignment profile to alignment profile query [88] available at http://prodata.swmed.edu/compass/compass.php.); for this purpose, the *T. thermophila* proteins were aligned with their orthologs from *P. tetraurelia* (and other ciliates if available).

Significant similarities found are indicated in Tables 2 and S4, but in the majority of cases, no additional significant matches were obtained. Texts S1 and S2 contain detailed examples of the results of many of these searches.

The prediction of transmembrane helices in membrane proteins was carried out using TMHMM (v2) (http://www.cbs.dtu.dk/services/TMHMM/), TMMOD (http://liao.cis.udel.edu/website/servers/TMMOD/scripts/frame.php?p=submit), and TOPCONS (http://topcons.cbr.su.se/index.php) [89]–[92]. Protein secondary structure predictions were made using the PSIPRED Protein Structure Prediction Server (http://bioinf.cs.ucl.ac.uk/psipred/) [93],[94].

## Supporting Information

Figure S1 **Typical reaction traces of** ***Tetrahymena*** **ATPase activity.** The activity was measured by a coupled spectrophotometric assay (Materials and Methods), minus and plus high concentrations of inhibitors (20 µM oligomycin and 75 mM sodium azide). Note the time-dependent increase in the rate of ATP hydrolysis. The yellow overlay box on the first trace indicates the approximate range of the linear, steady state enzyme activity attained after activation.
(1.23 MB TIF)

Figure S2 **Projections of I–III_2_ supercomplex and complex III_2_ from** ***Tetrahymena thermophila*** **(A–D) and** ***A. thaliana*** **(E).** (A) Average of 2,217 projections of I–III_2_ supercomplex representing the side view. (B) Average of 1,657 projections of the top view of I–III_2_ supercomplex. (C) Top view of I–III_2_ supercomplex lacking the NADH-oxidizing domain of complex I (sum of 512 projections). (D) Average of 768 projections of complex III_2_ in side view position. (E) Projection of dimeric complex III from *Arabidopsis* in a similar orientation [40]. White arrowheads mark the core 1 and 2 subunits of complex III_2_ in frames (A) and (D), and black arrowheads point to the presence (B) or absence (C) of the peripheral arm of complex I. All data are results from combined data of proteins extracted with digitonin and those extracted with dodecyl maltoside except for complex III_2_, which was solubilized with digitonin. Bar = 10 nm.
(1.25 MB TIF)

Figure S3 **Ymf66 and ATP synthase subunit a transmembrane topology predictions and position of conserved Arg residue.** (A) Graphical representations of TM topology calculated by TMHMM v2. Red rectangles at the top of each plot delineate the predicted transmembrane regions. Darker red rectangles in the *E. coli* plot (A2) indicate the extent of TM helices estimated using the results of biochemical and molecular genetic studies (see [95] and references therein). An asterisk in each plot denotes the position of the conserved Arg residue in subunit a or of a possible corresponding Arg in ciliate Ymf66. Plots shown: A1, *T. thermophila* Ymf66; A2, *E. coli* F_o_ a; A3 *B. taurus* F_o_ a; A4, *A. thaliana* F_o_ a; A5, *R. americana* F_o_ a; A6, *T. brucei* F_o_ a. (B) Position of arginines in putative TM6 of Ymf66 and possible alignment with the region of ATP synthase subunits a that contains the conserved Arg (reproduction of Figure 4 for convenience). Residues that are identical to or chemically similar to the consensus amino acid are shown with reverse coloration, and the conserved Arg is shown in red and highlighted. The extent of known (*E. coli* subunit a) and predicted (Ymf66) TM helices is indicated with dark red bars.
(0.27 MB PDF)

Figure S4 **Secondary structure predictions for the C-terminal domains of putative** ***T. thermophila*** **ATP synthase subunits having hydrophobic segments in their N-terminal regions.** Secondary structure was predicted by the PSIPred server (http://bioinf.cs.ucl.ac.uk/psipred/) for the sequences shown. The TM predictions are shown to indicate the approximate location of hydrophobic sections in the full sequences. The prediction for bovine subunit b is shown for comparison.
(0.79 MB PDF)

Figure S5 **Phylogenetic tree inferred for subunit β by Bayesian analysis.** Numbers near branch nodes indicate Bayesian posterior probabilities/maximum likelihood bootstrap support (200 replicates). Branches with less than 0.5 posterior probability have been collapsed to a common node. The bar at the lower left indicates the scale in number of substitutions per site. Phylogenetic trees for subunits δ and d are shown in Texts S1 and S2, respectively.
(0.04 MB PDF)

Tabe S1 **Summary of proteins identified by mass spectrometric analysis of peptides in bands excised from BN-PAGE gels and spots excised from 2-D BN-PAGE gels.**
(0.11 MB XLS)

Table S2 **Sequences, MS parameters, and deduced parent proteins of peptides identified by LC/MS/MS analysis of bands excised from BN-PAGE gels.**
(0.27 MB XLS)

Table S3 **Sequences, MS parameters, and deduced parent proteins of peptides identified by LC/MS/MS analysis of spots excised from 2-D BN-PAGE gels.**
(0.37 MB XLS)

Table S4 ***T. thermophila*** **mitochondrial proteins identified in spots 1–5 excised from 2-D BN-PAGE.** The peptides detected by LC/MS/MS (Tables S1–S3) belonging to each of the 60 proteins detected are highlighted within their amino acid sequences.
(0.09 MB DOC)

Table S5 **gi identification numbers of proteins used in phylogenetic analyses.**
(0.04 MB XLS)

Text S1 **Supplemental results: proposed revision of gi:118355322/trembl:Q22ZH1/Hypothetical protein TTHERM_01094890 and identification as putative** ***T. thermophila*** **ATP synthase delta subunit.**
(0.79 MB PDF)

Text S2 **Supplemental results: proposed revision of gi:89295266/trembl:Q239R1/Hypothetical protein TTHERM_01188360 and identification as putative** ***T. thermophila*** **ATP synthase F_o_ d subunit.**
(2.17 MB PDF)

## Abbreviations

BN: blue native
LC/MS/MS: Liquid Chromatography-Mass Spectrometry-Mass Spectrometry also known as Liquid Chromatography-tandem Mass Spectrometry
MIB: mitochondria isolation buffer
mtDNA: mitochondrial DNA
ORF: open reading frame
PAGE: polyacrylamide gel electrophoresis
