# Supplementary figures and images for: Highly Divergent Mitochondrial ATP Synthase Complexes in Tetrahymena thermophila
Source: PLoS Biol. 2010 Jul 13;8(7):e1000418. doi: 10.1371/journal.pbio.1000418 (PMC2903591; doi:10.1371/journal.pbio.1000418)

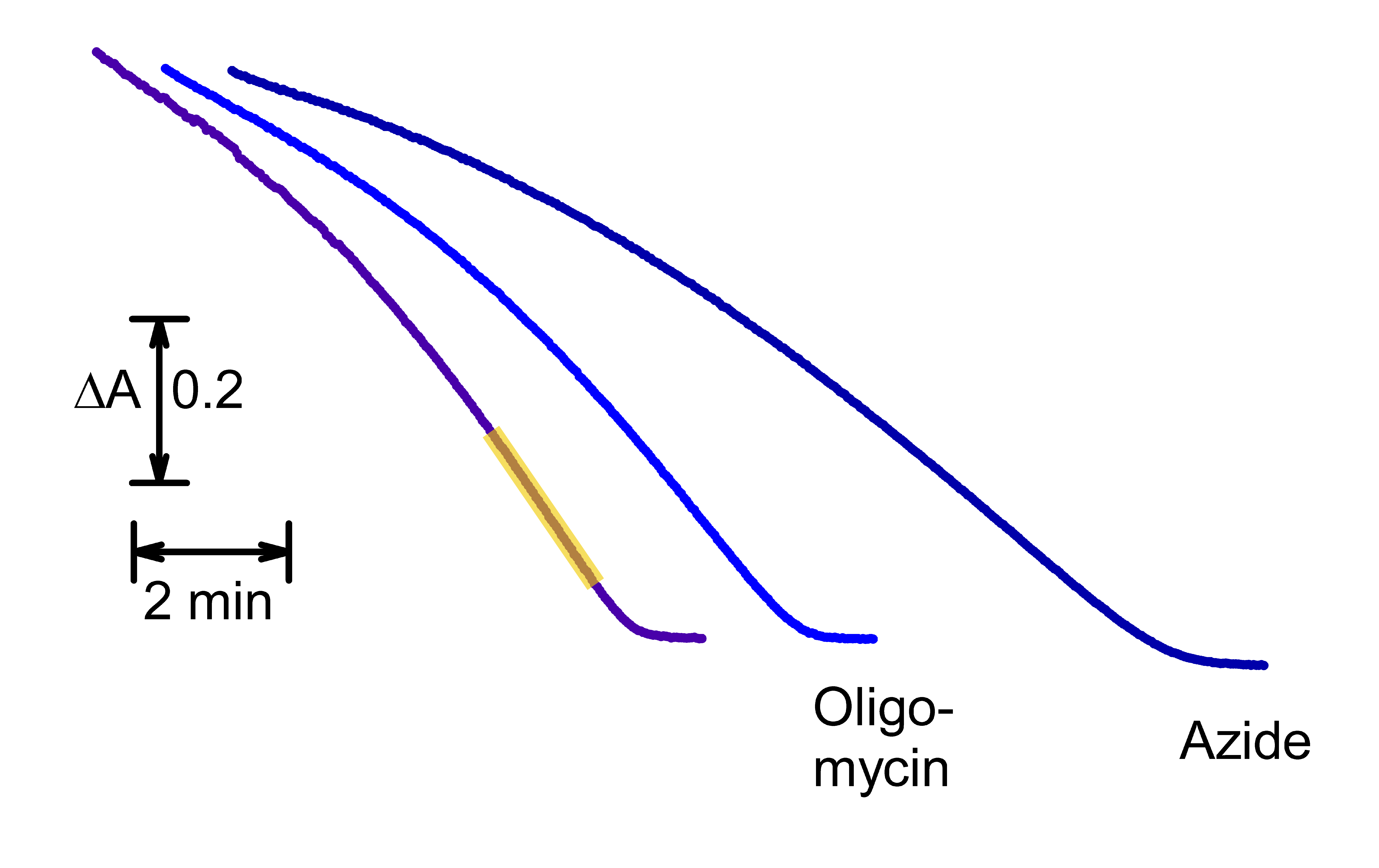

Supplement: Figure S1 — Typical reaction traces of Tetrahymena ATPase activity. The activity was measured by a coupled spectrophotometric assay (Materials and Methods), minus and plus high concentrations of inhibitors (20 µM oligomycin and 75 mM sodium azide). Note the time-dependent increase in the rate of ATP hydrolysis. The yellow overlay box on the first trace indicates the approximate range of the linear, steady state enzyme activity attained after activation. (1.23 MB TIF) [file pbio.1000418.s001.tif]

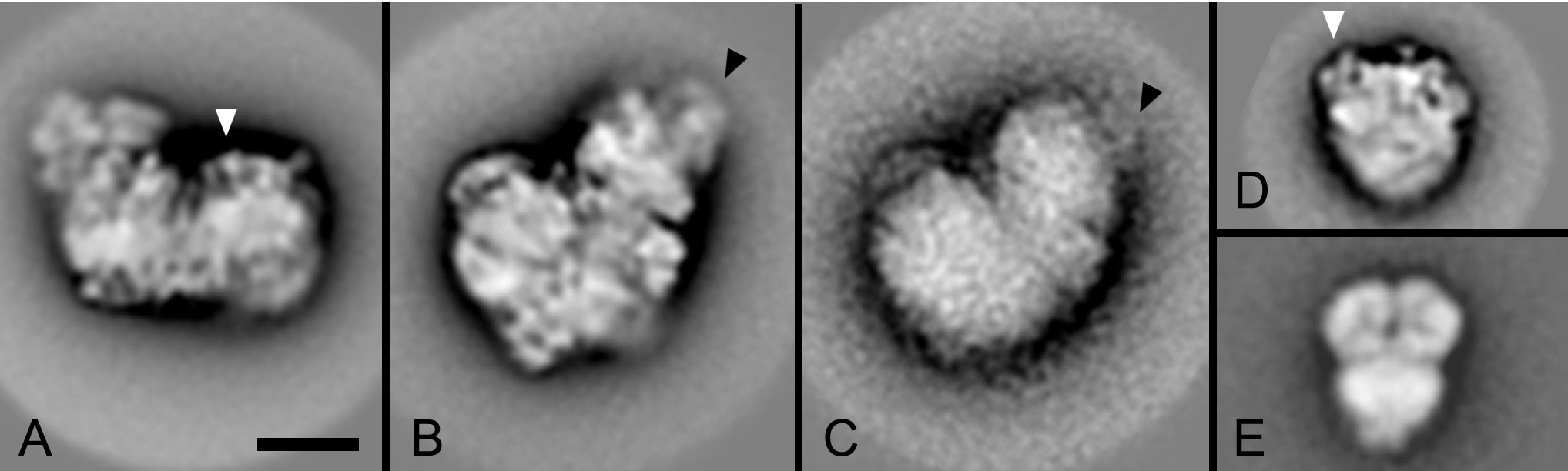

Supplement: Figure S2 — Projections of I–III2 supercomplex and complex III2 from Tetrahymena thermophila (A–D) and A. thaliana (E). (A) Average of 2,217 projections of I–III2 supercomplex representing the side view. (B) Average of 1,657 projections of the top view of I–III2 supercomplex. (C) Top view of I–III2 supercomplex lacking the NADH-oxidizing domain of complex I (sum of 512 projections). (D) Average of 768 projections of complex III2 in side view position. (E) Projection of dimeric complex III from Arabidopsis in a similar orientation [40]. White arrowheads mark the core 1 and 2 subunits of complex III2 in frames (A) and (D), and black arrowheads point to the presence (B) or absence (C) of the peripheral arm of complex I. All data are results from combined data of proteins extracted with digitonin and those extracted with dodecyl maltoside except for complex III2, which was solubilized with digitonin. Bar = 10 nm. (1.25 MB TIF) [file pbio.1000418.s002.tif]

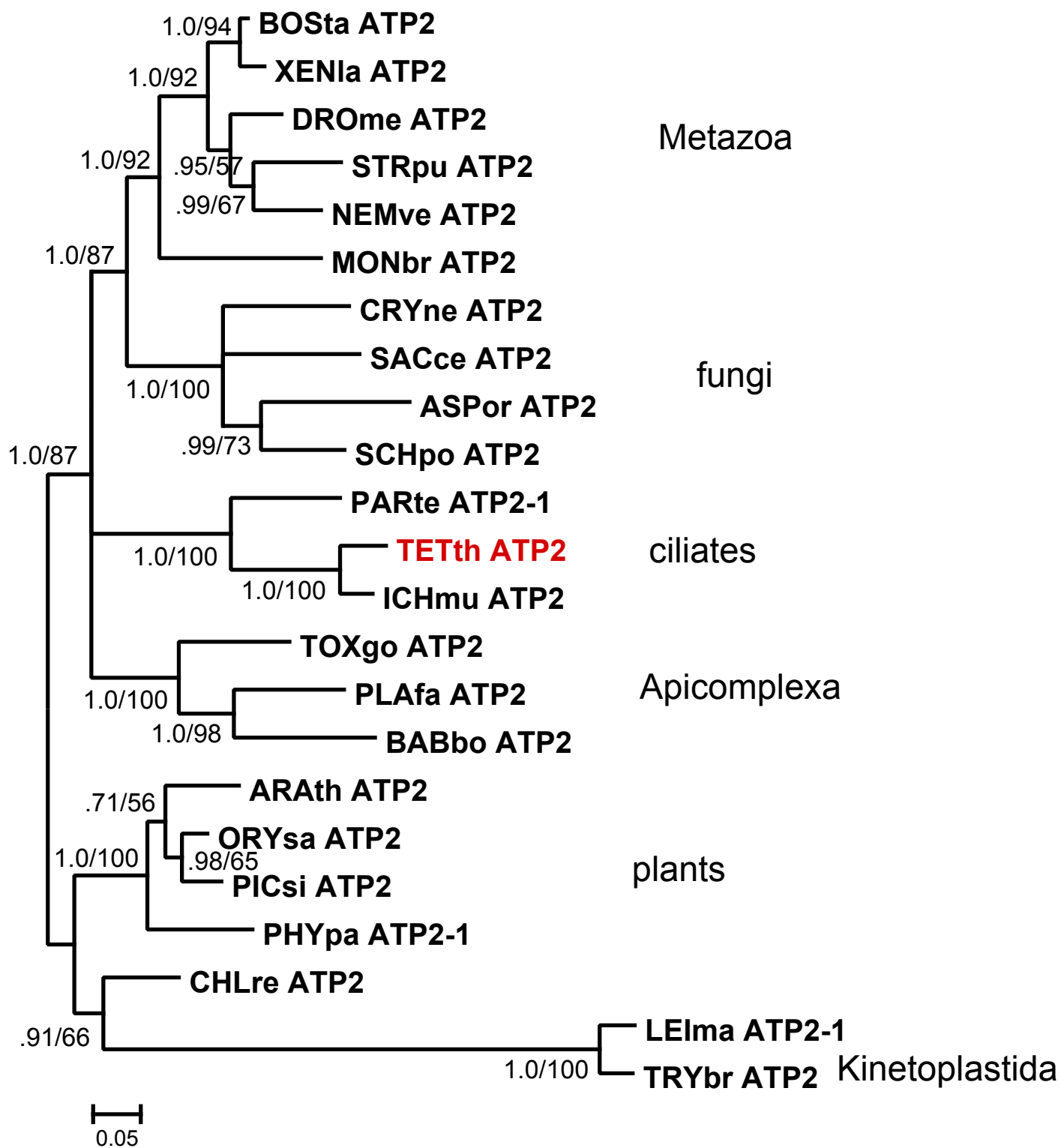

Supplement: Figure S5 — Phylogenetic tree inferred for subunit β by Bayesian analysis. Numbers near branch nodes indicate Bayesian posterior probabilities/maximum likelihood bootstrap support (200 replicates). Branches with less than 0.5 posterior probability have been collapsed to a common node. The bar at the lower left indicates the scale in number of substitutions per site. Phylogenetic trees for subunits δ and d are shown in Texts S1 and S2, respectively. (0.04 MB PDF) [file pbio.1000418.s005.pdf]
