# Supplementary material for: Highly Divergent Mitochondrial ATP Synthase Complexes in Tetrahymena thermophila
Source: PLoS Biol. 2010 Jul 13;8(7):e1000418. doi: 10.1371/journal.pbio.1000418 (PMC2903591; doi:10.1371/journal.pbio.1000418)
Supplement: Figure S3 — Ymf66 and ATP synthase subunit a transmembrane topology predictions and position of conserved Arg residue. (A) Graphical representations of TM topology calculated by TMHMM v2. Red rectangles at the top of each plot delineate the predicted transmembrane regions. Darker red rectangles in the E. coli plot (A2) indicate the extent of TM helices estimated using the results of biochemical and molecular genetic studies (see [95] and references therein). An asterisk in each plot denotes the position of the conserved Arg residue in subunit a or of a possible corresponding Arg in ciliate Ymf66. Plots shown: A1, T. thermophila Ymf66; A2, E. coli Fo a; A3 B. taurus Fo a; A4, A. thaliana Fo a; A5, R. americana Fo a; A6, T. brucei Fo a. (B) Position of arginines in putative TM6 of Ymf66 and possible alignment with the region of ATP synthase subunits a that contains the conserved Arg (reproduction of Figure 4 for convenience). Residues that are identical to or chemically similar to the consensus amino acid are shown with reverse coloration, and the conserved Arg is shown in red and highlighted. The extent of known (E. coli subunit a) and predicted (Ymf66) TM helices is indicated with dark red bars. (0.27 MB PDF) [file pbio.1000418.s003.pdf]

A

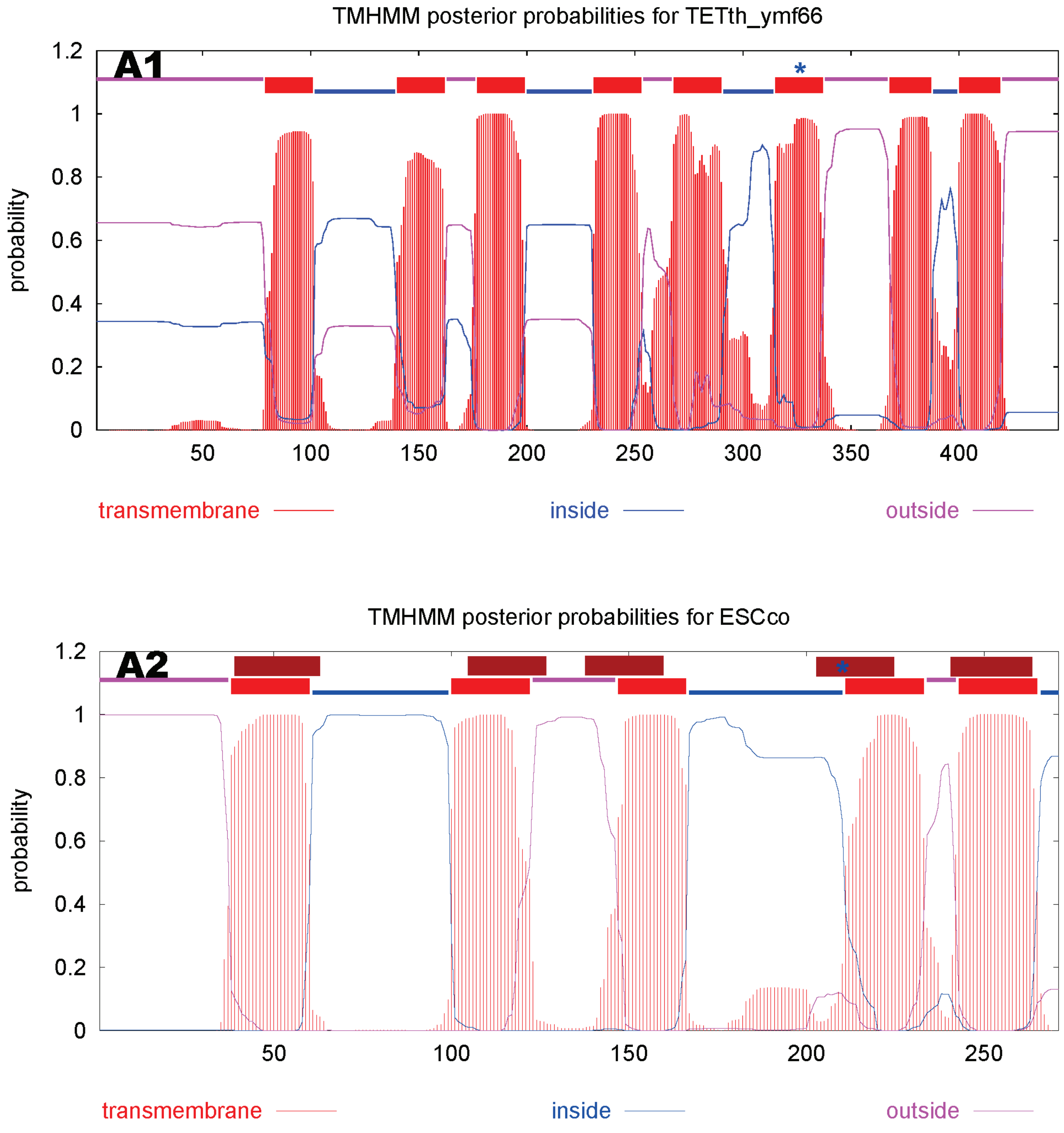
TMHMM posterior probabilities for TETth_ymf66
A1
*
1.2
1
0.8
0.6
0.4
0.2
0
probability
50
100
150
200
250
300
350
400
transmembrane
inside
outside
TMHMM posterior probabilities for ESCco
A2
*
1.2
1
0.8
0.6
0.4
0.2
0
probability
50
100
150
200
250
transmembrane
inside
outside

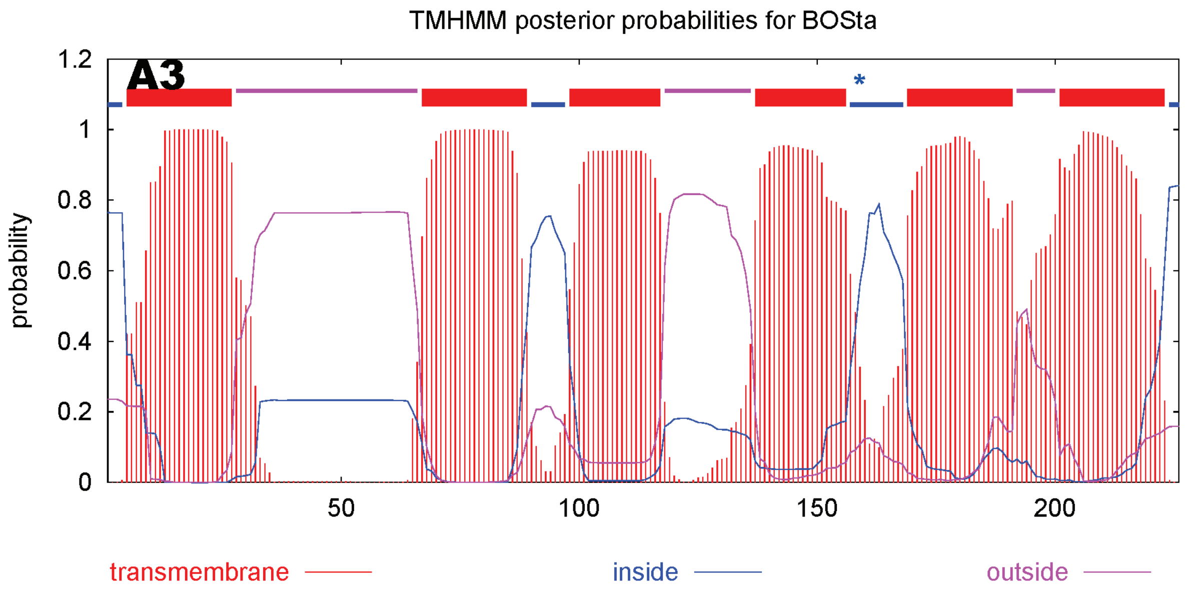
TMHMM posterior probabilities for BOSta
A3
probability
1.2
1
0.8
0.6
0.4
0.2
0
50
100
150
200
transmembrane
inside
outside

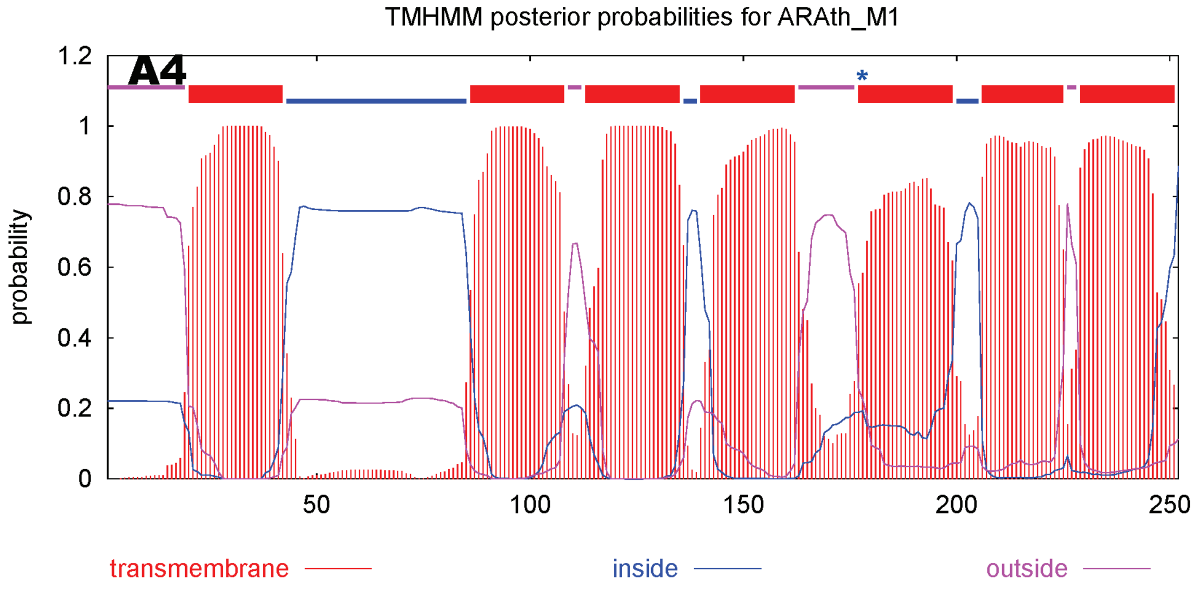
TMHMM posterior probabilities for ARAth_M1
A4
probability
1.2
1
0.8
0.6
0.4
0.2
0
50
100
150
200
250
transmembrane
inside
outside

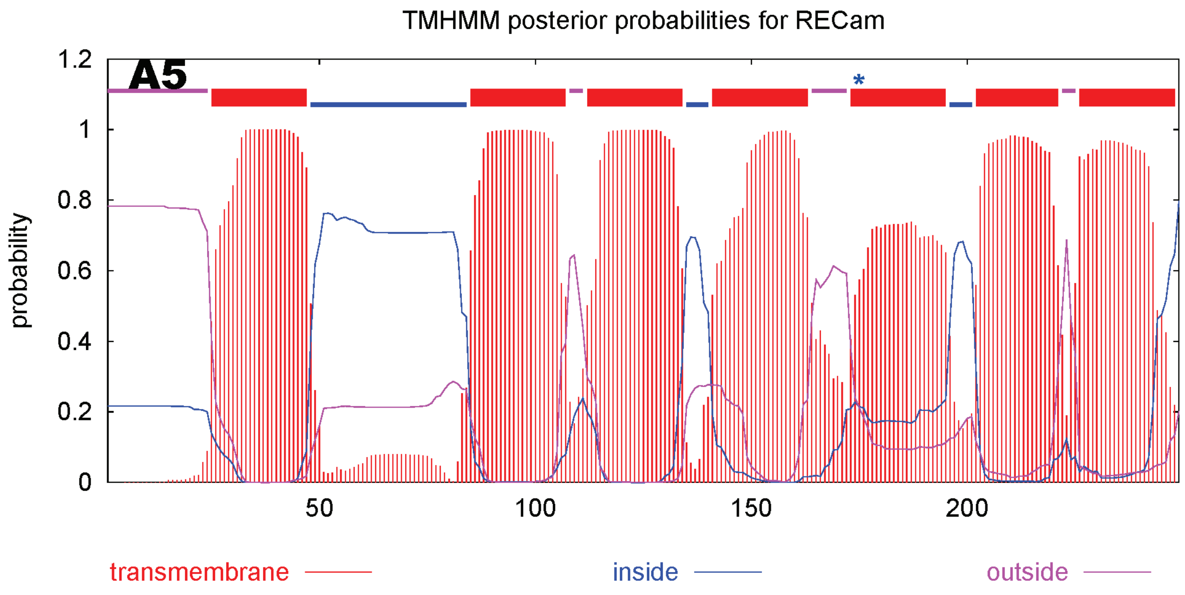
TMHMM posterior probabilities for RECam
A5
probability
1.2
1
0.8
0.6
0.4
0.2
0
50
100
150
200
transmembrane
inside
outside

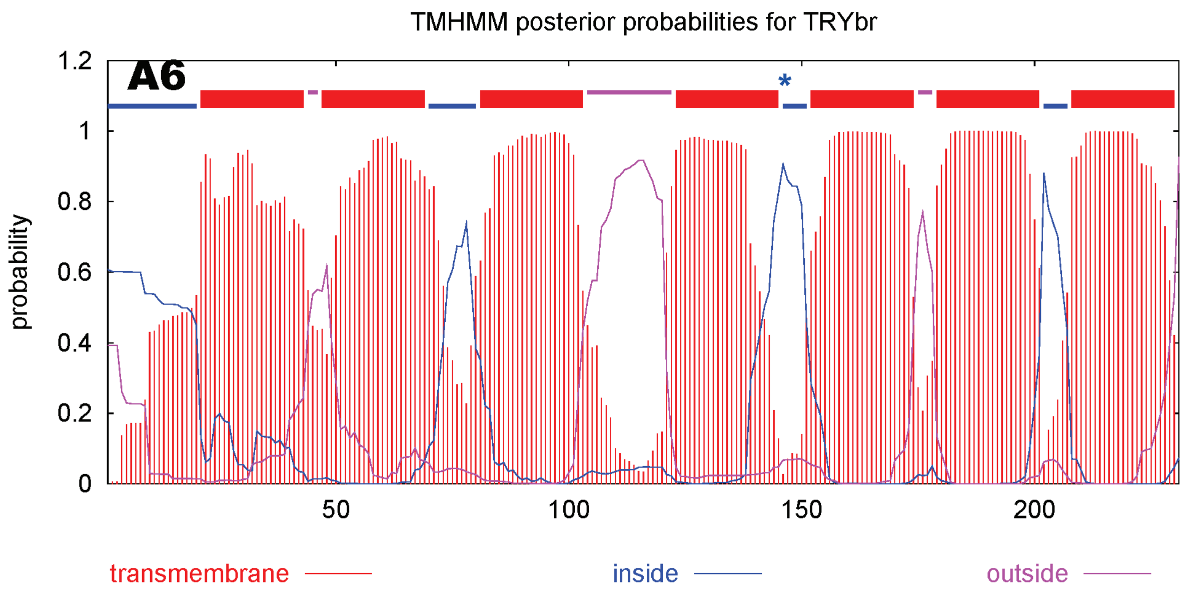
TMHMM posterior probabilities for TRYbr
A6
probability
1.2
1
0.8
0.6
0.4
0.2
0
50
100
150
200
transmembrane
inside
outside

B

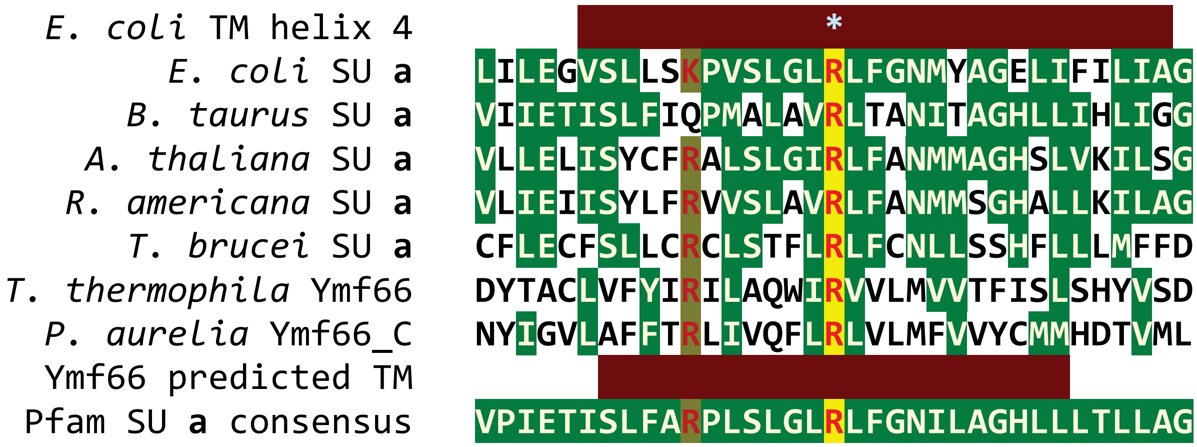
E. coli TM helix 4
*
E. coli SU a LILEGVSLLSKPVSLGLRLFGNMYAGELIFILIAG
B. taurus SU a VIIETISLFIQPMALAVRLTANITAGHLLIHLIGG
A. thaliana SU a VLLELISYCFRALSLGIRLFANMMAGHSLVKILSG
R. americana SU a VLIEIISYLFRVVSLAVRLFANMMSGHALLKILAG
T. brucei SU a CFLECFSLLCRCLSTFLRLFCNLLSSHFLLLMFFD
T. thermophila Ymf66 DYTACLVFYIRILAQWIRVVLMVVTFISLSHYVSD
P. aurelia Ymf66_C NYIGVLAFFTRLIVQFLRLVLMFVVYCMMHDTVML
Ymf66 predicted TM
Pfam SU a consensus VPIETISLFARPLSLGLRLFGNILAGHLLLTLLAG
