# Supplementary material for: Highly Divergent Mitochondrial ATP Synthase Complexes in Tetrahymena thermophila
Source: PLoS Biol. 2010 Jul 13;8(7):e1000418. doi: 10.1371/journal.pbio.1000418 (PMC2903591; doi:10.1371/journal.pbio.1000418)
Supplement: Figure S4 — Secondary structure predictions for the C-terminal domains of putative T. thermophila ATP synthase subunits having hydrophobic segments in their N-terminal regions. Secondary structure was predicted by the PSIPred server (http://bioinf.cs.ucl.ac.uk/psipred/) for the sequences shown. The TM predictions are shown to indicate the approximate location of hydrophobic sections in the full sequences. The prediction for bovine subunit b is shown for comparison. (0.79 MB PDF) [file pbio.1000418.s004.pdf]

A. Bovine subunit **b** prediction (for comparison).

Bos Taurus ATP synthase subunit **b** (gi|84490369) C-terminal domain

VKKYGASVGEFADKLNEQKIAQLEEVKQASIKQIQDAIDMEKSQQALVQKRHYLFDVQRN
NIAMALEVTYRERLHRVYREVKNRLDYHISVQNMMRQKEQEHMINWVEKRVVQSISAQQE
KETIAKCIADLKLLSKKAQAQPVM

PSIPRED PREDICTION RESULTS:

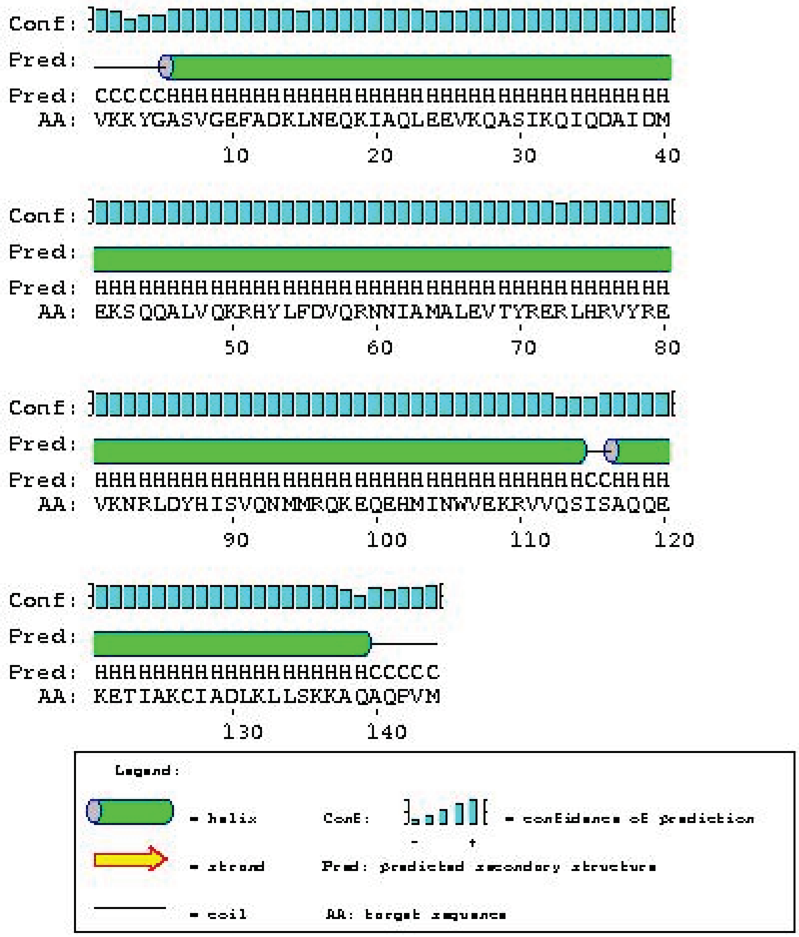

B. 146185889 hypothetical protein TTHERM_00529760 C-terminal domain
GDDNKCYSPDRFPYLKKRAGDMALPLNSLNQRTSAHYIEINAIYGAEMMKRYHKVWENIIEERSKATDQEKKTR

TMMOD TM prediction:

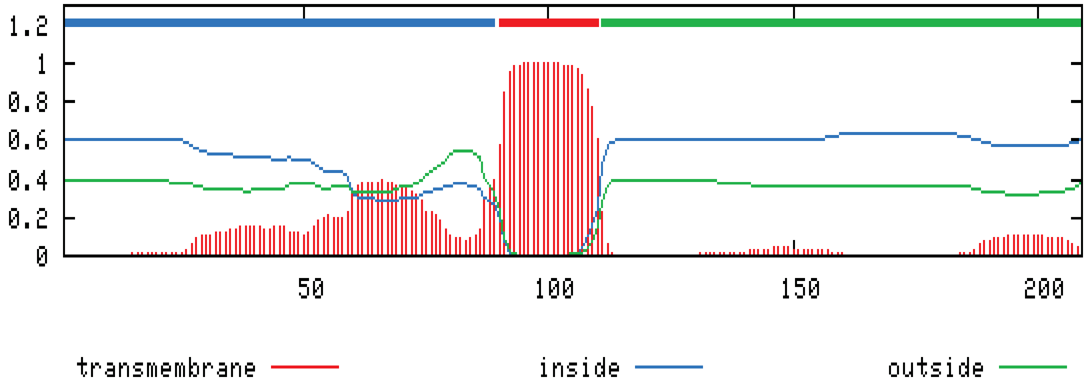

PSIPRED PREDICTION RESULTS:

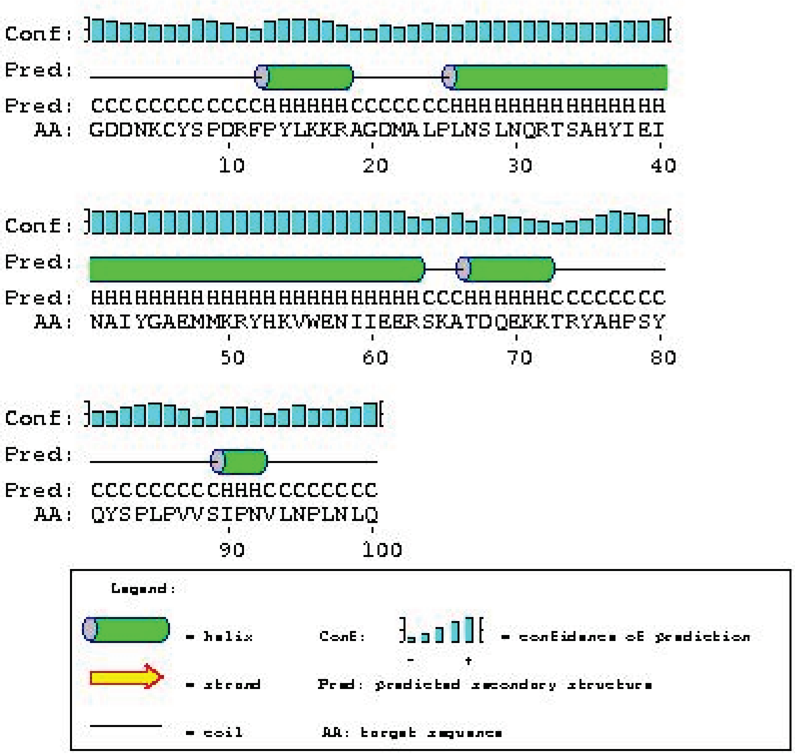

C. 118366175 hypothetical protein TTHERM_00127260 C-terminal domain
LLQRFYRRAAANEIFTMEAFYHENIENKLRNLMRITKGQLEYWDIHTSYGEIRADSINNFLANEYLRLQSHITS
RALNILKQAQAYETMNQAALLQKLIDDATSAIDNALKGDKKAEVLARSLDSAIDGLSKGYMDYQNDPLLPLILS
SIEANVKKITTLSAQEQANLIGLTAEQLKSIKENDVRARKEFLESQPKLDNNLKNIESVKKILATWGK

TMMOD TM prediction:

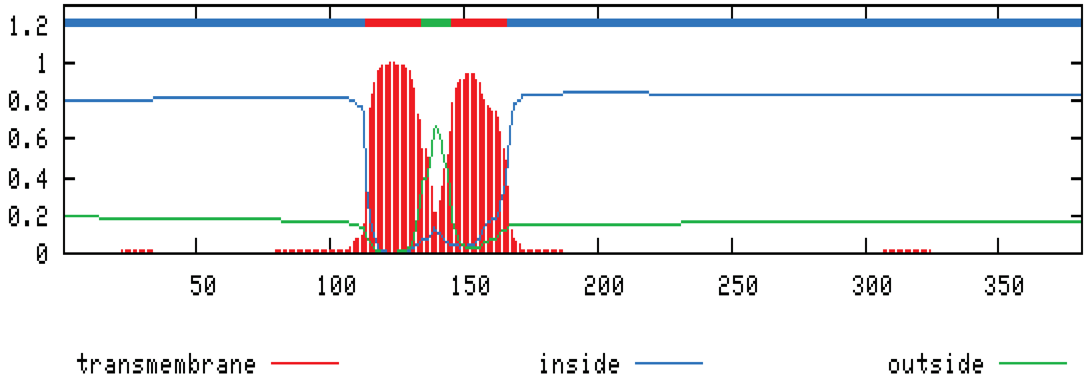

PSIPRED PREDICTION RESULTS:

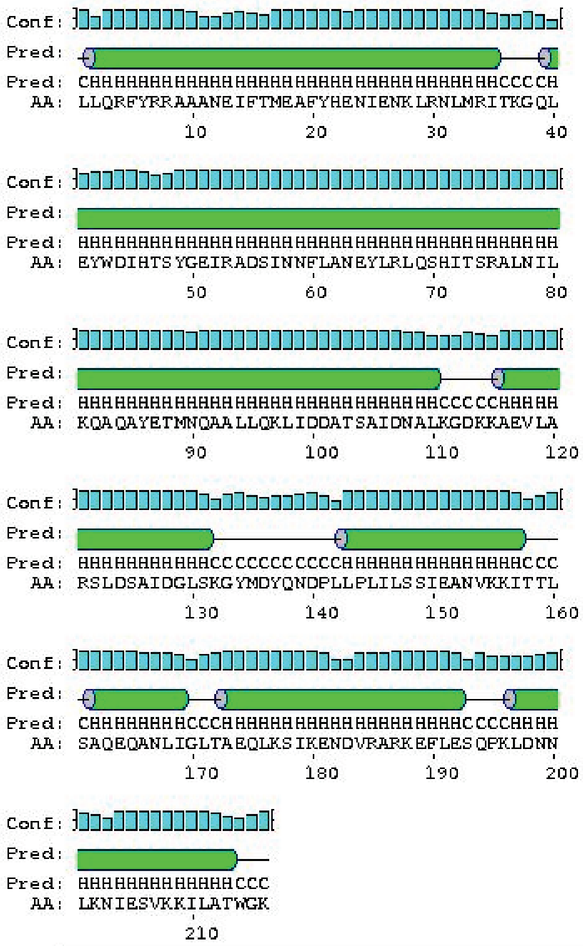

D. 146161614 hypothetical protein TTHERM_00068120 C-terminal domain
VDKYIFGENGNGGKFLEMQTINSNYDYYYNRQFQRMRYLTEDPAGDDPLQKTKDEHLVDLGFIPKVFGANVEVR
KRAPHDKYL

TMMOD TM prediction:

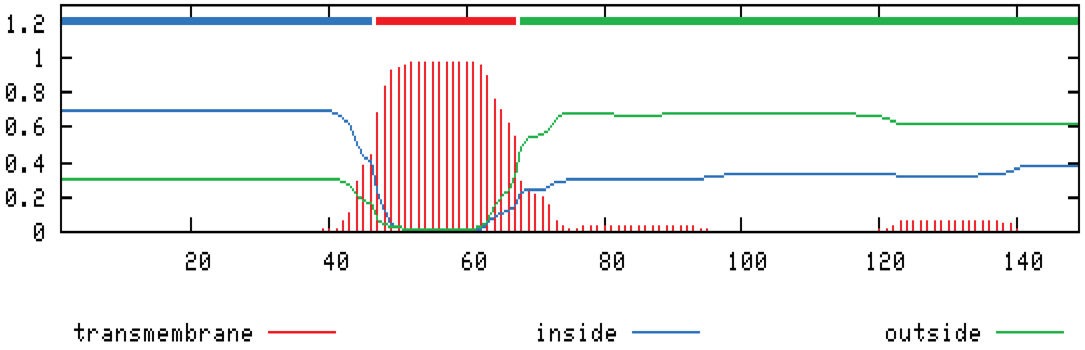

PSIPRED PREDICTION RESULTS:

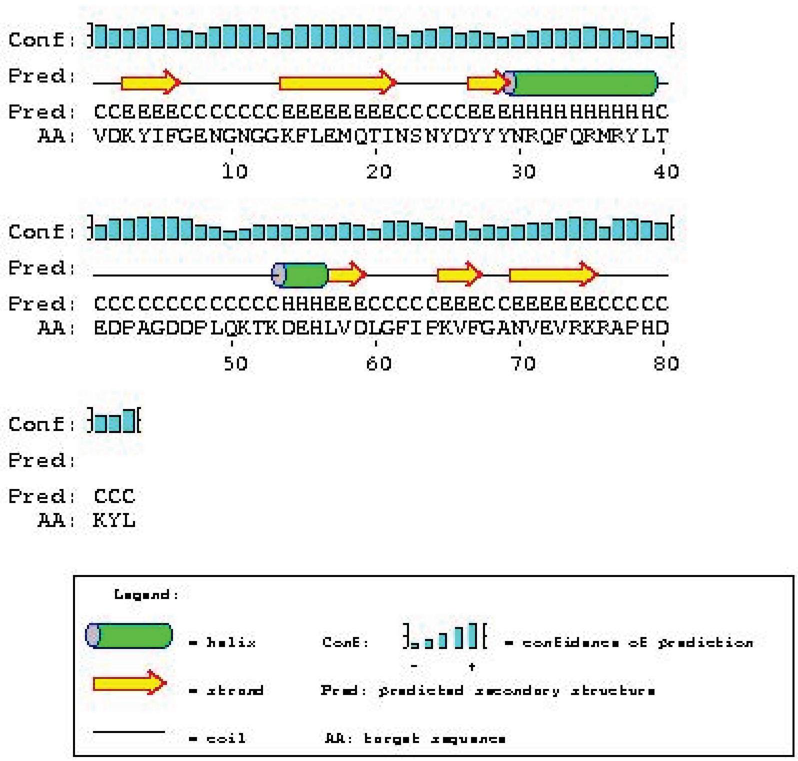

E. 146180703 hypothetical protein TTHERM_00316290 C-terminal domain
YRSQLVKVNLFDEYIQARAQELVKQNEYLLEHEDVKRYVWWYEDLKETLARVHRQANNHKACDFKDSEIILQDF
IRRYTNPKDNLPIKFHPQGQTF

TMMOD TM prediction:

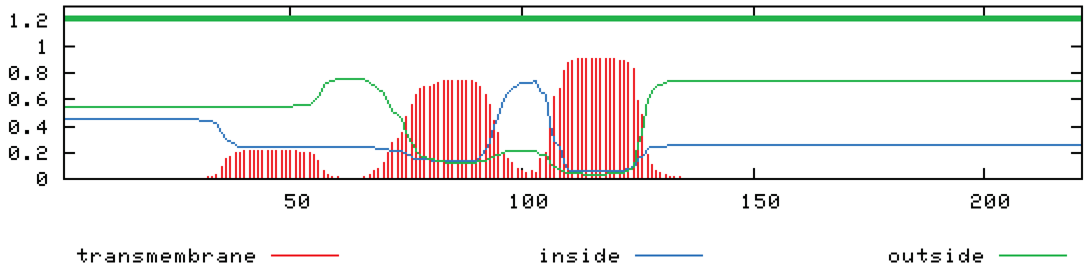

PSIPRED PREDICTION RESULTS:

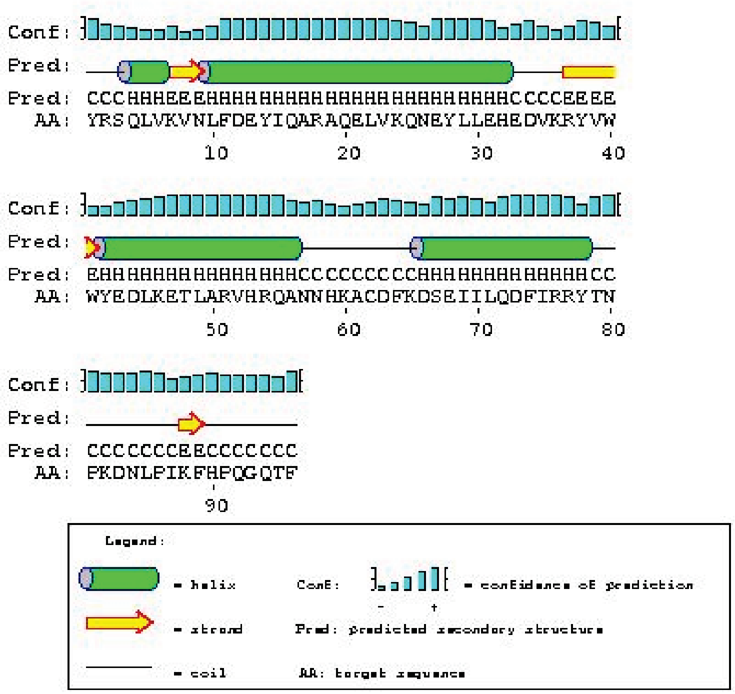

F. 118398278 hypothetical protein TTHERM_00823660 C-terminal domain
YGRYGYIINESSQKRAAQDLLDNDMAADKILFKNRVGAPTRPLRSLDDMMAFLSGSATYDQLADYASYNHAMDV
NQDQQAGLDSWMSEKDKNMVKYYQRSLGKKVEGI

TMMOD TM prediction:

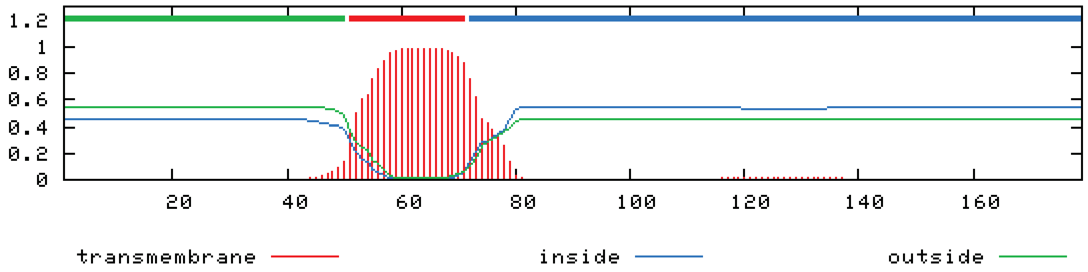

PSIPRED PREDICTION RESULTS:

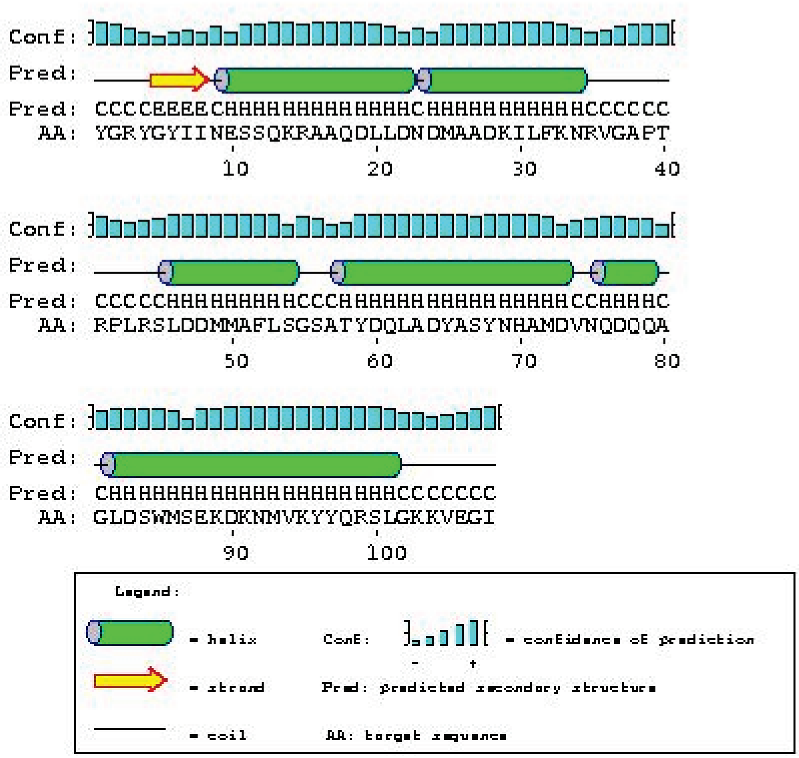

Figure S4. Secondary structure predictions for C-terminal domains of subunit **b** candidates. Servers used: PSIPRED Protein Structure Prediction -http://bioinf.cs.ucl.ac.uk/psipred/ ; TMMOD - http://liao.cis.udel.edu/website/servers/TMMOD/scripts/frame.php?p=submit.
