# Supplementary material for: Highly Divergent Mitochondrial ATP Synthase Complexes in Tetrahymena thermophila
Source: PLoS Biol. 2010 Jul 13;8(7):e1000418. doi: 10.1371/journal.pbio.1000418 (PMC2903591; doi:10.1371/journal.pbio.1000418)
Supplement: Table S4 — T. thermophila mitochondrial proteins identified in spots 1–5 excised from 2-D BN-PAGE. The peptides detected by LC/MS/MS (Tables S1–S3) belonging to each of the 60 proteins detected are highlighted within their amino acid sequences. (0.09 MB DOC) [file pbio.1000418.s009.doc]

**Table S4**

*T. thermophila* mitochondrial proteins identified in Spots 1-5 (Fig. 2C) excised from 2D BN PAGE. The peptides detected by LC/MS/MS (Tables S1-S3) belonging to each of the 60 proteins detected are highlighted within their amino acid sequences. The 24 proteins detected in Spot 1 are within the outlined box.

**Legend:**

ASTGEDSV light blue highlight = tryptic peptide

CVESLVA blue highlight = overlap of tryptic peptides

ALETDNVG yellow highlight = chymotryptic peptide

ASKQAVA dark red highlight = overlap of chymotryptic peptides

DAGEMVE teal highlight = overlap of chymotryptic with tryptic peptide

HNYIPGAK amino acid changes or additions due to a revised gene model

>gi:146184059 ATP synthase F_1_ alpha subunit (Atp1) (19 peptides)

MIRNFHSLVKRVPRLALTPFFGFRTSMTLADKKLSTGEASVVLAEKIKGITQQNDITEYGTVISIGDGIARVFGLTKVQAGEMVEFKSGIRGMALNLETDNVGVVVLGNDRDIKEGDVVKRTGAIVDVPIGEAMCGRVFDALGNPIDGLGPLKTTQRARVEIKAPGIIPRQSVRQPMQTGIKCVDSLVPIGRGQRELIIGDRQTGKTAIAIDTILNQKEAFNTGDVKKQLYCIYVAVGQKRSTIANLVSILKQHDCMKFTIVVCATASDAAPLQFLAPYSGCAIGEFFRDNGKHALIIYDDLSKQAVAYRQMSLLLRRPPGREAYPGDVFYLHSRLLERAAKMNDSLGGGSLTALPVIETQAGDVSAYIPTNVISITDGQIFLETELFYKGIRPAINVGLSVSRVGSAAQIKAMKKIAGNLKLTLATYRELAAFSQFGSDLDAKTQQQLNTGERLVEMLKQNQYTPMKVEEQVCIIFAGVKGFLDALVTSEVLKFEKKFLEHVRTNHSALLKRIRDSGDLSEVDTNELNTIIPLFIQEGGFKLKAQ

>gi:146185860 ATP synthase F_1_ beta subunit (Atp2) (19 peptides)

MLSKALQRGIARAFSTTAKKEAPKTVKANGQVSQVIGAVVDVQFEGELPQILNALEVQGTQHRLVLEVAQHLGDSRVRTIAMDSTEGLVRGQPVVDTGLPISVPVGPGTLGRIMNVIGEPIDQRGPIKAAKLYPIHRDAPSFTDQATSAEILVTGIKVVDLLAPYARGGKIGLFGGAGVGKTVLIQELINNVAKHHGGYSVFAGVGERTREGNDLYHEMMDSKVISVKEGESRCALIFGQMNEPPGARARVGLTGLTVAEYFRDEEGKDVLLFVDNIFRFTQACSEVSALLGRIPSAVGYQPTLATDLGALQERITTTQKGSITSVQAIYVPADDLTDPAPATTFAHLDATTVLNRGLTELGIYPAVDPLDSTSRMLDPITIGEEHYTVARGVQKLLQDYKSLQDIIAILGVDDLSEEDKLVVARARKVQKFLSQPFFMSEVFSGIPGRFVNLKQNIASFKALLEGAGDEYPESCFYMKGDLEESLAAGRADALKSK

>gi:118355000 ATP synthase F_1_ gamma subunit (Atp3) (4 peptides)

MFGLASKGFINTSLVMVPQMNFGANLKQLKIRMKAIGSIKKITKAMKMVAASKMKAETSRLENGRNFAVGSVQKMLENESYVQKKKSTTAPKSTLLVPITSDKGLCGSVNSSIVREVKRLALNNRSAFGLLPVGEKGSSGLSRPFPDLLKSSIVNIQNVNFPTAAAIAHQVSTQGAGYDQVTLIYNHFKNAISYVVKHQELLPRAQFLNLFKYVTRHEAVEPELEYSKNYFFELYMASSVYNALLNSSASEQASRMNAMENASKNAGEILSKLTLDYNKARQAKITMELIEIISGASIV

>gi:146182760 ATP synthase F_1_ delta subunit (OSCP) (3 peptides)

MQVIRKHLAQKGLFNPFLFNRFSTQQDITSVPGDKPPAIEDSIHGKYAGVLFSSASSNKSLNKVAEDMKYFNQLYKESEVFKSFLNNVSLKRNQQRDIISALGKTNFNPATNNLLETLIENKRLDSLPKIAEKYMDYYRILNKQESITIISAQELTAAEKQKVEQGLKKGNANVQFTVVYQVDPAILGGLQMYSGNNFLDCSLLSRVNKLKTEIAKISF

>gi:118355322[revised] Putative F_1_ delta subunit (3 peptides)

MFTRFVTQPTLLTQTQRALFSALTKKQKMEVTLRTPYKEYLANFDGFSRITAKTNEASLVIQNKTPASLYVLPPGPLKIRFTSEVKNVSGDFLHTGGWVIVHADNTCEINVMDLFDRKEVRADQFEKGNIQDLDTLAGKYAAKSRKSTVRLFTKATTQ

>gi:15027631 Ymf66 (product of mitochondrial gene TepyoMp02; subunit a-like protein, putative) (2 peptides)

MGRENVLPVHNDVYEDFVFTTPYFQPESTFKSVPKLFSDILLGGVEWVYTTSESVLAYDYKLWYLWSGVSNLDESFDMFFNQYWALSLSTSVFQLFYAVILDRYLSVLFQNTPYTNDWFRMMLHSKETALIWLYHPELSWHINGLNQFFTYFYGGILEFVYFDKSNPDMCILVHTLWIHLLILFLIFTGFVTILFSFYGNPNTEENTIDSDYLAASGTVEAEKEITSIDDYLGLVFAIAYVFGVFFYVHGWTSMLSHAVLLLSCYSIIIMFLFILGMPTLLLYDFGIFFLAYLKGAGKYISSVAEMMFDYTACLVFYIRILAQWIRVVLMVVTFISLSHYVSDFDITNSALIGSENQSDSMNELNTNFSMTYYILTVLPGKFIYWIYEILHTFFVVCSQFVAFFAIVFWLFLFLYTFFIIEKHEDFFSKKREERKKKLKELWNLKN

>gi:15027647 ATP synthase F_O_ subunit 9 (subunit c; product of mitochondrial gene TepyoMp18) (3 peptides)

MLLVKAVKVLVMGGCMLPIAFGALGTGVLFAGFNVALSRNPEETESLFNNTLMGFALIETFIFMSIGLGFFVLFAA

>gi:118360532[revised] Putative F_O_ d subunit (3 peptides)

MSMLAKIAKNVVKTQALKNTTAAQTPSFQAPGNQDKILKWISTLSNKATTGESRSYCTQLSSLVSFYNKQHVEQIPTIDFNEWKSVISTQGLVDKVKENYESLIKEQYNTDAISKQISSASSKALDDIENELSFHAAIWLNAYADYTMFLFELEEYNDPNDYLMHENFDFFRGLETELEELTETHNYIPGAKDDVNLRGYLATQFAWGKKVISFYRHPADDFKCAKATKNMLGR

>gi:118384478 Branched-chain amino acid aminotransferase family protein (2 peptides)

MHLLKSFVPKNCSQAMSISNLAKRAFSTSYYANMDLSSKQTFYRKDLTIELTKNPIPMPEFDAEKLRFGAHGTDHMLSVDFDSKKGGWGKPYIHPFRNLEMHPFNSAIHYAMQCFEGAKAFKGTDGKIRMFRVRNNMYRLKNSCKSLALPDFDGEELYKCIEEYVKIDEKWIPPIRGFSLYIRPTVIAWEDKLGVRPADKAKLFVVFSPVGPYYPSGFKPIKLFTETKRIRAAPGGTGNMKVGGNYAPTIPISQNAEQKGFQQVLWLWQDSILEVGTSNIFFFWVNEQGEKELVTPYLDEGTILPGVMRDSILSLTRKWKEFKVTERRITIDEVVKAAKEGRLIEAFGAGTAVIVCPIEEFNYQGVTYKVPINPKKQAGDLTYKISETIQNIQYGDTPHEWAPIVN

>gi:118397639 Lipid A-disaccharide synthase (14 peptides)

MLTHISRRYFSFTGRKTIFVAAGSPSHDLQAANFMRDLKKKSNNNYDFVGIGGPLMQAEGLNQSYADINKFIDKPFFPLKNFIRFHVARCYHPYMAPLHFFNKQVLNQVDKSSLLKDQVELSIPSAIITFGNEFFMKKLYVRLCDQYELHNKIRPPTFFYDRSHINQRFEFQDYLDHFFYTIPMKQINFQSFTYPSTCVGHEGVGRAIQYLFQNSKQYANVKSLVTANGLKIASNPKQHREIIEKLVEEQRGIQRARLGINESKNVFLLAPGNTKAEINFAVNLLSRSLEEFFKKPQLTNVSRDHFTIIITADNAQNAEFVNQAVSNTKYLKTLQTIVTTGEKEKFGAMCAADVGIPLNGELVSECAALQLPSVIISNMNLFYAYITQLYNNFYSDINFAIQGEAYHELVSTAANPYKLSDEIFDLYSDPKLRYHFAERYQNVVHEMIPQANSQDNIVTTDVATLHGVEVQERAFTYETIAAKVLKAARAYESLDKNIPNHQIDQHRKEKLIKAAF

>gi:118384179 Peptidase M16 inactive domain containing protein (27 peptides)

MLSKITQNTLKSLSGQCKQAFSVRKAFYDFIYKDDKSAETYKVTTADPRTPVQGFRGQTAEDVAAKYEVTKLANGVTIITESQTFPSQVDMGILLDVGTRDETNETSGSLLSIKNTYLKTVLNTNETINYGVVQQSGGSFEMEYDQETAYFKANCLAHDATDVFSMVADCALEPRSTVAASVGVEKNQNTHKLESYLKTGELFNESVFKTAYGLKGLGLPLKGLRGNVKNLSSYTLQKFQLENITPNRIFVCAAGVESHQEFVDLVQTKLAQIPSAEGQKTHQREKSEYLGGEVRNLTEESNVTLALLFQSVPWSSADIVAFNVAAALLNNLRLKKNLLQKYAYFDQAEALNFHFTDSGLFGLRTSGSADRAKDILNHSIAELKAIASGVNADELLTAKAALKNSVLSALERQTDRLEETVKNVRTFNKIQHTDYVKQIDSVTADQVAKAVAKVLTSNPTFVAQGSQVNALPTYDAIRNLLK

>gi:146175330[revised] Conserved hypothetical protein TTHERM_00137990 (with **s**imilarity to COG1252, Ndh, NADH dehydrogenase, FAD-containing subunit (E=4e-10) and COG0446, HcaD, Uncharacterized NAD(FAD)-dependent dehydrogenases (E=6e-9) (8 peptides)

MIHCLRNIRTVSALQSKISYNLGGGNKRKKTSGDLDNYDVLFVGANLGGICSNHFDKDTHGKYKCFVSFDQPINQIYSVRIPYEQQRVRKSEYIHFSKKSINQFTPSEMLAVKEILPEQNAVVLSSGRRIGYNQLVLATGLKHDFSQIKGFYEALEHPEHPVYANRDPETWRSAQHKYSKYISNFKSGDGYFCIPEYPYAGEVECFNFFVSDEVWKWAQHHGALSPKHTFTIVNANEKFVHYCDSADAFIKERLEKRGIRVEYNTKLLEVHQDGQKATFINTKTGEKSVRDYNNLYSIVPSKRQEFLDKAGLTNGNGLLNVDHQTLQHKKYKNIFGLGDAADLPTTKTFWAGWYQIAVVRNNVKRNLQGQTLNAHYDGFSKVPLFTGHQTLTYVAHSYGGVGNWQHLKHNNGGILAWMRYRSWAKGMAKKFQDFYNGARLGPPYHKVLKSFPELPGSPESQQSSGISKYFPTKTENKAAH

>gi:146163301 Conserved hypothetical protein TTHERM_00145440 (with **s**imilarity to COG0596, MhpC, Predicted hydrolases or acyltransferases (alpha/beta hydrolase superfamily) [General function prediction only] (E=2e-07) and pfam00561, Abhydrolase_1, alpha/beta hydrolase fold (E=1e-04) (4 peptides)

MINTRKQVFKCLWGAQPAYNFSRIINHLRGDPVYQDNTKDVSLRGTFLRGKYDDLPTMIFFTEACDLTANWIPFFTNPQYDILAHRNVWLLNPRNFGNSDRHPSFDLQEMSDDVMRFMYSQKISMATLGGHGIGGKIALAVGCYHAERVTGVFSIDSSPMDQRYHEAFKEFKGYVNALTEINFKTWSDKDVKVFLKENIKDPKWRSIFTNNISKNAKTQSDSFNFEINYLNHNLNFNKADSLGNWAVKNGIYTGRAHFIFPEYSRWVHLATNTLPMHKVCARVKGFGHDIFYVQGDENPLNHWVYDFENYANVVASKLNKFLHSYDGVHALLKDRTEIGNFMIPDRIKSRNDSKHIYGDYSPAHLHHNWRFNHIYEKHDELDKKLNEQ

>gi:146185889 Hypothetical protein TTHERM_00529760 (3 peptides)

MNPIQKAWLKILEPVSYVINEKMAKRTGIIGKLGRFFAIGPREYGVHPINRMFIFMNRKYMAFQAVALHRYSFVKSLTHNGFHMLRVFRHFAFVLPATVLAGLGLFVYWGDDNKCYSPDRFPYLKKRAGDMALPLNSLNQRTSAHYIEINAIYGAEMMKRYHKVWENIIEERSKATDQEKKTRYAHPSYQYSPLPVVSIPNVLNPLNLQ

>gi:229594811 Hypothetical protein TTHERM_00486310 (2 peptides)

MGYKIRNKSIFWTRAGWKNNWHPKNFNAPRPSYGEFTMGMRCRNDHHSFLRYVQTYRNMSRHCKQYFLGDKQLEETFILGLRSLFLVPYDSQCLTDQIKHGGERRFVDQLDRDFELISYNTHPYQLFTYTVRNEHLAWKNEQYEKIQKGEKTFEQELLDYLDEQVLAEKAKLRDGQNFSIERMTEIALHVFRKARAGKVRPAQDVRGPDGNVNDFLEQRRPFEHPNPTGVTH

>gi:146161614[revised] Hypothetical protein TTHERM_00068120 (7 peptides)

MSQDPKIVNPQLWPNPNKLRFADLYKYQGVEMKKINDSIKNYKAAKFYIGGILGGCLVFKFFIDAAVDKYIFGENGNGGKFLEMQTINSNYDYYYNRQFQRMRYLTEDPAGDDPLQKTKDEHLVDLGFIPKVFGANVEVRKRAPHDKYL

>gi:118399953[revised] Hypothetical protein TTHERM_00649060 (7 peptides)

MPVKEGQAKLWFSTKEEADAYDDKMISNIELKSQDYEDENFSPVFNRKTQEYFLEPSEKFKSDFAELLRPLRSLSFNQVVDRYVLIPPNHTFYRNWTYEKFLGGFGLSYLILRELPLRNFYARVFVMYAFAAKVLDHLGNPFPFSGHGQIVAAADRWNHWDVRCYDNVMKALKYIRIPTVQNNIPEATRWYGRQPGHLLRADTYWIPNLVSQRFAKHQPAHWDGTQNMPIFRLADPKHKDSYMVQFR

>gi:118366175[revised] Hypothetical protein TTHERM_00127260 (7 peptides)

MHSTLRVFTKNNCLSFTNMNRFSTAAQVAQANYSKFRADYSASVAAFQQRIKTIEKENTGSMKKPMAKAYEHPYNSEHHPLNFSAVKIAETFHDFIGPEQVSPHYESFAMSRKFLLTFWGGFFVLNFGMATVDLNWIMKSTYIPWIFWFQLMYFYVEGKNSMFMPLLQRFYRRAAANEIFTMEAFYHENIENKLRNLMRITKGQLEYWDIHTSYGEIRADSINNFLANEYLRLQSHITSRALNILKQAQAYETMNQAALLQKLIDDATSAIDNALKGDKKAEVLARSLDSAIDGLSKGYMDYQNDPLLPLILSSIEANVKKITTLSAQEQANLIGLTAEQLKSIKENDVRARKEFLESQPKLDNNLKNIESVKKILATWGK

>gi|118370910 Hypothetical protein TTHERM_00289380 (3 peptides)

MTIFAETWEASEYQYRNKANLKTLPVNHLGKLAELKFDFVEYKAHQLIACHLYERMTIHCMNQYGLFKDFYRPECLDAQYYFKTCVELNAAYGIQKKFFPEHFVGSPYARPVPQFQQLGL

>gi:229594147 Hypothetical protein TTHERM_01528510 (10 peptides)

MSENKAPGQIYAYDIHNTHYPYVNIKQDSQTQLLASFRRSIASINPFSYRQVPSQDRAAFGLRWGNAWYAPNPYPNGIHFDRVFPTHYDPLAETNRTKANLQLIKYAPGNYSTLVVTSEKLPRPCIRTIQNYRRCQMVNGTEKCNSEAQDILAICPNWALDHMKEKVRFYTKALAINNQTYIRAMQVEEYNQGRTVADVAPKTWIHGTRQHLRPDTMWADDRYTNITQTEINEAIKRVEARKAREHEKKPVEQANVNANTGEQPVRVEKSLYP

>gi:146180703[revised] Hypothetical protein TTHERM_00316290 (2 peptides)

MDNYFTAITLLGLRDQNLPPFKDARLQRYKSIKKMIDLIETTTKLAPPMPVELFMLNPTDPEWDDDMTYPTITHATALYKSSALAGNLFLYAYNYNNFTANIRLRTMRYLFPVVSLAIFGNIYWDYRSQLVKVNLFDEYIQARAQELVKQNEYLLEHEDVKRYVWWYEDLKETLARVHRQANNHKACDFKDSEIILQDFIRRYTNPKDNLPIKFHPQGQTF

>gi:118398278 Hypothetical protein TTHERM_00823660 (3 peptides)

MWYKYFSKQSWNLRVWRKANLKYNQDDFGMTQPKYIARFGDFRFRLVRTEGALRGCMFFVGFGCFSIINYLYGRYGYIINESSQKRAAQDLLDNDMAADKILFKNRVGAPTRPLRSLDDMMAFLSGSATYDQLADYASYNHAMDVNQDQQAGLDSWMSEKDKNMVKYYQRSLGKKVEGI

>gi:118398135[revised] Hypothetical protein TTHERM_00825290 (3 peptides)

MSNIFLELQDGDKTVYTHTSLIEESKQEQIQAIYDKVPQWTNGGRFLGFWLSMEAVNRVQSVAKLPIYYRAGIVATSTLLGGLVSSLVFWKSGNENQVAKLANGAPVYLKKWEVPELSKLYFFLDDDNNFKPSLNHHAVTQGRQYYKIYQHN

>gi:146184052 Hypothetical proteins TTHERM_00571670 (2 peptides)

MSLHEKMQTDYLWVKDHSQADSWAKARTHGYNYIAHTVPNKKERYEMIWRSMGKSTDWELEKFRLGKKFPDRGNKRRWFKNLFRLIKNPMGYIFWKTYKARLAKPSLIVTSMFIGFTLGFIKLKAQSIAYSKKQYATLRAGKNIEGSGQVHFGYHDQKWGMPAIPMFQLMYYELPGNSIVVNPCRNQNYRLYFEMRKKLGILPA

>gi:118397435 DnaJ domain containing protein (8 peptides)

MIVNRLFRQQFRNISYGFATQKLDYYAVLGVDRLATAEQIKDSYRKLAMKYHPDVNTTVTEAHEPSARKFQEIAEAYAVLSVEEQRRAYDFLNQPSPYDRYTEDSALEDELLRRRSVDGNAIRQPHKVGTYAAEKQRLLAEERAKFNVDHLGRYKGGLPVKGKGSIRKGIHGEGFGAPSHAHDALIHQIKQSKDTMDYQNITNEVAQNFANHQNNDRWVYERRKSNFIAQVDYEYFKFNHWRTAWRYFRNIFLLTAGVSFLYNMELDEGLGGLSLKYKEFVKTNPGQDLLIGNIRVTQRPNGLLVAVDAHQKQHHHH

>gi:118378329[revised] Hypothetical protein TTHERM_00557760 (with similarity to COG0702, Predicted nucleoside-diphosphate-sugar epimerases [Cell envelope biogenesis, outer membrane / Carbohydrate transport and metabolism], E=3e-15; including putative ortholog of subunit Ndufa9 of complex I in plants.) (7 peptides)

MIGKTQQRLNKCLIQVIQKQFSQQRSTQLKFYDGGNRQSISGIRATIFGATGFMGPYIGAALGYIGSDVIFPHNHVYAYDDYVKELKLCAGSGQSYIMRHFNYDDDNMYDMAIKNSNVVINLVGSRLQNKNFQKAAYANIHVAKKIAEACARNPNVRRLIHFSAAGADTKSPSPDLHTKFHGEEAVLNAFPNATIFRPCTVYGMQDYFIRHWIKERDWWYHFNIVTDDCTAKRQPILINDVAQCVLNALKLQESAGQIYELGGPHVYSRLEVFEMLANLSGRPPKLAHIPHDIALKITQNFYNWEFFNMEKVIKDKLDLIVTGKHKTISDLYVQPVSFPQGAEQFIDDVRYRGVETHDNLEK

>gi:229593554 Hypothetical protein TTHERM_00526930 (with similarity to pfam04800, ETC_C1_NDUFA4, ETC complex I subunit conserved region, E=4e-12) (4 peptides)

MLLPKNAIQSARYFEIQKIPAKIAKTANEKILSVGVAQKNNIQPKTVTAQGQIGFVQHPQLDSSCQYTQFYTPQRRDIRGRVARIYIQDTNHMHDTPQIPEGYKWTLEFERQAQYKTPWMGWSFNGDTFSKRNHYFCTLEDAISYCKQMGFGYEVSFPRSRYHTRKSYADNMLWPGHDNAVDEDC

>gi:229595475 NADH-ubiquinone oxidoreductase 75 kDa subunit, mitochondrial precursor, putative (18 peptides)

MMKVLQKYGKQFYGLTHIQKKQFRSTQVLNKQIEIFIDGKPAKVDDSYTIFQACYENGVIVPRFCYHERLSVAGNCRMCLVEVENVPKPVAACASQVVPGMKIKTKSEKTRIHRGNVMEFLLANHPLDCPICDQGGECDLQDISSVYGYGISRYNEYKRAVEDKNYGPLVATSMNRCIHCTRCVRFATQIAGVEDLGKTGRGKAAEIGTYVEKTFNTELSGNVVDVCPVGALTNAPYAFTSRPWELKSFYTSDVFDTLGSAIQVDTRGPEIMRVLPRIHEEINEEWISDKTRHAFDGLKRQRINSPMKRSKDGNYEDIFWEEAIQTISKKCLNTPSDQIGAIIGEFADIESITALKDFLNRLDVDNFEVRQHGNLKVSPDFRANYLMNSKITGVEDADVLLLVGCNPRYEAPVLNARILKSTRKNLKVFNIGTNQDLNYKNVHLGNSTKVLKEIADGTHPFAERLKKAKLPMIMVGASALEREDGAELYNTLKVISNKTGVISEEKSWNGFNILHKEMGRINALELGINPTSVNKNAKLVFILGADNNLRPEDIPADAFVVYFGTHGDEGAYYADIILPTAAYTEKNATWVNTEGRVQQGRLVVMPPGDAREDWQIIRALSEEAGVPLPYDSLEELRYRVAELAPHLLKYDYIEPTIFGKVALSAQQGVKTTLSPTPITDYIDNFYMTDAISRASVTMAKCSTAFNHEKFSNFKNLAK

>gi:146181190 Peptidase M16 inactive domain (22 peptides)

MFGRISQRISKLARLQRAFSTLQKQAQSNVVKSERLQFSKARLTDFGELPQGEIPTALQYDRPCRVETLANGVRLAVEPSSVSPLAAVSVVVRAGTRQETLETSGVAQFVQRLVLRGTSKRNREQIEKELALLGGNLKVQVGRETTTYTLSVLPENVEKAVDFLGDILQNSVFNKQQVEAEKEAVYNNALSAQNDQQGLLLENIHFTAYRDHYFGQPTHGIRENLHNITDEVVKNFVKTNYVGSNFVVAAAGNVNSQAFLQAAEKAFGTVAQKDATTFVPNTEKPYFTPSYMTIRDDEMHNLNVGVFFEAPSWTDPDFFTINFFQRILGEYQADKYTGQHLNTSDRQYSLIHKELGNLPDVTIHKTHYLPYSDTGLFGSYFYGNEIFGNQMLFLSQMILSEYASYINQAEIYRARAKYFNELLAEQNSADIASSIATQVTYLNRRVPRSEVAKRISSLDSGLINRAATRWFWDKELAIVTWGPSHGLIAGSHYNRSIKRSTLGWYGNTHYYIV

>gi:146164447 Rieske iron-sulfur protein, Ubiquinol-cytochrome c reductase iron-sulfur subunit (15 peptides)

MFSKTLAHVTRSCNKLNQVQAYNFGVLSEYNQRLSKKLHKGHLVEDKPTFFVTSSRPGNFGDHIDFKVNIDNWFDENRVHNEHETDIRRTQIYTLNAIYYGGLLSFARLYAMGVIGRLNGWKRYERDTYSEVDIGALPPGEVMQMVWNGTPIFIRRLTSNEVKEENELPSNTLLDKDKEVILSDAGNTKVIVVSAVCTHLGCIPIPYLGAYKGYVCICHGSVYDKFARVRQGPALLNLPAINNSIHDEGTLVCMEQLKFPHEPSQRFWA

>gi:118389517[revised] Cytochrome c_1_ family protein (14 peptides)

MKSFVAAGIIGLSLANSQNKEVQNFIYRDDIGAFWGIKGYEELVTEVGTHKGHNYWPQFSFLGTYDSGSVRRGFQVFARNCGNCHGMIYKKYDYLLDKAYRQLELAQMVSDFTIHPAHQHFKQYYYQEWDERDRVICDHIYPPYFSQDQAKNANGGVWPTDFSKIKLRPGGINYIYNISTGYHFTPPFGMDVPKGKYFNPYFDHMIIGMPRQLVDGLVDYDDGTPASTPQMAYDVSNFINFMQRRVGYKRPDKMVRYYMVFTGGLLILPFKYFKTKAYYRNLLSLRWEMYAVRDGVYYNHFKYGGYNSRAYQFRGYFWA

>gi:118373062[revised] Hypothetical protein TTHERM_00136440 (with similarity to cd04645, LbH_gamma_CA_like, Gamma carbonic anhydrase-like family, E=4e-48; possible subunit of complex I.) (8 peptides)

MLRLRLFDAYEKISMTFLGPLYRRIGKSLAQTGLNIQQPYTSDDRLVPSLRNIRVTNKIPSINDSEFIAPNSVVIGDVITKEGSSIWYGATLRGELGPIEIGKQTVIQDLVNIQSGKQNQKTQIGDNVFIGPNSYIQSSKINDNSFVGMGSTVSTGCNLASNAVVAAGSVVPENTQVPSNQIWAGSPAQYLRDITPEERQVLQEHHQECVQLARIHAEETEKSFREVLNDFDRITAEAEYDHESLALQKMRDLGFPMEGEEEEYIEQRVFMREQLPPLESEFWKKNYDPYEQDLFHFPDSFKAYQQQYKRYDEAKKYFEENPNVEATIIDREFKEPTNKKPWTRKY

>gi:118382425[revised] Hypothetical protein TTHERM_00532490 (2 peptides)

MYHIKTYFESLLNLKERRSNKYLLKQRIQNEKKFCKQNQREKAKSKELVKNKSKLSKYKMNISYTGLKLEDYSDEVIRKYKFPNSNELERFLNREQTLTVQQHKSAIKLAQQDFFAVAGLLSVGSLSYIFYNSVGGKVIRDRIRASMPFPKRVLVQVLPFVALGTALIISRRGIEGHNHGYKQ

>gi:118385011 Hypothetical protein TTHERM_00697370 (4 peptides)

MIARRLFKRSLYYIPRAGFGGGDIRHKFSNEITDDDYDYQRAMHVKPPKEESLFQLTNILSSVPVFKTRFFLDFIARNLDTNSAVSTSDFVAPPRVHENSFFVYHSRELGNVIRKYRSLESIVLPGALLTFTYPLFAAFVAIPSYYFMFNAKIYEMSRRFVVRMDVLPHLEMISVQRIGAFGILYTKLHRIQDLEYVPFDQVKEQENYLWAIGGHGVDNQLIFKDRSTGEFFYFERQGVWDAKGLNHPLLN

>gi:118388745 Hypothetical protein TTHERM_01000190 (with similarity to PRK08315, acyl-CoA synthetase (AMP-binding domain), E=3e-20) (10 peptides)

MKLFSQVNRFAFSTANKAASPLAAQLAINGNRNAVRYENQNRTWTFNELDAHTNAFAYGLTELGWKAGDKLLLWVEKNHTSEITTAQVGAAKAGVTLVPIYAHSAEELEKALNDTKAKGLLLSPNSKAGNSKYIEVVNKVIPELYNTGRGSTLKTKFANLQHIIHTGFYTFPGTYKFRQIMVYASKNFNTLTLPNVELNAPLFISGNQTYTLKDLISKTEENRKTSKLNDNTPVFVTGDSRSPLSFSLGILNSLLHGNYSVYTGAQDLNEVGQTIRFYDNALLLVDGDIVKATQSLKHSENFAKLGGVAANENIPKDSLNQQFQIYQIIQQLFKQYNINQKIIWWQISLIAQNLKDTSLIIIKMFFVKMIFDLKQNLKKSPILKI

>gi:146161176 Hypothetical protein TTHERM_00541460 (with similarity to cd04645, LbH_gamma_CA_like, Gamma carbonic anhydrase-like family, E=4e-43; possible subunit of complex I.) (5 peptides)

MKLFQAMWTRTIYSIGRMVRETGLGMLLFSLTLDRYGCKLEQDISCYEPLSRHRNILPIYDLVPTFYHSTFIAPNSSLIGAVYLGQNTVVGYGSTLRGDNHAIRVGHNTVIGDKVAISNVATLAAGIPVSTNIGNHVNIGAGCVLQSCVVDDNVTVGHNTVILEGSVLERGSVIAPNSLVPAGRLIPSGQLWAGSPVRYVRDLKEEEIKLNLEQTEQNLSIGKTHKSSLIQQEAYDRLLA

>gi:146164764 Hypothetical protein TTHERM_00399360 (9 peptides)

MRKALERFNEIIFNPAIRWYQLPKPTVRRTRYPAPGSEPINREVHQIDYKTAFRDSPHNIRYHHEIHTSDQTYHSSYDPVGETTTERLVRYGYLNKDQVNNAEAVAAAAKEFQEKEKRSPSNNIIIDEISNSDKPITKENRESVAHHVRQQFEFFREVNAEEVWSVSIEEKYNPELYIYKTYDMAADDPVWRQVKLDLEWTFENIAERRESLGYMPTFKGDPNFWQALDNSFSPENIAQVQSSIGDKVTNIDTKALALNHQTEEYHKTSKLVYPIRTNLVVE

>gi:146165634 Hypothetical protein TTHERM_00382330 (24 peptides)

MVRLEKILWEQLVNVKAFSRQRVIGAPSKWYNENRTEWFKVAQHNAFNTGFSGVILRALEPLLAKFIYRWRLDIAHQRGLTLEDSLLFMDRELRRCYFFETVARQNLHPYTVLFMKKRRARYYKVERGLRGFYVPDWVRKEAEERQLSETVDNIFNWENFVYREYMSDMTPIGRWTSLSKITPLDMFQYYGLFRNEAWDRFFYNEAFYESYSEKEKQEANGNPFGKFNLQTADGRAQFEKEVNTFIERYPFAVTKPGQKFDFTRFYALEDLANKRDTSKYDPALLESVKNELKQSAALPADNGANKTKKSKPILPDWLQPKFGKAFQA

>gi:229595595 Hypothetical protein TTHERM_00649080 (5 peptides)

MQNAYPEITDQHREFLKKQGLKVYEPKPLPDQINPFSKTYWITNAFIIGVSFLARRHALKVGAPRIFWSGCIVGVPLAAIISRGKSDQLDELVGARKTLEQKLEYAPITRRAWERALATNQEYQNEIKTQIQDLQAEIAAKKVAAKLE

>gi:118368475 Hypothetical protein TTHERM_00481330 (2 peptides)

MALRRVLKNQFNLIHKGQAQAVRGGHGWDRPDVPLSFNPLYVHKRELSIFDTNMWMYDQVYPEYVISYNEIHLVDQWKGLKESFSQSAYWWAMMAMVFGFYFINTTPRQLGIDTNDLKGFLGEYYGQYKKRSGIRSNFLGLDVTGENSIIQPNYDRKNGIRDVIDSLNADAGKRKLINLEAKNFIERVEKECEQRILKKGGATQSHH

>gi:118354116[revised] Hypothetical protein TTHERM_01005010 (with similarity to cd04645, LbH_gamma_CA_like, Gamma carbonic anhydrase-like family, E=8e-54; possible subunit of complex I.) (5 peptides)

MKLFRALTKSGLIQKIRQATGSEISTATKYGENISKHRSLMSLYDLHPQIGYQSYIAPNSTVIGEVTIGNETTVWYNSVIRGDVNAVQIGNNVSIGENVVIHTAGSLPTGQPASVDIGHYVIIGSKSTIYSCTIQDEVVIGQGCVILEGARIEKGAMIAANSVVPPGRLIPAGTLWAGNPCTFVRNLTKSELATNIDHAKKQLHLAQQHRYEYLPYNSAYLQKSNSEEDLNPTKYDDVTINYNFGDEERAQENPLKY

>gi:118370180 Hypothetical protein TTHERM_01161000 (with similarity to pfam04716, ETC_C1_NDUFA5, ETC complex I subunit conserved region) (4 peptides)

MLQRGLAKFLLRNTRQTFGVVRNGFSSTQIEKAAEKSDEVPIHLRPYNKEKYEVPSTKLKYSTGYALLDVEPMPRAKIMKISYNILHKLKEIPETAMYRIYTEEKVKYIMKLTDEIEDVRQLEEEFGQDSIELFIYSYGKELELVEYMKHSKPWESRPGDEEQNEMIRIASQKRVYLKHQRVDRPARQEAAFISSNAKPQLEAGKQ

>gi:15027638 NADH dehydrogenase subunit 10 (nad10; product of mitochondrial gene TepyoMp09) (2 peptides)

MRVDYIVTPINAVVSWARQGSFWPLTFGLACCAVEMMHATVSRYDFDRFGVIFRATPRQADLILVAGTLTNKMAPGLRRLYEQTLDPKWVLSMGSCANGGGYYHYSYSVVRGCNRIIPVDIYVPGCPPTAEALMFGVLQLQKSLYRQINEGEKKYNKKDLTY

>gi:15027641 NADH dehydrogenase subunit 7 (nad7; product of mitochondrial gene TepyoMp12) (8 peptides)

MNRSIFQLWKPESPRSNVNSKQIKTMNVNFGPQHPAAHGVLRLILQLNGEIAERFDPHIGLLHRGSEKLIEDRPYLQGMPYFDRFDYVSMMVQEHAYCLGIESLLGTTNYSATFTQIRTMYDELTRILNHLLAVACHALDVGSMSSVFWAFEEREKLMEFYERVCGARMHAAFYRPNEVNLNAVSSFLMEDILEFSRNFFTTLNEMHNVLTYNKIWKQRLINIGTYSFQTCLDYGLTGVMARSCGLKRDLRLSKTETYANYYYLNFRSYTGQHGDCYDRFLIRMNEMCESLNIVNQSINKISKFNNIVSINTKKNILNKENFNRQTTVLPHLVLSYLNKNDYNLKNTKNDYNSMEELITHFKYWSKGLKVESGYTYQSVESPKGEFGVSMLSDGSNKPYKCKVRSPALHHLQVLPKIGKGHFLADLVALVGTVDIVFGEIDR

>gi:15027658 and gi:15027659 (duplicate genes) NADH dehydrogenase subunit 9 (nad9; product of mitochondrial genes TepyoMp29 & Mp30) (4 peptides)

MQNKYMQPIQLFIVFEKLNSKMWISKKLNSNHSIALIPSNWFYSINLFLKRELLFSNITLIENSAIDTISYNLETNQNDLDKTNIENYFFKNKMLVFYNYYNYFLKSKLTLFIILNNLNKSIDSIDRIFANANWLERETSEMYGINYKWKIDTRKLLLDYSKIENPMLKEFQSEGTQDAFYNIFENQVVVLKNETVEL

>gi:118367869 NADH-quinone oxidoreductase, F subunit family protein (NUBM/NDUFV1/NuoF) (5 peptides)

MLSKIFKTTVIANKKFLNSKNLSMVISYNRSYGNLKDQDRIFTNLYRDGDPFVKGALKRGDWHQTKEILSNGPEWIIDEIKKSGLRGRGGAGFLSGLKYSFMPKVNPDGRPSYLVINSDESEPGTCKDREILRNDPHKLVEGALVVGFSMRARAAYIYIRGEFWVEANILQQAIDEAYAKGFIGKNACGSGYDFDVYIHRGAGAYICGEETGLIESIEGKAGQPRVKPPFPANAGLYGCPTTVTNVETVAVCPTIMRRGASWFASFGRPNNAGTKLYCISGHVNNPCTVEEEMSIPLRELLEKHCGGVRGGWDNLLAVIPGGSSVPMMPKNVCDDVLMDFDALKAVGSGLGTAAVIVMDKSTDPIDAILRLSKFYKHESCGQCTPCREGTGWIVDVMERLLVGNADYAEIDMLQQVTQQIEMHTICALGDAAAWPVQGLIKNFREEIEDRIDSYHAKHPQLKKSRKSNPQIGHH

>gi:118400152[revised] Hypothetical protein TTHERM_00637380 (5 peptides)

MLKRIITNIVKLNGQRPCLLMNNLKGSNCLVQNVAFNFSQRGRDYTPSNKKYLQPWELERKEYVELSLAIQSAYSCKMLSEILKDNLYMLTDYQLSFAMFHLWNHEIPIDNYFYNVISPILKEYITRFDRECNKSLAEIATFLGRMNVQDDALWKVIETKLVQERLYRYIPLNDLIDLAHGMATANRGSQEFYNIVENVIIKHRLRLIPDKIAVAKDCFTARKIGSPLLYQVLENPQAEAHELAGLKEHEQLKISG

>gi:146161872 Hypothetical protein TTHERM_00006120 (3 peptides)

MTHQFENVLLSNRKNLTPQESVQKVINYALLQDAKQRSRTLRHIKASWVIPALLFTYPAWYLAKGAVNGVWSNIHPTDKVTLSFANIGRPFRLIYRPEIFLRDQQAKFIQLEKEHIEKSKKGEFVETTSPLVLWN

>gi:229594864 Hypothetical protein TTHERM_00148710 (2 peptides)

MSNNNQGDFFVDKYNFSRRVVDHRQPYDLNFSINNPVGSRVWFKAWKQKAIGNFLNLVGVHYAFYGAGFCLLFVLADAWGREKYAQPYKSQILHGRQPFGHTFVQNYRNQATDLGRWNHNFACYEKQPGCGRDFD

>gi:118365429[revised] Hypothetical protein TTHERM_00268000 (2 peptides)

MSGLLRNFEKLVCQSQLSKAGHKLLLRSPNSTLHPTAFYYKRNSSQRLANEMDVFQLGLAAAALTRQANNYAQLLDQVDKEAVREEVQERITQNHSDLNVYFGEILSLFKIGKKECPVQTVADISYVLAFGPIQVPNAAAIITENLLPVLKEKLDYASIHNLQDILSAFVKLNYVSDKELLKRLITALSQKDFPNQLQPVTNHAWNIDQYEYSDCNSWNIVSCGDNTFEKYIHEGGCENSLAKAKFAVHELLDHISFNFVNPFLFRENRINHRFAKRNADLDHEVLMQTLSKLQEIVPETSEAIATIKARL

>gi:118401281 Hypothetical protein TTHERM_00470710 (with similarity to TIGR00517, acyl_carrier, acyl carrier protein, E=4e-07) (2 peptides)

MFGLTKNLLKFAQQANKMNSILNKTNTLQWASVYNYAPQKVVLRNEKSGYYANPDDVARRLIRLISLHDKVQNPSAITLKSTWSEIGVDPLSYVEIMLEAENEFYIELADEDLERFRTVEDAVEFIARNFFTN

>gi:146169365 Hypothetical protein TTHERM_00193750 (3 peptides)

MAFGGFRQTDNSLIIDDRRKIILNTRSLNDFQQKIYLRNFFTNYRPDLSSYDYFAFKEKLRIGELFLNEYRKRINNEVRRAAILTPTSSLREKMNHKIADQILDLSSPHVRGAHFQAVRSWTDASKIVNYVEEKQTKINKYGLQFPLLGNMTEEQCASKEDEVYQRLLKEMQKPPKKASEPVEESSDE

>gi:146170370 Hypothetical protein TTHERM_00334340 (2 peptides)

MEFSNALLSQTKLPGQQYDYKRCFQASNTHFECLDSIRDAYDNHHKCPQTYQAWQSSCDVGIRRYQMDNREQQKKDQLLYSKEQLKNYNAAKNKEAYGITAPKNLSFAF

>gi:146162619 Hypothetical protein TTHERM_00160690 (with similarity to pfam10247, Mit_gmP, Putative mitochondrial genome maintenance protein, E=0.002) (2 peptides)

MSSMLIWGACFGLFTRAAACKASMIPLTTSPWKYPKYMIVSAVTFYYFDWYRRMALEQLCYNEEKLERYQIRAKLQSLKIGEELSDAYRESFFEHAVQKNNI

>gi:118381896[revised] Mitochondrial ribosomal protein L51 / S25 / CI-B8 domain containing protein (3 peptides)

MSSWQKLIGTNLRELRFVFCQNSQRSLGLRNYIAKNYWNLKNQNPNFPLIVRECEEADPYIIARYQYGVEKKAFAANNNEAEIENIIKTLVEQSNKVNNQMQK

>gi:15027669 NADH dehydrogenase subunit 1 (nad1_a; product of mitochondrial gene TepyoMp40) (3 peptides)

MGFWIAPLFVNILSLPLYLVMLVYNIVCMLLITLVIASITLIERKVLSLVQRRVGPHYVGYRGRLQYIADALKLFIKGIVVPEGSNKFWFVAIPSAAGAICYTFWINSMWGPSVSIFDLEYNLVYATILSILFSFCIMLTGYFSKSKYAFMASIRCAILMLNIEIFLGLLVINLIFISESFCFSVFVIYQEIIWLIFIFFGVSGLIFITFLLETNRAPFDLAEAESELVTGYSVEYGGFYFALYYLGEYFHLFFFSMVISIVLFGGWELPNFLYLFLLNDFNIL

>gi:146174422 SPFH domain / Band 7 family protein (2 peptides)

MAQKVASAVSLGVAGVAGLIIAQSCIYTVEPGHTALKFSRLTGLSDKQYNEGWHLRVPYFERPIIFNTQTRYKTFPANTANADMQSVNITVRVLFEPIQDKLSELYRYVGQDYDNKILPSIMNEVMRAVVAQYSASQLMSQRDKISQKIQKILEERARVFHINIKNIAITELSFSKEYQEATEAKKIAQQEAERARYYVEMAKDIKKSIIIKAQAQTKSIELVGQAAANDASYIDLKRIEYAKEIASVLADSRNHIMLNSDILQMDASLKTPKRN

>gi:15027661 Cytochrome b (cob; product of mitochondrial gene TepyoMp32) (4 peptides)

MEWNKGTYMSTSIKKIVQYFSVMTVSFHDINSLFGFFTFLTIASQLVSGTMLAFSLVPEPMLIPMVREEEDVEDLYTDDFFWLHERGVDMLFIFSYFHLFRKIYLNNFEYEQEAAWKSGVFTFLLFQVVVFLGLVLCCTHLSDITLAIAANLYDTFFAGKGKFYWWIFTSKELNTDTIIRLAYLHYVLAFFLAYLGLIHGVDMHYDWKNESSMDGLETEMIWFDEALSNELGAMIEIILIVMIVCFFMYPEPEALSYEFFMWGDIGFINDVRFLSVAPHWYFRPFMAWLTVCPFHKIGLFGLIYYFFILFYQPVIHGTNEQNNYTKRNVAFVSFFINRSDIMTPKYHSVEDNLLHQITFWLFLCSALYVTSYLPYGRFYNRINGNYGTLWSFMYIFFYLGNSFLRRPLITELYLFNAFVKSKFLKK

>gi:146182840 Hypothetical protein TTHERM_00765330 (2 peptides)

MNVTGAGLTHVKDFHSDEMRVFRGGLRHIADKQGNLIYGSVNSSVRYYHDKMSYERGFIQHSRSPSNQFINFHFMLGGFRTYVLERFFKQVWYRRNIRTFWFPVLISYTSGCITMRMYDNNCYDYFYFSD

>gi:118382053 Hypothetical protein TTHERM_00456790 (3 peptides)

MVYGKLIFNNIKEYTPSWIKTIPYSQVTKPILRKQPQIVGKINADPKVKKFWVFLRENVQYYPFLWQFFILGTSFVWFHVCYDPWLAIYQANNAHRSLETALTKEKAHKKKLAEQEESE
