# Supplementary material for: Highly Divergent Mitochondrial ATP Synthase Complexes in Tetrahymena thermophila
Source: PLoS Biol. 2010 Jul 13;8(7):e1000418. doi: 10.1371/journal.pbio.1000418 (PMC2903591; doi:10.1371/journal.pbio.1000418)
Supplement: Text S1 — Supplemental results: proposed revision of gi:118355322/trembl:Q22ZH1/Hypothetical protein TTHERM_01094890 and identification as putative T. thermophila ATP synthase delta subunit. (0.79 MB PDF) [file pbio.1000418.s011.pdf]

Text S1. Supplemental Results:

Proposed revision of gi:118355322/tr:Q22ZH1/Hypothetical protein TTHERM_01094890 and Identification as putative *T. thermophila* ATP synthase delta subunit

Index--

Summary of revision
Paramecium ortholog has similarity to ATP synthase delta chain
Apparent extra amino acid sequence in predicted *T. thermophila* protein
Existing gene model and proposed revision
Results of PFam queries
COMPASS searches and statistical significance of similarity
Four *Paramecium* paralogues
Putative *Ichthyophthirius multifiliis* ortholog
Alignments of delta subunit sequences
Tree from phylogenetic analysis of delta subunits

Summary of revision:

>gi|118355322|ref|XP_001010921.1| hypothetical protein TTHERM_01094890 [Tetrahymena thermophila SB210] 228 aa
MFTRFVTQPTLLTQTQRALFSALTKKQKMEVTLRTPYKEYLANFDGFSRITAKTNEASLVIQNKTPASLYVLPPGPLKIRFTSEVKNVSGDFLHTGGWVIVHA
QQFNKRQHKNIHTNIQKFISNLIADLQKDIQQQEKQICNQKKENCEAKVQKFNSQQIKMMNINIIKSTQIDNTCEINVMDLFDRKEVRADQFEKGNIQDLDTL
AGKYAAKSRKSTVRLFTKATTQ

>TTHERM_01094890_revised (putative ATP synthase delta subunit) 158 aa.
MFTRFVTQPTLLTQTQRALFSALTKKQKMEVTLRTPYKEYLANFDGFSRITAKTNEASLVIQNKTPASLYVLPPGPLKIRFTSEVKNVSGDFLHTGGWVIVHADNTCEINVMD
LFDRKEVRADQFEKGNIQDLDTLAGKYAAKSRKSTVRLFTKATTQ

***Paramecium* ortholog reportedly has similarity to ATP synthase delta chain, beta-sandwich domain**

>GSPATP00005023001 Paramecium tetraurelia (156 bp) annotated as hypothetical protein but listed in InterPro as having ATPase, F1 complex, delta/epsilon subunit, N-terminal **domain,** IPR020546. **(note—there are 3 more paralogues in *P.tetraurelia*)**
MNRIANGVIGLALKNQACFSAAKKAKMELTIRTPYRTILDKFEGFSRIVAKTNEAALIIQNRTPAAVYILPPGPLKIKFTQDVKGVTGDFLHLGGYVFVNPDNTCEINLMDVV
DRKEAKVDQFDKSDVKDADTVAGRYAGKIRRSAQRTFIKKATA

>GSPATG00005023001 gene [Paramecium tetraurelia]
ATGAATAGAATCGCCAATGGTGTGATAGGACTTGCCTTGAAAAATTAGGCTTGTTTCAGTGCAGCAAAGAAAGCTAAGATGGAATTGACAATTAGAACTCCATATAGAACAAT
ATTGGACAAATTCGAAGGATTCTCAAGAATAGTGGCTAAAACAAATGAAgtattagtgtattgtaataaattcacaagGCAGCTCTCATCATTCAAAATAGAACTCCTGCTGC
AGTTTACATATTACCTCCAGGCCCTTTGAAAATCAAATTCACTTAAGATGTTAAAGGAGTCACAGGGGACTTTTTGCATTTGGGTGGCTATGTGTTTGTGAATCCgtatttaa
tttaaatctaaattagTGACAATACTTGCGAAATCAATTTGATGGATGTTGTTGATAGAAAAGAGGCAAAAGTAGATTAATTTGACAAGTCTGATGTTAAAGATGCTGATACT
GTTGCTGGAAGATATGCAGGAAAAATTAGAAGATCAGCATAACGTACATTTATTAAAAAAGCAACAGCATGAtattattacataaaataaataaatacatttacaagatgctt
taaaa

2 *Paramecium* cDNAs support complete *P. tetraurelia* putative delta subunit CDS:

>gi|133934818|gb|CT753532.1|CT753532 CT753532 Paramecium tetraurelia vegetative cells at standard temperature (27 degrees Celsius) Paramecium tetraurelia cDNA clone LK0ACA11YM02 5', mRNA sequence
ATTATGAATAGAATCGCCAATGGTGTGATAGGACTTGCCTTGAAAAATTAGGCTTGTTTCAGTGCAGCAAAGAAAGCTAAGATGGAATTGACAATTAGAACTCCATATAGAAC
AATATTGGACAAATTCGAAGGATTCTCAAGAATAGTGGCTAAAACAAATGAAGCAGCTCTCATCATTCAAAATAGAACTCCTGCTGCAGTTTACATATTACCTCCAGGCCCTT
TGAAAATCAAATTCACTTAAGATGTTAAAGGAGTCACAGGGGACTTTTTGCATTTGGGTGGCTATGTGTTTGTGAATCCTGACAATACTTGCGAAATCAATTTGATGGATGTT
GTTGATAGAAAAGAGGCAAAAGTAGATTAATTTGACAAGTCTGATGTTAAAGATGCTGATACTGTTGCTGGAAGATATGCAGGAAAAATTAGAAGATCAGCATAACGTACATT
TATTAAAAAAGCAACAGCATGATATTATTACATAAAATAAATAAATACATTTACAAGA

>gi|133937136|gb|CT779175.1|CT779175 CT779175 Paramecium tetraurelia conjugation (meiosis and beginning of macronuclear development) Paramecium tetraurelia cDNA clone LK0AEA1YG04 5', mRNA sequence
AGAATCGCCAATGGTGTGATAGGACTTGCCTTGAAAAATTAGGCTTGTTTCAGTGCAGCAAAGAAAGCTAAGATGGAATTGACAATTAGAACTCCATATAGAACAATATTGGA
CAAATTCGAAGGATTCTCAAGAATAGTGGCTAAAACAAATGAAGCAGCTCTCATCATTCAAAATAGAACTCCTGCTGCAGTTTACATATTACCTCCAGGCCCTTTGAAAATCA
AATTCACTTAAGATGTTAAAGGAGTCACAGGGGACTTTTTGCATTTGGGTGGCTATGTGTTTGTGAATCCTGACAATACTTGCGAAATCAATTTGATGGATGTTGTTGATAGA
AAAGAGGCAAAAGTAGATTAATTTGACAAGTCTGATGTTAAAGATGCTGATACTGTTGCTGGAAGATATGCAGGAAAAATTAGAAGATCAGCATAACGTACATTTATTAAAAA
AGCAACAGCATGATATTATTACATAAAATAAATAAATACATTTACAAGA

Putative *T. thermophila* protein has long insert or incorrectly spliced intron.

>gi|118355322|ref|XP_001010921.1| hypothetical protein TTHERM_01094890 [Tetrahymena thermophila SB210]
MFTRFVTQPTLLTQTQRALFSALTKKQKMEVTLRTPYKEYLANFDGFSRITAKTNEASLVIQNKTPASLYVLPPGPLKIRFTSEVKNVSGDFLHTGGWVIVHAQQFNKRQHKN
IHTNIQKFISNLIADLQKDIQQQEKQICNQKKENCEAKVQKFNSQQIKMMNINIIKSTQIDNTCEINVMDLFDRKEVRADQFEKGNIQDLDTLAGKYAAKSRKSTVRLFTKAT
TQ

```
TTHERM_01094890  MFTRFVTQPTLLTQTQRALFSALTKKQKMEVTLRTPYKEYLANFDGFSRITAKTNEASLVIQNKTPASLYVLPPGPLKIRFTSEVKNVSG
PATP00005023001  -MNRIANGVIGLALKNQACFSA-AKKAKMELTIRTPYRTILDKFEGFSRIVAKTNEAALIIQNRTPAAVYILPPGPLKIKFTQDVKGVTG

TTHERM_01094890  DFLHTGGWVIVHAQQFNKRQHKNIHTNIQKFISNLIADLQKDIQQQEKQICNQKKENCEAKVQKFNSQQIKMMNINIIKSTQIDNTCEIN
PATP00005023001  DFLHLGGYVFVNP----------------------------------------------------------------------DNTCEIN

TTHERM_01094890  VMDLFDRKEVRADQFEKGNIQDLDTLAGKYAAKSRKSTVRLFTKATTQ
PATP00005023001  LMDVVDRKEAKVDQFDKSDVKDADTVAGRYAGKIRRSAQRTFIKKATA
```

Smith etal proposed the following corrected version (Smith DG, Gawryluk RM, Spencer DF, Pearlman RE, Siu KW, et al. (2007) Exploring the mitochondrial proteome of the ciliate protozoon *Tetrahymena thermophila*: direct analysis by tandem mass spectrometry. J Mol Biol **374**: 837-863):
>321_26951_X_hypothetical_protein_TTHERM_01094890
MFTRFVTQPTLLTQTQRALFSALTKKQKMEVTLRTPYKEYLANFDGFSRITAKTNEASLVIQNKTPASLYVLPPGPLKIRFTSEVKNVSGDFLHTGGWVISTQIDNTCEINVM
DLFDRKEVRADQFEKGNIQDLDTLAGKYAAKSRKSTVRLFTKATTQ

Existing gene model and proposed revision:

>gene model of GenBank gi|118355321|ref|XM_001010921.1| Tetrahymena thermophila hypothetical protein
TTHERM_01094890
ATGTTCACCAGATTCGTTACTTAGCCAACTTTACTCACCCAAACCCAAAGAGCTTTGTTCTCTGCTTTGACCAAGAAGCAAAAAATGGAAGTTACTTTGAGAACTCCCTACAA
GGAATATTTAGCCAACTTTGACGGCTTCAGCAGAATCACTGCTAAGACCAACGAAGCTTCTTTAGTTATTCAAAATAAAACCCCTGCTTCCTTGTATGTTTTACCTCCTGGTC
CTCTTAAGATTAGATTTACTTCTGAAGTCAAGAATGTTTCAGGTGATTTCCTCCACACTGGTGGTTGGGTCATTGTCCATGCGTAATAATTTAATAAGAGATAACACAAAAAC
ATTCATACAAACATATAAAAATTTATTTCCAATCTGATAGCCGATTTGCAGAAAGATATTCAATAATAGGAAAAACAAATTTGCAATTAAAAAAAGGAAAATTGTGAAGCAAA
AGTCTAAAAATTTAATAGTTAATAAATTAAAATGATGAATATAAATATTATAAAGTCTACACAAATgtatgcgaaattattttcatcttatatcctaaaaaaataatttaaaa
agaattaatgcaatttcgatcttttaaaaacactcgaaatgtacagataatttttttttgagaaaggagctaatagaatgagtaaataaatgaacaaataaaaatatttgat
gttatctagaaaaagatctgatgaatttacatttaaattttcaaaaattaaaactttggaaaagatagattgaaaattactaataaaaatatcaatcgatcgactgatttatg
tggaaataaaaaagatatcacttttattacaatagcaataacaattgcttcgagaaaagatttatatgattaaaattatttattttttataatattaaccaaatttttaact
taaattttaaattaaatt**ag**CGATAATACTTGTGAAATCAATGTTATGGATTTATTCGATAGAAAGGAAGTTAGAGCTGATCAATTCGAAAAGGGTAACATCTAAGATCTTGA
TACTTTGGCTGGTAAATACGCTGCTAAGTCCAGAAAGAGCACTGTTAGACTTTTTACTAAGGCTACCACTTAATGA

Unfortunately, there are no reported *Tetrahymena* EST sequences matching this locus.

Our proposed gene model produces a gene product more closely similar to the *Paramecium* ortholog (and putative *Ichthyophthirius* ortholog, see below).

>revised_NW_001219199.1:49438-50530 [**gene model change** (note: intron at same location in CDS as 2nd intron in PARte ortholog, but larger.)] Tetrahymena thermophila SB210 scf_8254749 genomic scaffold, whole genome shotgun sequence
ATGTTCACCAGATTCGTTACTTAGCCAACTTTACTCACCCAAACCCAAAGAGCTTTGTTCTCTGCTTTGACCAAGAAGCAAAAAATGGAAGTTACTTTGAGAACTCCCTACAA
GGAATATTTAGCCAACTTTGACGGCTTCAGCAGAATCACTGCTAAGACCAACGAAGCTTCTTTAGTTATTCAAAATAAAACCCCTGCTTCCTTGTATGTTTTACCTCCTGGTC
CTCTTAAGATTAGATTTACTTCTGAAGTCAAGAATGTTTCAGGTGATTTCCTCCACACTGGTGGTTGGGTCATTGTCCATGCgtaataatttaataagagataacacaaaaac
attcatacaaacatataaaaatttatttccaatctgatagccgatttgcagaaagatattcaataataggaaaaacaaatttgcaattaaaaaaaggaaaattgtgaagcaaa
agtctaaaaatttaatagttaataaattaaaatgatgaatataaatattataaagtctacacaaatgtatgcgaaattattttcatcttatatcctaaaaaaataatttaaaa
agaattaatgcaatttcgatcttttaaaaacactcgaaatgtacagataatttttttttgagaaaggagctaatagaatgagtaaataaatgaacaaataaaaatatttgat
gttatctagaaaaagatctgatgaatttacatttaaattttcaaaaattaaaactttggaaaagatagattgaaaattactaataaaaatatcaatcgatcgactgatttatg
tggaaataaaaaagatatcacttttattacaatagcaataacaattgcttcgagaaaagatttatatgattaaaattatttattttttataatattaaccaaatttttaact
taaattttaaattaaatt**agCGATAATACTTGTGAAATCAATGTTATGGATTTATTCGATAGAAAGGAAGTTAGAGCTGATCAATTCGAAAAGGGTAACATCTAAGATCTTGA**
TACTTTGGCTGGTAAATACGCTGCTAAGTCCAGAAAGAGCACTGTTAGACTTTTTACTAAGGCTACCACTTAATGA

>TTHERM_01094890_revised_CDS
ATGTTCACCAGATTCGTTACTTAGCCAACTTTACTCACCCAAACCCAAAGAGCTTTGTTCTCTGCTTTGACCAAGAAGCAAAAAATGGAAGTTACTTTGAGAACTCCCTACAA
GGAATATTTAGCCAACTTTGACGGCTTCAGCAGAATCACTGCTAAGACCAACGAAGCTTCTTTAGTTATTCAAAATAAAACCCCTGCTTCCTTGTATGTTTTACCTCCTGGTC
CTCTTAAGATTAGATTTACTTCTGAAGTCAAGAATGTTTCAGGTGATTTCCTCCACACTGGTGGTTGGGTCATTGTCCATGCCGATAATACTTGTGAAATCAATGTTATGGAT
TTATTCGATAGAAAGGAAGTTAGAGCTGATCAATTCGAAAAGGGTAACATCTAAGATCTTGATACTTTGGCTGGTAAATACGCTGCTAAGTCCAGAAAGAGCACTGTTAGACT
TTTTACTAAGGCTACCACTTAATGA

>TTHERM_01094890_revised putative ATP4 F1 delta subunit (158 aa) /
MFTRFVTQPTLLTQTQRALFSALTKKQKMEVTLRTPYKEYLANFDGFSRITAKTNEASLVIQNKTPASLYVLPPGPLKIRFTSEVKNVSGDFLHTGGWVIVHADNTCEINVMD
LFDRKEVRADQFEKGNIQDLDTLAGKYAAKSRKSTVRLFTKATTQ

Queries of PFam database v 24.0. The top match for each ciliate putative **delta** subunit is ATP synthase, Delta/Epsilon chain, beta-sandwich domain (but below the level normally considered "significant"):

header line key:
Family| Description| Entry type| Clan| Envelope{start\end}| Alignment{start\end}| HMM {from\to}| Bit score| E-value|

PARte putative delta-1, E=0.004
ATP-synt_DE_N ATP synthase, Delta/Epsilon chain, beta-sandwich domain Domain n/a 27 112 27 107 1 75 16.5 0.004

```
#HMM       lkleivtpervvfsge..veevvapgeeGefgilpgHaplitaLkpgvlrikted...geeesiavsgGflevq.dnkvti
#MATCH     ++l+i tp r+++++    +++va+++e  + i  +    +  L pg+l+ik ++   g +  +   gG++ v+ dn ++i
#PP        7899**********8668***************99999999***********86524456677888899999995577776
#SEQ       MELTIRTPYRTILDKFegFSRIVAKTNEAALIIQNRTPAAVYILPPGPLKIKFTQdvkGVTGDFLHLGGYVFVNpDNTCEI
```

PARte putative delta-4, E=0. 021

ATP-synt_DE_N ATP synthase, Delta/Epsilon chain, beta-sandwich domain Domain n/a 27 111 27 107 1 75 14.2 0.021

```
#HMM       lkleivtpervvfsge..veevvapgeeGefgilpgHaplitaLkpgvlrikted...geeesiavsgGflevq.dnkvti
#MATCH     ++l++ tp +++ ++    +++va+++e  + i  +    +  L pg+l+ik ++   g +  +   gG++ v+ dn ++i
#PP        78999********98778***************99999999***********86524445577777888888885566655
#SEQ       MELTVRTPYKTIIDKFdgFSRIVAKTNEAALIIQNRTPAAVYILPPGPLKIKFTQdvkGVTGDFLHLGGYVFVNpDNTCEI
```

ICHmu putative delta, E=0.036

ATP-synt_DE_N ATP synthase, Delta/Epsilon chain, beta-sandwich domain Domain n/a 29 114 29 111 1 77 13.5 0.036

```
#HMM       lkleivtpervvfsge..veevvapgeeGefgilpgHaplitaLkpgvlriktedgee...esiavsgGflevq.dnkvtila
#MATCH     ++l++ tp r+v+ +    ++++a+++e  + +  +    +  L pg+l+ik +++ +     +  sgG l ++ dn ++i +
#PP        5678889988876543369*********************************8665442335678888988888778888876
#SEQ       MELTLRTPYREVLVNFdgFSRIQAKTNEAALCVQNKTPASLYVLPPGPLKIKLTQDVKdvsGDYLHSGGWLIIHaDNTCEINV
```

TETth putative delta, E=0.14

ATP-synt_DE_N ATP synthase, Delta/Epsilon chain, beta-sandwich domain Domain n/a 29 114 30 111 2 77 11.6 0.14

```
#HMM       kleivtpervvfsge..veevvapgeeGefgilpgHaplitaLkpgvlriktedgee...esiavsgGflevq.dnkvtila
#MATCH     ++++ tp ++ + +    ++++a+++e ++ i  +    +  L pg+l+i+ +++ +     +  +gG + v+ dn ++i +
#PP        56666776665544335899*******************************7664432234455566666666567777665
#SEQ       EVTLRTPYKEYLANFdgFSRITAKTNEASLVIQNKTPASLYVLPPGPLKIRFTSEVKnvsGDFLHTGGWVIVHaDNTCEINV
```

COMPASS generalized Psi-BLAST alignment profile – profile queries support significance of the similarities to the ATP synthase Delta/Epsilon domain:

Queries using alignment of 4 putative delta subunit sequences from 3 ciliate species (see below).

COMPASS 3.0 Query of PDB70

Job ID: JOB_fkzAWU

Query = Ciliate_F1-deltx.aln
length=158; filtered_length=158; Neff=1.722
(threshold of effective gap content in columns: 0.5)

Database: PDB70_iter5
Effective database length used: 3663686

COMPASS alignment format:
CAPITAL letters: residues at positions aligned by COMPASS (input alignment positions with gap content < 0.5)
lower-case letters: residues at positions not used by COMPASS (input alignment positions with gap content >= 0.5)
'-' : gaps retained from original alignments at positions aligned by COMPASS (positions with gap content < 0.5)
'.' : gaps retained from original alignments at positions not used by COMPASS (positions with gap content >= 0.5)
'=' : gaps introduced by COMPASS in profile-profile alignment
'~' : gaps introduced by COMPASS against positions that are not used in the construction of profile-profile alignment
(positions with gap content >= 0.5)

Clickable sequence names: links to full alignments.

Hits with E-value BETTER than threshold:

Profiles producing significant alignments:		Score	E-value
1E79H	Bovine F1-Atpase Inhibited By Dccd (Dicyclohexylcarbo	107	8.71e-10
2E5YA	F0F1 ATP synthase subunit epsilon [Geobacillus kausto	71	3.31e-03
1AQTA	Molecular Architecture Of The Rotary Motor In Atp Syn	71	3.44e-03
2HLDH	Crystal Structure Of Yeast Mitochondrial F1-Atpasegi	70	3.70e-03

Alignments:

Subject = 1E79H Bovine F1-Atpase Inhibited By Dccd (Dicyclohexylcarbo

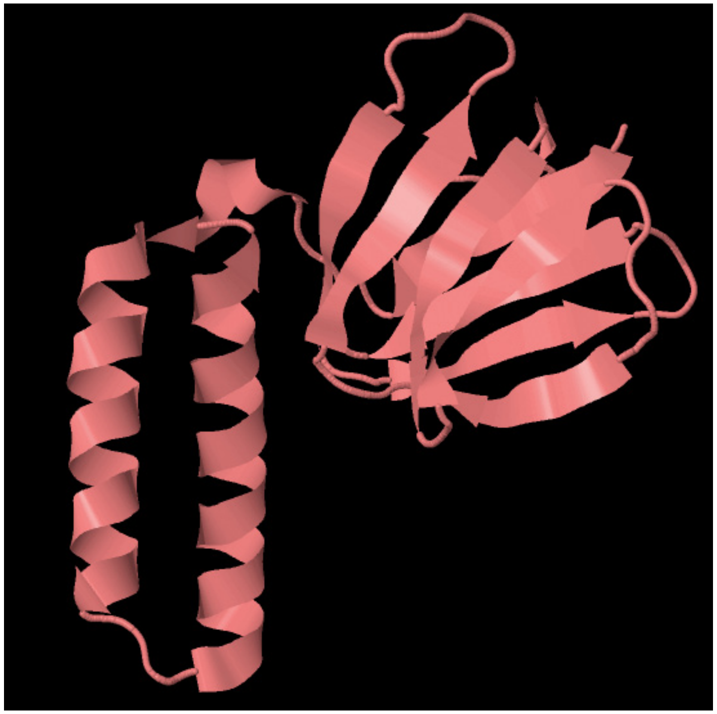

```
length=131  filtered_length=131  Neff=15.893
 Smith-Waterman score = 107   Evalue = 8.71e-10

PARte_delta1   26     KMELTIRTPYRTILDKFEGFSRIVAKTNEAALIIQNRTPAAVYILPPGPLKIKFTQDVKG  85
CONSENSUS_1    28     KMELTLRTPYREVLDNFDGFSRIVAKTNEAALVVQNRTPASLYVLPPGPLKIKLTQDVKG  87
                      K++L++ TP+R+++D+ +  +++V++++E++L ++ +  + L +L PG+L+IK   + +G
CONSENSUS_2    1      KLKLEIVTPERVLFDSGE=VEEVVLPGAEGELGILPGHAPLLTALKPGVLRIK===LADG  56
1E79H          1      QMSFTFASPTQVFFNSAN=VRQVDVPTQTGAFGILAAHVPTLQVLRPGLVVVH===AEDG  56

PARte_delta1   86     VTGDFLHLGGYVFVNPDNTCEINLMDVVDRKEAKVDQFDKSDVKDADTVAGRYAGKIRRS  145
CONSENSUS_1    88     VSGDFLHLGGWLFVHPDNTCEINLLDLFDRKEAKADQFDKGDVKDADTLAGRYAGKSRRS  147
                       +  ++++GG L V+PDN++ I++ +++  +++ +++++++ +++A++++ + + + +R+
CONSENSUS_2    57     EEEKLAVSGGFLEVNPDNVVTILADEAVRAEDIDLERAEEA=LERAEERLADAKDDLDRA  115
1E79H          57     TTSKYFVSSGSVTVNADSSVQLLAEEAVTLDMLDLGAAKAN=LEKAQSELLGAADEATRA  115

PARte_delta1   146    AQRTFIKKATA  156
CONSENSUS_1    148    AQRLFTKAATA  158
                        +L +++A+A
CONSENSUS_2    116    EAQLALAVAEA  126
1E79H          116    EIQIRIEANEA  126
```

COMPASS 3.0 Query of Pfam 23.0

Job ID: ciliate F1-delta

```
Query = RUNBLAST_9reWWW
length=158; filtered_length=158; Neff=1.747
(threshold of effective gap content in columns: 0.5)
```

Database: Pfam 23.0
Effective database length used: 2236890

Hits with E-value BETTER than threshold:

```
Profiles producing significant alignments:                 Score   E-value
ATP-synt_DE_N   ATP synthase, Delta/Epsilon chain, beta-sand  73      1.81e-04
```

Alignments:

Subject = ATP-synt_DE_N ATP synthase, Delta/Epsilon chain, beta-sand

```
length=132  filtered_length=84  Neff=15.060
Smith-Waterman score = 73   Evalue = 1.81e-04

PARte_delta1       27     MELTIRTPYRTILDKFEGFSRIVAKTNEAALIIQNRTPAAVYILPPGPLKIKFTQDVKGV  86
CONSENSUS_1        29     MELTLRTPYREVLDNFDGFSRIVAKTNEAALVVQNRTPASLYVLPPGPLKIKLTQDVKGV  88
                          ++L++ TP+++++D+ D+ +++V++T+E++L+++++   + L +L PG+L+I++++D KG
CONSENSUS_2        1      LKLKVLTPDGLLFDG=DDVKLLVLPTAEGDLGILPGHAPLLTALKPGLLRIRDEDD=KGE  58
Q8M9F2_9ERIC/2-80  1      LNLCVLTPNRIVWDS=E-VKEIILSTNSGQIGVLPNHAPIATAVDIGILRIR-LN-=-DQ  54

PARte_delta1       87     TGDFLHLGGYVFVNPDNTCEINLMDV  112
CONSENSUS_1        89     SGDFLHLGGWLFVHPDNTCEINLLDL  114
                          +  +  +GG+L+V+++++++ I++ ++
CONSENSUS_2        59     TELIFLSGGFLEVSGGDEVTILAEEA  84
Q8M9F2_9ERIC/2-80  55     WLTMALMGGFARIG-NNDITVLVNDA  79
```

```
Parameters:
E-value cutoff: 1.000e+00
Maximal number of hits to display: 100
Lambda_ungapped: 3.4578e-01
Expected value of positional scores: -2.8043e-01
Gap opening: 10
Gap extension: 1
```

COMPASS 3.0 Query of KOG

Job ID: ciliate F1 delta

```
Query = RUNBLAST_wh4zX9
length=158; filtered_length=158; Neff=1.747
(threshold of effective gap content in columns: 0.5)
```

Database: KOG
Effective database length used: 2777205

Hits with E-value BETTER than threshold:

Profiles producing significant alignments:	Score	E-value
KOG1758 Mitochondrial F1F0-ATP synthase, subunit delta/ATP	94	5.85e-07

Hits with E-value WORSE than threshold (may be not biologically meaningful):

KOG2090 Metalloendopeptidase family - mitochondrial interm	64	7.68e-01

Alignments:

Subject = KOG1758 Mitochondrial F1F0-ATP synthase, subunit delta/ATP

```
length=207  filtered_length=165  Neff=3.788
Smith-Waterman score = 94   Evalue = 5.85e-07

PARte_delta1    21     -AAKKAKMELTIRTPYRTILDKFEGFSRIVAKTNEAALIIQNRTPAAVYILPPGPLKIKF  79
CONSENSUS_1     22     ALAKKAKMELTLRTPYREVLDNFDGFSRIVAKTNEAALVVQNRTPASLYVLPPGPLKIKL  81
                       ++A   K+ LT+ +P+++V+D+ +     +    ++   ++ V   +     + VL PG ++++
CONSENSUS_2     32     PAAGPEKLKLTFALPNQTVYDGAE=VKQVDLPALSGQIGVLANHVPTIAVLKPGVVSVH=  89
AtCh029         1      -----MTLNLCVLTPNRIVWD-SE=VKEIILSTNSGQIGVLANHAPIATAVDIGILKIR=  52

PARte_delta1    80     TQDVKGVTGDFLHLGGYVFVNPDNTCEINLMDVVDRKEAKVDQFDKSDVK  129
CONSENSUS_1     82     TQDVKGVSGDFLHLGGWLFVHPDNTCEINLLDLFDRKEAKADQFDKGDVK  131
                         D  G +  F+ +GG+++V+PD+ + I + +++      K++ +D +++K
CONSENSUS_2     90     ==DEGGNVLKFFLSGGFATVNPDSELQILAEEAL=====KLEDIDPSEAK  132
AtCh029         53     ==-LANQWLTMALMGGFARIG-NNEITILVNDAE=====KNSDIDPQEAQ  93
```

```
--------------------------------------------------------------------
```

Almost complete partial sequence of ortholog from cDNA data of the ciliate *Ichthyophthirius multifiliis*.
2 cDNAs:

```
>gi|133881319|gb|EL910355.1|EL910355 INIT2_39_E06.b1_A006 G5 trophont cDNA (INIT2) Ichthyophthirius multifiliis
cDNA clone INIT2_39_E06_A006 5', mRNA sequence
TGTTTTTTTGATAAAATAAAATTAAATAATTATAATATAAATATATATAAAAGAAAAACAAATGTTCACTAAATTATTTTTGAACCAATCTCATATAACTTAATTATAAAAAT
AATATTTCTCCTAATTAGCAAAAAAAGCAAAAATGGAGCTAACTTTAAGAACTCCATATAGAGAAGTATTAGTTAATTTTGATGGTTTTTCAAGAATTTAAGCCAAAACAAAT
GAAGCCGCTTTATGTGTTTAAAATAAAACTCCAGCATCTTTATATGTTTTACCACCTGGTCCTTTAAAAATAAAACTTACATAAGATGTCAAAGATGTATCAGGAGATTATCT
TCATAGCGGTGGTTGGGTAATTATACATGCAGATAACACTTGTGAAATTAATGTTAT
Orf in reading frame 2:
VFLIKQNQIIIIQIYIKEKQMFTKLFLNQSHITQLQKQYFSQLAKKAKMELTLRTPYREVLVNFDGFSRIQAKTNEAALCVQNKTPASLYVLPPGPLKIKLTQDVKDVSGDY
LHSGGWVIIHADNTCEINV…

>gi|133879978|gb|EL909014.1|EL909014 INIT2_28_E06.g1_A006 G5 trophont cDNA (INIT2) Ichthyophthirius multifiliis
cDNA clone INIT2_28_E06_A006 3', mRNA sequence
AAAAAAAAAAAAGAGAAGTATTAGTTAATTTTGATGGTTTTTCAAGAATTTAAGCCAAAACAAATGAAGCCGCTTTATGTGTTTAAAATAAAACTCCAGCATCTTTATATGTT
TTACCACCTGGTCCTTTAAAAATAAAACTTACATAAGATGTCAAAGATGTATCAGGAGATTATCTTCATAGCGGTGGTTGGTTAATTATACATGCAGATAACACTTGTGAAAT
TAATGTTATGGATTTATTTGAAAGAAAGGAAGTTAAGGCTGATTAATTTGATAAAGCAAATATTGTTGATGCAGATACTAACGCAGGTAAATATGCATAAAAATCAAGAAAAA
ATACTAACAGAATATTTTTTC
Orf in reading frame 3:
KKKREVLVNFDGFSRIQAKTNEAALCVQNKTPASLYVLPPGPLKIKLTQDVKDVSGDYLHSGGWLIIHADNTCEINVMDLFERKEVKADQFDKANIVDADTNAGKYAQKSRKN
TNRIFF
```

Overlap:
VFLIKQNQIIIIQIYIKEKQMFTKLFLNQSHITQLQKQYFSQLAKKAKMELTLRTPYREVLVNFDGFSRIQAKTNEAALCVQNKTPASLYVLPPGPLKIKLTQDVKDVSGDY
~~KKK~~REVLVNFDGFSRIQAKTNEAALCVQNKTPASLYVLPPGPLKIKLTQDVKDVSGDY
LHSGGWVIIHADNTCEINV…
LHSGGWLIIHADNTCEINVMDLFERKEVKADQFDKANIVDADTNAGKYAQKSRKNTNRIFF…

Combination—*I. multifiliis* putative Delta subunit evidently missing only a few amino acids at C-terminus.
MFTKLFLNQSHITQLQKQYFSQLAKKAKMELTLRTPYREVLVNFDGFSRIQAKTNEAALCVQNKTPASLYVLPPGPLKIKLTQDVKDVSGDYLHSGGWLIIHADNTCEINVMD
LFERKEVKADQFDKANIVDADTNAGKYAQKSRKNTNRIFF…

The 4 Paramecium paralogues:

>gi|145480051|ref|XP_001426048.1|A0BJD3_PARTE| hypothetical protein GSPATT00005023001 [Paramecium tetraurelia strain d4-2]
MNRIANGVIGLALKNQACFSAAKKAKMELTIRTPYRTILDKFEGFSRIVAKTNEAALIIQNRTPAAVYILPPGPLKIKFTQDVKGVTGDFLHLGGYVFVNPDNTCEINLMDVV
DRKEAKVDQFDKSDVKDADTVAGRYAGKIRRSAQRTFIKKATA

>gi|145540206|ref|XP_001455793.1|A0DZC9_PARTE| hypothetical protein GSPATT00003365001 [Paramecium tetraurelia strain d4-2]
MNRIANGVIGLALKNQACFSTAKKAKMELTIRTPYRTILDKFEGFSRIVAKTNEAALIIQNRTPAAVYILPPGPLKIKFTQDVKGVTGDFLHLGGYVFVNPDNTCEINLMDVV
DRKEAKVDQFDKADVKDADTVAGRYAGKIRRSAQRTFIKKATA

>gi|145541926|ref|XP_001456651.1|A0E1T7_PARTE| hypothetical protein GSPATT00022425001 [Paramecium tetraurelia strain d4-2]
MNRITNGVIGLAFKNSFFFSAAKKAKMELTVRTPYKTIIDKFDGFSRIVAKTNEAALIIQNRTPAAVYILPPGPLKIKFTQDVKGVTGDFLHLGGYVFVNPDNTCEINLLDVV
DRKEAKVDQFDKADVKDADTVAGRYAGKIRRAAQRTFIKKATA

>gi|145536381|ref|XP_001453918.1|A0DU04_PARTE| hypothetical protein GSPATT00020205001 [Paramecium tetraurelia strain d4-2]
MNRLTNGVIGLAIKNSFFFSTAKKAKMELTVRTPYKTIIDKFDGFSRIVAKTNEAALIIQNRTPAAVYILPPGPLKIKFTQDVKGVTGDFLHLGGYVFVNPDNTCEINLLDVV
DRKEAKVDQFDKADVKDADTVAGRYAGKIRRAAQRTFIKKATA

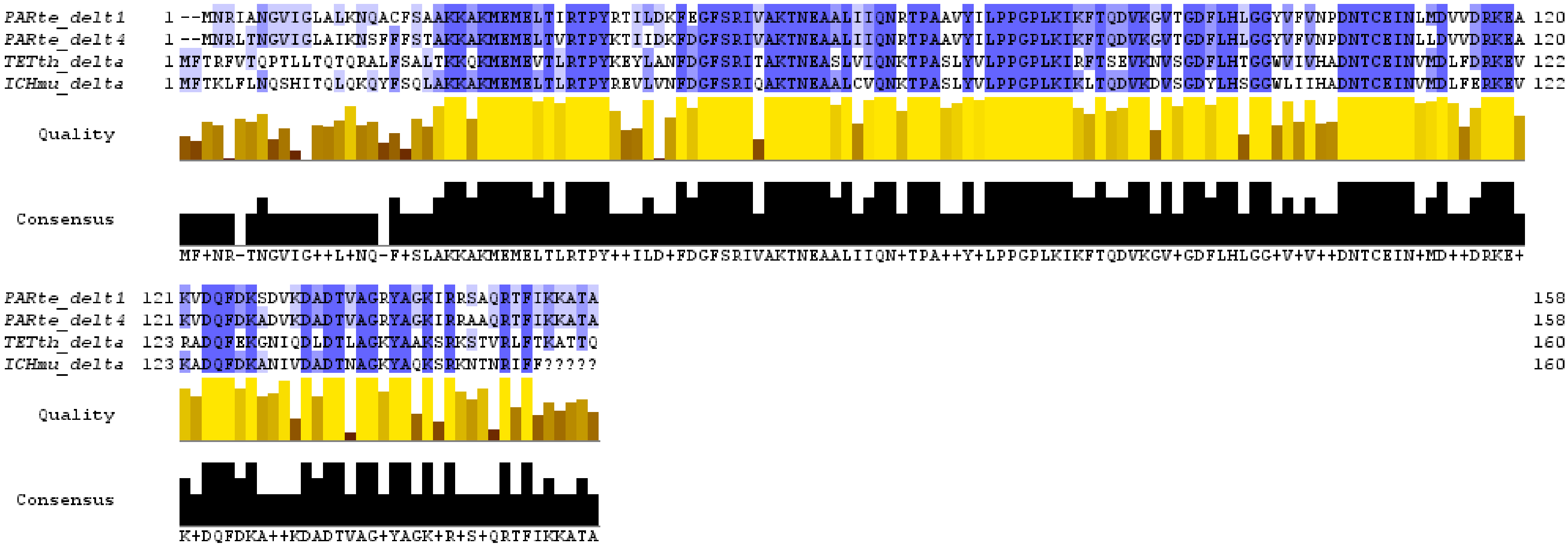

Alignment of ciliate F_1 delta subunit sequences, colored according to per cent identity in Jalview. Species identified by Swissprot/Uniprot abbreviation style (TETth: *Tetrahymena thermophila*).

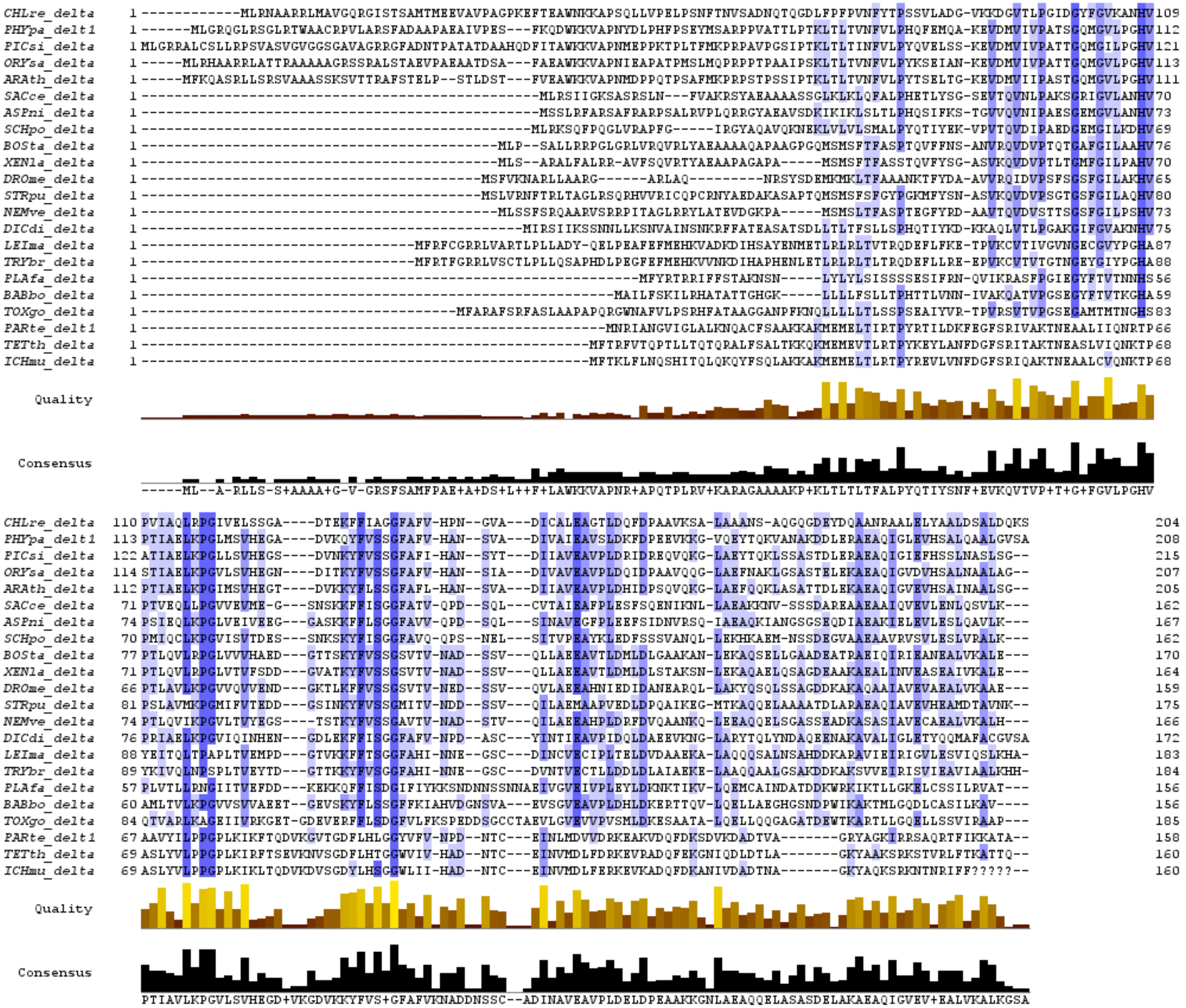

Alignment of 3 putative ciliate F_1 delta subunits with 19 other Eukaryotic delta sequences.

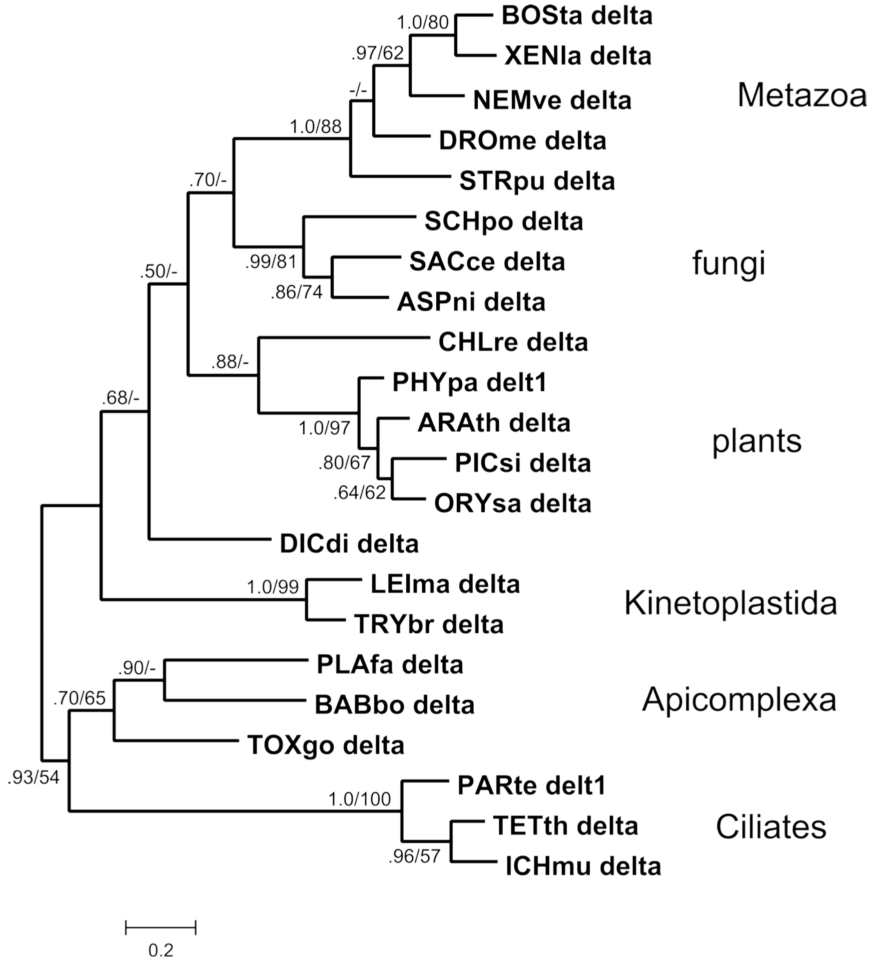

Phylogram resulting from phylogenetic analysis by Bayesian inference. Similar results were obtained by maximum likelihood analysis. Numbers near branch nodes indicate Bayesian posterior probabilities/maximum likelihood bootstrap support; - indicates Bayesian inference probability of less than 0.5 or maximum likelihood support of less than 50%.
