# Supplementary material for: Highly Divergent Mitochondrial ATP Synthase Complexes in Tetrahymena thermophila
Source: PLoS Biol. 2010 Jul 13;8(7):e1000418. doi: 10.1371/journal.pbio.1000418 (PMC2903591; doi:10.1371/journal.pbio.1000418)
Supplement: Text S2 — Supplemental results: proposed revision of gi:89295266/trembl:Q239R1/Hypothetical protein TTHERM_01188360 and identification as putative T. thermophila ATP synthase Fo d subunit. (2.17 MB PDF) [file pbio.1000418.s012.pdf]

Text S2: Supplemental Results:

Proposed revision of gi:89295266/trembl:Q239R1/Hypothetical protein TTHERM_01188360 and Identification as putative *T. thermophila* ATP synthase F_O d subunit

Index--

Summary of revision
Existing gene model and proposed revision
Three *Paramecium* paralogues
Putative *Ichthyophthirius multifiliis* ortholog
Alignment of ciliate sequences
Results of BLAST searches
Results of PFam queries
COMPASS searches and statistical significance of similarity
Alignment of d subunit sequences
Tree resulting from phylogenetic analysis of d subunits

Summary of revision:

>gi|89295266|gb|EAR93254.1| hypothetical protein TTHERM_01188360 [Tetrahymena thermophila SB210] 162 aa
MSMLAKIAKNVVKTQALKNTTAAQTPSFQAPGNQDKILKWISTLSNKATTGESRSYCTQLSSLVSFYNKQHVEQIPTIDFNEWKSVISTQGLVDKVKENYESLIKEQY
NTDAISKQISSASSKALDDIENELSFHAAIWLNAYADYTMFLFELEEYNDPNVP

>TTHERM_01188360_revised (putative ATP synthase F_o d subunit) 234 aa
MSMLAKIAKNVVKTQALKNTTAAQTPSFQAPGNQDKILKWISTLSNKATTGESRSYCTQLSSLVSFYNKQHVEQIPTIDFNEWKSVISTQGLVDKVKENYESLIKEQYNTDAI
SKQISSASSKALDDIENELSFHAAIWLNAYADYTMFLFELEEYNDPNDYLMHENFDFFRGLETELEELTETHNYIPGAKDDVNLRGYLATQFAWGKKVISFYRHPADDFKCAK
ATKNMLGR

Existing gene models:

Gene models presently in NCBI for two predicted genes TTHERM_01188360 and TTHERM_01188370 separated by 102 bp on Tetrahymena thermophila SB210 genomic scaffold scf_8254676. (Exons, uppercase blue letters; introns, lowercase brown letters.)

aattaaaaattaagaaacaaaataaaaggagaaaatatcaaataaaaatagatataaaaATGTCCATGTTAGCTAAGATTGCCAAGAATGTTGTTAAGACTTAAGCTCTTAA
GAACACCACTGCAGCTTAAACCCCTTCTTTCTAAGCTCgtatagattttattcatcctgatttgatcaagcaaaagcacttcttcaaatgaaaaagcaagcaaaaagcagaa
gcgaaattattaaatttgaagctatcatatcatcaaaattattatcactaaaataagctcttaaaatgaattatagatttaaattttcagatattatgttcaagagaccaaat
tttattatgatgcaagttaggttttaagatgcaaacattttagatagcaattagtagataaagagcggctttttagcgattcctttaaataatttttcatcgaaaaaataatt
tattgtgaattaattaagatgaagcaggaaataaatagagcttgttcgaagattttgctttttgctttgataaaataagataagttttatgtgagatattaattgttattta
tgttataaattatagCTGGTAACCAAGACAAAATCCTTAAGTGGATCTCTACCTTGAGCAACAAGGCCACTACTGGTGAATCTAGATCATACTGCACTCAACTTTCTTCTCTT
GTCAGTTTCTACAACAAGCAACACGTTGAATAAATCCCTgtaagcacagcataaaatgaatgaaaaatattaaaaataaataatatctttttaaactggccaaatagtctaag
caaaattttatttagttagaataactagcaaaaatgagattaggaaatttatagaaatcgtttagcagtttaaagttagaaattaataaataaataaatatctaaagctaaga
atttatttaatatcttttgcaaatctatataaaagatcaagaaatagcataaaacactaatgaaagcaatttattttataaaatttgatttcttatgaaaatcttcattgaaa
ttacttaaaaaaacttattttggcaaattatatttgatattaattaatgtcatcttattcatattattttcattaaaaaagACCATCGATTTCAATGAATGGAAGAGTGTCAT
CTCCACCCAAGGTTTGGTTGATAAGGTTAAGGAAAACTACGAATCTTTGATTAAGGAACAATACAACACTGATGCTATCTCCAAGCAAATCAGTTCTGCTTCCTCTAAGGCTC
TCGACGATATTgtaaattgataaaacaagttcaattatatatttctaaaatcttttattattctacagGAAAACGAACTCTCCTTCCACGCTGCTATCTGGTTGAATGCTTAC
GCTGACTACACCATGTTCCTTTTCGAACTTGAAGAATACAACGACCCCAATGTACCTTGAatttcctcagctttattattattatcatataccaaaaaagaaaaatatgcaaa
tttcaaggcattgaaaaatcaagcaaataaaagctgcttgctatataacATGAATTAATTGCATATTTATTTATAAGCAAAAATAATACTTTTATTTGATAAAATAAAGGACT
ACCTCATGCATGAAAACTTCGATTTCTTCAGAGGTCTTGAAACTGAAgtaattatttattataaaatttgataggaaaaacataatttaaattatatattttttttaattaata
aagctcgaagaattaaccgaaagtatagattttattcattgattaaacagagaaattagaaaatattttaagcttgaaacaatttaaaatgaatataattcaaagTTTCTCAT
TTTTTGTTAAAATAAAATAGCCCACAACTACATTCCCGGTGCTAAGGATGACGTCAACTTGAGAGGTTATCTTGCCACTCAgtaaataatttaaaaattttttattttcaatct
gtttgtttgcttgacttcttgattattcgatcaagcaaagcagataaaatttatttttttaagttatttaaataacatatttaatctctttaatttaaaatagATTCGCTTGG
GGTAAAAAAGTTATCAGgtaaacatttttgttctttttatgatggaagtatttgaaatagaatcagttataatacatacatagattacatagatgataaaattgattgtttaa
attagcatgtatcaatattttattgtataaatagTTTCTATCGTCACCCTGCTGATGATTTCAAGTGTGCTAAGGCCACCAAGAATATGTTAGGTAGATGAgctgccctttct
tacctaaataacaaatttaatttaaaaaaactactgcttttaataatg

Gene model revisions:

Smith etal (Smith DG, Gawryluk RM, Spencer DF, Pearlman RE, Siu KW, et al. (2007) Exploring the mitochondrial proteome of the ciliate protozoon Tetrahymena thermophila: direct analysis by tandem mass spectrometry. J Mol Biol 374: 837-863) **proposed a revision** [327a/b] containing most of TTHERM_01188360 + TTHERM_01188370 (matching cDNA/ESTs were not available at that time):

+ >gi|89295266|gb|EAR93254.1| hypothetical protein TTHERM_01188360 [Tetrahymena thermophila SB210]
MSMLAKIAKNVVKTQALKNTTAAQTPSFQAPGNQDKILKWISTLSNKATTGESRSYCTQLSSLVSFYNKQHVEQIPTIDFNEWKSVISTQGLVDKVKENYESLIKE
QYNTDAISKQISSASSKALDDIENELSFHAAIWLNAYADYTMFLFELEEYNDPNVP
+ >gi|146164582|ref|XP_001013500.2| hypothetical protein TTHERM_01188370 [Tetrahymena thermophila]
MNQLHIYLQAKIILLFDKIKDYLMHENFDFFRGLETEFLIFCQNKIAHNYIPGAKDDVNLRGYLATQFAWGKKVISFYRHPADDFKCAKATKNMLGR

= >327a/b TTHERM_01188360+TTHERM_01188370 237 aa
MSMLAKIAKNVVKTQALKNTTAAQTPSFQAPGNQDKILKWISTLSNKATTGESRSYCTQLSSLVSFYNKQHVEQIPTIDFNEWKSVISTQGLVDKVKENYESLIKE
QYNTDAISKQISSASSKALDDIENELSFHAAIWLNAYADYTMFLFELEEYNDPNDYLMHENFDFFRGLETEFLIFCQNKIAHNYIPGAKDDVNLRGYLATQFAW
GKKVISFYRHPADDFKCAKATKNMLGR

Further correction using newer cDNA sequences--
One of 2 identical cDNA sequences that extend past the existing predicted start of the second gene (matching the 5’ 80% of the revised CDS):

>gi|18194228|gb|BM394175.1|BM394175 50072-2-2-D04.f.1 Chilcoat/Turkewitz cDNA (large fraction) Tetrahymena thermophila cDNA. (Extent of exons deduced in the genomic DNA shown by alternating violet and light blue coloration)
AGAAACAAATAAAGGAGAAAATATCAAATAAAAATAGATATAAAAATGTCCATGTTAGCTAAGATTGCCAAGAATGTTGTTAAGACTTAAGCTCTTAAGAACACCACTGCAGC
TTAAACCCCTTCTTTCTAAGCTCCTGGTAACCAAGACAAAATCCTTAAGTGGATCTCTACCTTGAGCAACAAGGCCACTACTGGTGAATCTAGATCATACTGCACTCAACTTT
CTTCTCTTGTCAGTTTCTACAACAAGCAACACGTTGAATAAATCCCTACCATCGATTTCAATGAATGGAAGAGTGTCATCTCCACCCAAGGTTTGGTTGATAAGGTTAAGGAA
AACTACGAATCTTTGATTAAGGAACAATACAACACTGATGCTATCTCCAAGCAAATCAGTTCTGCTTCCTCTAAGGCTCTCGACGATATTGAAAACGAACTCTCCTTCCACGC
TGCTATCTGGTTGAATGCTTACGCTGACTACACCATGTTCCTTTTCGAACTTGAAGAATACAACGACCCCAATGACTACCTCATGCATGAAAACTTCGATTTCTTCAGAGGTC
TTGAAACTGAACTCGA_apparent_end_of_exact_matches_to_gDNA_GGAATAACCGAACCCACAACTACGTTCCCG

Revised gene model based on the cDNAs plus guidance from homology to 3 other ciliate coding sequences at the 3’ end (which sequences are fully supported by cDNAs from those spp.).
aattaaaaaattaagaaacaaaataaaaggagaaaatatcaaataaaaatagatataaaaATGTCCATGTTAGCTAAGATTGCCAAGAATGTTGTTAAGACTTAAGCTC
TTAAGAACACCACTGCAGCTTAAACCCCTTCTTTCTAAGCTCgtatagatttttattcatcctgatttgatcaagcaaaagcacttcttcaaatgaaaaagca
agcaaaaagcagaagcgaaattattaaatttgaagctatcatatcatcaaaattattatcactaaaataagctcttaaaatgaattatagatttaaattttca
gatattatgttcaagagaccaaattttattatgatgcaagttaggttttaagatgcaaacattttagatagcaattagtagataaagagcggctttttagcga
ttcctttaaataatttttcatcgaaaaaataatttattgtgaattaattaagatgaagcaggaaataaatagagcttgttcgaagattttgctttttgctttg
ataaaataagataagttttatgtgagatattaattgttattttatgttataaattatagCTGGTAACCAAGACAAAATCCTTAAGTGGATCTCTACCTTGAGC
AACAAGGCCACTACTGGTGAATCTAGATCATACTGCACTCAACTTTCTTCTCTTGTCAGTTTCTACAACAAGCAACACGTTGAATAAATCCCTgtaagcacag
cataaaatgaatgaaaaatattaaaaataaataatatctttttaaactggccaaatagtctaagcaaaattttatttagttagaataactagcaaaaatgaga
ttaggaaatttatagaaatcgtttagcagtttaaagttagaaattaataaataaataaatatctaaagctaagaatttatttaatatcttttgcaaatctata
taaaagatcaagaaatagcataaaacactaatgaaagcaatttattttataaaatttgatttcttatgaaaatcttcattgaaattacttaaaaaaacttatt
ttggcaaattatatttgatattaattaatgtcatcttattcatattattttcattaaaaaagACCATCGATTTCAATGAATGGAAGAGTGTCATCTCCACCCA
AGGTTTGGTTGATAAGGTTAAGGAAAACTACGAATCTTTGATTAAGGAACAATACAACACTGATGCTATCTCCAAGCAAATCAGTTCTGCTTCCTCTAAGGCT
CTCGACGATATTgtaaattgataaaacaagttcaattatatatttctaaaatcttttattattctacagGAAAACGAACTCTCCTTCCACGCTGCTATCTGGT
TGAATGCTTACGCTGACTACACCATGTTCCTTTTCGAACTTGAAGAATACAACGACCCCAATgtaccttgaatttcctcagctttattattattatcatatac
caaaaaagaaaaatatgcaaatttcaaggcattgaaaaatcaagcaaataaaagctgcttgctatataacatgaattaattgcatatttatttataagcaaaa

ataatacttttatttgataaaataaagGACTACCTCATGCATGAAAACTTCGATTTCTTCAGAGGTCTTGAAACTGAAgtaattatttattataaaatttgat
aggaaaaacataatttaaattatatatttttttaattaataaagCTCGAAGAATTAACCGAAAgtatagattttattcattgattaaacagagaaattagaaa
atattttaagcttgaaacaatttaaaatgaatataattcaaagtttctcatttttttgttaaaataaaatagCCCACAACTACATTCCCGGTGCTAAGGATGAC
GTCAACTTGAGAGGTTATCTTGCCACTCAgtaaataatttaaaaatttttattttcaatctgtttgtttgcttgacttcttgattattcgatcaagcaaagca
gataaaatttattttttttaagttatttaaataacatatttaatctctttaatttaaaatagATTCGCTTGGGGTAAAAAAGTTATCAGgtaaacatttttgtt
cttttatgatggaagtatttgaaatagaatcagttataatacatacatagattacatagatgataaaattgattgtttaaattagcatgtatcaatatttta
ttgtataaatagTTTCTATCGTCACCCTGCTGATGATTTCAAGTGTGCTAAGGCCACCAAGAATATGTTAGGTAGATGAgctgccctttcttacctaaataacaaa
tttaatttaaaaaaactactgcttttaataatg

>deduced **TTHERM_01188360_revised** CDS
ATGTCCATGTTAGCTAAGATTGCCAAGAATGTTGTTAAGACTTAAGCTCTTAAGAACACCACTGCAGCTTAAACCCCTTCTTTCTAAGCTCCTGGTAACCAAGACAAA
ATCCTTAAGTGGATCTCTACCTTGAGCAACAAGGCCACTACTGGTGAATCTAGATCATACTGCACTCAACTTTCTTCTCTTGTCAGTTTCTACAACAAGCAACACGTT
GAATAAATCCCTACCATCGATTTCAATGAATGGAAGAGTGTCATCTCCACCCAAGGTTTGGTTGATAAGGTTAAGGAAAACTACGAATCTTTGATTAAGGAACAATAC
AACACTGATGCTATCTCCAAGCAAATCAGTTCTGCTTCCTCTAAGGCTCTCGACGATATTGAAAACGAACTCTCCTTCCACGCTGCTATCTGGTTGAATGCTTACGCT
GACTACACCATGTTCCTTTTCGAACTTGAAGAATACAACGACCCCAATGACTACCTCATGCATGAAAACTTCGATTTCTTCAGAGGTCTTGAAACTGAACTCGAAGAA
TTAACCGAAACCCACAACTACATTCCCGGTGCTAAGGATGACGTCAACTTGAGAGGTTATCTTGCCACTCAATTCGCTTGGGGTAAAAAAGTTATCAGTTTCTATCGT
CACCCTGCTGATGATTTCAAGTGTGCTAAGGCCACCAAGAATATGTTAGGTAGATGA

>Predicted protein TTHERM_01188360_revised 234 aa
MSMLAKIAKNVVKTQALKNTTAAQTPSFQAPGNQDKILKWISTLSNKATTGESRSYCTQLSSLVSFYNKQHVEQIPTIDFNEWKSVISTQGLVDKVKENYESLIKEQYNTDAI
SKQISSASSKALDDIENELSFHAAIWLNAYADYTMFLFELEEYNDPNDYLMHENFDFFRGLETELEELTETHNYIPGAKDDVNLRGYLATQFAWGKKVISFYRHPADDFKCAK
ATKNMLGR

(MitoProt II PROBABILITY of export to mitochondria: **0.6914)**

***Paramecium* orthologues**:

>gi|145536582|ref|XP_001454013.1| hypothetical protein [Paramecium tetraurelia strain d4-2] 222 aa
MRRLIVNQTRSKTVAARPSANLDRINKWLQTLSAKANTLESRFYASQLSSLFNFYSKPSTGAAQEIDWNYWKEQITTEGLVDKVQKGHDTLLQREFDVERICHQVVSSQSKEL
EDLENELTFHSAVWSNYYLDQHLALLDLEQYGDRNSYVIHEDYDFYPGLEADLEELTETHNWIPGSKDDINLKGYMVSQFQWGKKIISFYRHPCDDFKAARGTKNILGR
(MitoProt II PROBABILITY of export to mitochondria: **0.9813)**

>gi|145538341|ref|XP_001454876.1| hypothetical protein [Paramecium tetraurelia strain d4-2] 222 aa
MRRLIVNQTRSKTVAARPSANLDRINKWLQTLTAKANTLESRFYTSQLSSLFNFYSKPTTGAAQEIDWNYWKEQITTEGLVDKVQKGHETLLNKEFDVERICHQVVSSQSKEL
EDLENELTFHSAVWSNYYLDQHLALLDLEQYGDRNSYVIHEDYDFYPGLEADLEELTETHNWIPGSKDDINLKGYMVSQFQWGKKIISFYRHPCDDFKAARGTKNILGR

>gi|145544122|ref|XP_001457746.1| hypothetical protein [Paramecium tetraurelia strain d4-2]
MRRLIVNQTRSKTVAARPSANLDRINKWLQTLSAKANTLESRFYASQLSSLFNFYSKPSTGAAQEIDWNHWKEQITTEGLVDKVQKGHDTLLQREFDVERICHQVVSSQSKEL
EDLENELTFHSAVWSNYYLDQHLALLDLEQYGDRNSYVIHEDYDFYPRIRSRLRRIDRNSQLDSRFKG

Putative ortholog from another ciliate: *Ichthyophthirius multifiliis* cDNA

>gi|133878779|gb|EL907815.1|EL907815 INIT2_21_D04.b1_A006 G5 trophont cDNA (INIT2) Ichthyophthirius multifiliis cDNA clone INIT2_21_D04_A006 5', mRNA sequence
AAAAAAAAATAAATCAATAAAAACAAATGTCGTTCTTCTCAGTAATTGCTAAAAATAAAGTTTCAAATTAATCCTTAAGAAATGCTGTTACTGCATTAAAGCCATCTTAAAAT
CAAGAAATAATTACAAAATGGATATAAACCTTAAATAGCAAAGCAAGTTCAGCTGAATCCCGTTCATATTGTGCTTAACTTTCATCTTTAATAAGTTATTACAATAGATAACA
CACTGAAAAAATTCCAACCATTAATTGGGAAGAATGGAAAAAATAGATTAGCACAAAAGGATTAGTAGAAAAAGTAAAAGAGAATTATGAAACTTTAATAAGAGAATAATACC
AAGTTGATTAGATTGCTAAATAAGTTTTATCTTAAACATCTAAGCCTCTCGATGATATTGAAAATGAATTATCTTTCCATGCTGCTATTTGGTTAAATGCTTATTCTGATTAT
ACTATGTTTTTATTTGAACTAGAAGAATATAATAATCCTAATGAATACCTAATGCATGAAAACTATGACTTCTTTAAAGGATTAGAAGCTGAACTTGAAGAATTAACTGAAAC
ACATAACTATATTCCTGGTTCAAAGGACGATGTTAACCTTAGAGGATATTTAGCATGCTAATTTGCTTGGGGTAAGAAAGTTATTAGTTTCTATCGTCATCCTTCTGATGATT
TTAAATGCGCAAAAGCCACAAAAAAATATGCTTGGTAGATGATTTATTTTTTATTTAGACTTATTAAGATAAGAATATAAATATTAAATAACACCTTACTTATATGAAAAAATA
AATAATAATTTTTTGTTATAAATAAAAGTAAATGTTATTTATAAATATTTAAAATAAAAAATATTAAAAT

Translation of **Ichthyophthirius multifiliis cDNA clone INIT2_21_D04_A006**
KKINQQKQMSFFSVIAKNKVSNQSLRNAVTALKPSQNQEIITKWIQTLNSKASSAESRSYCAQLSSLISYYNRQHTEKIPTINWEEWKKQISTKGLVEKVKENYETLIREQYQ
VDQIAKQVLSQTSKPLDDIENELSFHAAIWLNAYSDYTMFLFELEEYNNPNEYLMHENYDFFKGLEAELEELTETHNYIPGSKDDVNLRGYLACQFAWGKKVISFYRHPSDDF
KCAKATKNMLGR*FIFYLDLLRQEYKYQITPYLYEKINNNFLLQIKVNVIYKYLKQKILK

(MitoProt II PROBABILITY of export to mitochondria: **0.9458)**

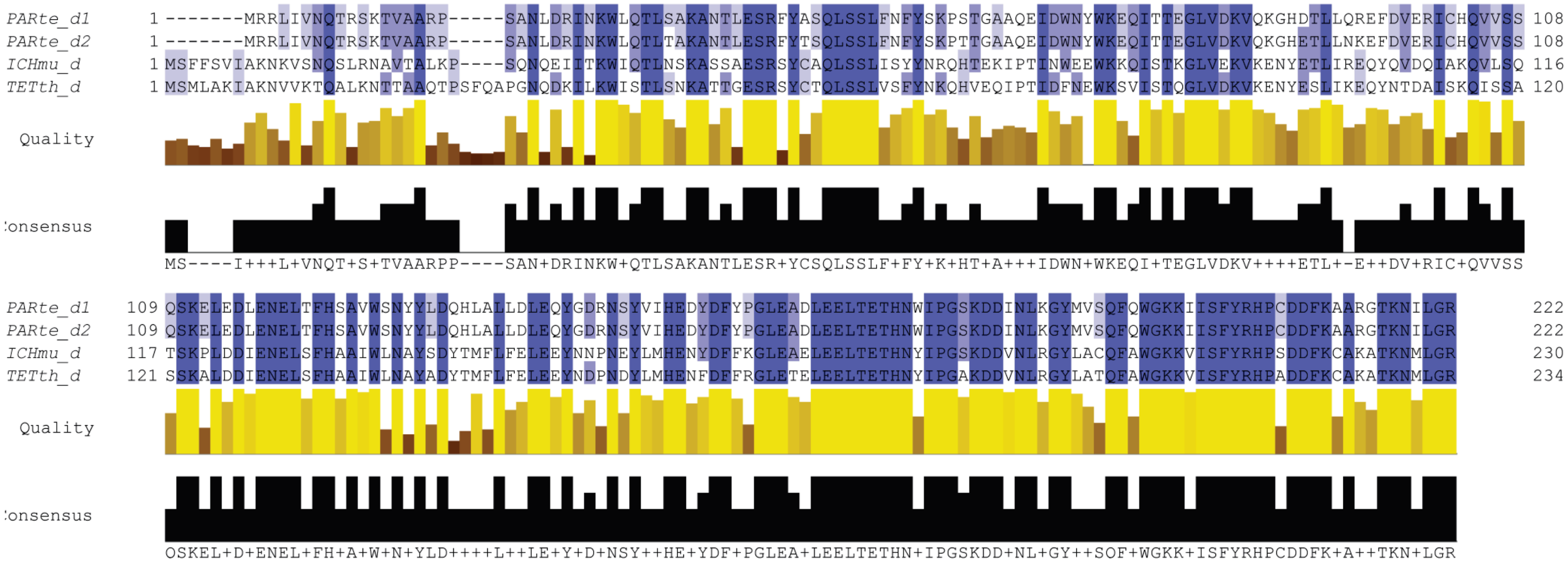

Alignment of putative subunits **d** from 3 ciliate spp., highlighted by Jalview according to % identity.

Blast Results.

BLAST query at NCBI with the revised TTHERM_01188360 pulls up hits to additional putative ATP **d** subunits (as well as helicases and GHMP kinases), but with E-values above the level normally considered significant. The first two non-ciliate matches, *Monosiga* and *Phytophthora*, both contain putative Pfam ATP synthase D chain domains.

Sequences producing significant alignments:		Score (Bits)	E Value
ref|XP_001013499.1|	hypothetical protein TTHERM_01188360 [Tet...	327	7e-88
ref|XP_001454013.1|	hypothetical protein [Paramecium tetraure...	256	9e-67
ref|XP_001454876.1|	hypothetical protein [Paramecium tetraure...	256	2e-66
ref|XP_001457746.1|	hypothetical protein [Paramecium tetraure...	151	4e-35
ref|XP_001013500.2|	hypothetical protein TTHERM_01188370 [Tet...	145	2e-33
ref|XP_001750450.1|	hypothetical protein [Monosiga brevicolli...	43.1	0.023

```
gb|EEY55610.1|  conserved hypothetical protein [Phytophthora i...  37.0   1.8
ref|YP_812094.1|  ATP-dependent DNA helicase RecG [Lactococcus...  36.6   2.1
ref|YP_001033759.1|  ATP-dependent DNA helicase RecG [Lactococc...  36.6   2.1
ref|NP_001161446.1|  ATP synthase, H+ transporting, mitochondr...  36.6   2.2
gb|AAX30619.2|  SJCHGC05868 protein [Schistosoma japonicum]        36.6   2.2
gb|AAY97888.1|  unknown [Schistosoma japonicum] >emb|CAX76370....  36.6   2.2
gb|EEY55604.1|  conserved hypothetical protein [Phytophthora i...  36.2   2.6
ref|XP_002298553.1|  predicted protein [Populus trichocarpa] >...  36.2   2.6
emb|CAX71056.1|  ATP synthase, subunit d [Schistosoma japonicum]   36.2   2.7
ref|XP_664235.1|  hypothetical protein AN6631.2 [Aspergillus n...  36.2   2.9
ref|XP_394036.1|  PREDICTED: similar to ATP synthase D chain, ...  35.8   3.3
emb|CAX76369.1|  ATP synthase, subunit d [Schistosoma japonicum]   35.8   3.8
 …
```

direct reciprocal BLAST of *M. brevicollis* protein with Mt_ATP-synt_D, ATP synthase D chain domain:
Query: JGI_XYM17876.rev EST translation *Monosiga brevicollis* MX1 length = 157
Subj: TTHERM_01188360_rev Length=234

Score = 39.3 bits (90), Expect = 4e-08, Method: Compositional matrix adjust.
 Identities = 22/47 (46%), Positives = 31/47 (65%), Gaps = 1/47 (2%)

```
Query  46   VAQPEPIDWAHYKSVINATGYVDEIKKAYDS-IKVEYPADTVSDQIS  91
            V Q   ID+   +KSVI+   G VD++K+ Y+S IK +Y   D +S QIS
Sbjct  72   VEQIPTIDFNEWKSVISTQGLVDKVKENYESLIKEQYNTDAISKQIS  118
```

Note: Revision (split) of *M. brevicollis* 1676 aa hypothetical protein with ATP synthase D chain domain indicated by cDNA:

```
>jgi|JGI_XYM17876.rev| JGI_XYM17876.rev
CCACGCGTCCGCTCTCTCTCTTTTTTTTCCTTTGTCCAACTGTTCAAAATGGCATCCCGTGAGGCTGGCAAGCGCGTCTTTGACTTTGCGGCCATGGTGGCCCGTGTGCCCGC
CAGCGCCCGCGCTGACGTGACGGCCCTGCGCAACGCCTACTTGGCCGTTCAGAGCAGCCTGGCCAAGACTGTCGCCCAGCCCGAGCCCATTGACTGGGCGCACTACAAGAGCG
TCATCAACGCCACTGGCTACGTTGATGAGATCAAGAAGGCCTACGACAGCATCAAGGTTGAGTATCCGGCCGACACCGTGTCGGACCAGATTTCTCAGGAGCAGAAGGCTGCC
GAGACAGAGGCTCAGCAGGTCATTGCCAAGGCTGATGCCCGCATTGCCGAGCTCAAGGCCCAGCTTGAGTCGATCAAGAGCGAGAAGCCCGTGGCTGATGTCACTGTTGACGA
GTTCCTGGCGGACAAGCCCGAGTGGCAGCGCGAGATTAAGGCTGAAATTGCCCAGCACAAGTACCAATAAATCACATTGGTACTGGCCGCATGCTCCTCCTTGGTCCCAGTCC
AAGAGACTGCGTGCTTCCATCCTTTTTCGCCCTCTTTGTACCACTGGCTTGTCTTCCGTGGCGGTCATGTCGCGACC

>JGI_XYM17876.rev EST translation [Monosiga brevicollis MX1]
MASREAGKRVFDFAAMVARVPASARADVTALRNAYLAVQSSLAKTVAQPEPIDWAHYKSVINATGYVDEIKKAYDSIKVEYPADTVSDQISQEQKAAETEAQQVIAKADARIA
ELKAQLESIKSEKPVADVTVDEFLADKPEWQREIKAEIAQHKYQ
```

(MitoProt II PROBABILITY of export to mitochondria: **0.5995)**

Queries of PFam database v 24.0. The top match for each ciliate putative **d** subunit is ATP synthase D chain, mitochondrial (ATP5H) Family, but only the match of the *Ichthyophthirius multifiliis* subunit is considered statistically significant.

header line key:
Family| Description| Entry type| Clan| Envelope{start\end}| Alignment{start\end}| HMM {from\to}| Bit score| E-value|

ICHmu_d E=0.00013
Mt_ATP-synt_D ATP synthase D chain, mitochondrial (ATP5H) Family n/a 33 131 65 113 42 90 21.3 0.00013

```
#HMM       aklpeklPkiDwayykknvpekaglvdsfekkyeal.kipyeedkylkeV
#MATCH     ++  ek+P+i w+ +kk+++ + glv++ +++ye l +  y+ d+  k+V
#PP        6889****************.9************9735566667777777
#SEQ       RQHTEKIPTINWEEWKKQIS-TKGLVEKVKENYETLiREQYQVDQIAKQV
```

TETth_d E= 0.00015
Mt_ATP-synt_D ATP synthase D chain, mitochondrial (ATP5H) Family n/a 42 138 70 120 43 93 21.1 0.00015

```
#HMM       klpeklPkiDwayykknvpekaglvdsfekkyeal.kipyeedkylkeVDae
#MATCH     +  e++P+iD++ +k  ++ + glvd+ +++ye+l k  y+ d  +k++
#PP        67799************9.************97588899898888888655
#SEQ       QHVEQIPTIDFNEWKSVIS-TQGLVDKVKENYESLiKEQYNTDAISKQISSA
```

PARte_d2 E=0.074
Mt_ATP-synt_D ATP synthase D chain, mitochondrial (ATP5H) Family n/a 47 125 65 105 50 90 12.3 0.074

```
#HMM       kiDwayykknvpekaglvdsfekkyeal.kipyeedkylkeV
#MATCH     +iDw+y+k+++  + glvd+ +k +e l    ++ ++  ++V
#PP        7***********.9**********99862344555555555
#SEQ       EIDWNYWKEQIT-TEGLVDKVQKGHETLlNKEFDVERICHQV
```

```
PARte_d1  E=0.088
Mt_ATP-synt_D ATP synthase D chain, mitochondrial (ATP5H) Family n/a 52 119 65 105 50 90 12.1 0.088

#HMM       kiDwayykknvpekaglvdsfekkyeal.kipyeedkylkeV
#MATCH     +iDw+y+k+++  + glvd+ +k ++ l +  ++ ++  ++V
#PP        7***********.9**********998624455555555555
#SEQ       EIDWNYWKEQIT-TEGLVDKVQKGHDTLlQREFDVERICHQV

-----------------------------------------------------------------
```

COMPASS generalized Psi-BLAST alignment profile - profile queries support significance of the similarities to the ATP synthase Delta/Epsilon domain:
Queries using alignment of 4 putative d subunit sequences from 3 ciliate species (see above).

COMPASS 3.0
Reference:
Ruslan I. Sadreyev and Nick V. Grishin (2003) COMPASS: a tool for comparison
of multiple protein alignments with assessment of statistical significance.
J Mol. Biol. 326(1):317-36
Job ID: ciliate_Fo_d

Query = ciliate_Fo_d.aln Input processed by alignment reader (please check!)
length=234; filtered_length=230; Neff=1.691
(threshold of effective gap content in columns: 0.5)

Database: PFAM 23.0
Effective database length used: 2236890

COMPASS alignment format:
CAPITAL letters: residues at positions aligned by COMPASS (input alignment positions with gap content < 0.5)
lower-case letters: residues at positions not used by COMPASS (input alignment positions with gap content >= 0.5)
'-' : gaps retained from original alignments at positions aligned by COMPASS (positions with gap content < 0.5)
'.' : gaps retained from original alignments at positions not used by COMPASS (positions with gap content >= 0.5)
'=' : gaps introduced by COMPASS in profile-profile alignment

'~' : gaps introduced by COMPASS against positions that are not used in the construction of profile-profile alignment
(positions with gap content >= 0.5)

Clickable sequence names: links to full alignments.
For more details, see documentation.

Hits with E-value BETTER than threshold:

Profiles producing significant alignments:	Score	E-value
Mt_ATP-synt_D ATP synthase D chain, mitochondrial (ATP5H)	81	2.20e-04
FH2 Formin Homology 2 Domain	93	5.15e-04

Hits with E-value WORSE than threshold (may be not biologically meaningful):

LACT Lecithin:cholesterol acyltransferase	69	2.82e-01
XH XH domain	60	3.02e-01
Cullin Cullin family	70	5.34e-01
PAD_porph Porphyromonas-type peptidyl-arginine deiminase	64	8.39e-01
APG6 Autophagy protein Apg6	63	8.53e-01
NHase_beta Nitrile hydratase beta subunit	59	9.86e-01

Alignments:

Subject = Mt_ATP-synt_D ATP synthase D chain, mitochondrial (ATP5H)

length=192 filtered_length=161 Neff=7.509
Smith-Waterman score = 81 Evalue = 2.20e-04

```
ICHmu_dr       69   EKIPTINWEEWKKQISTKGLVEKVKENYETLIREQYQVDQIAKQVLSQTSKPLDDIENEL  128
CONSENSUS_1    73   GAAPEIDWNEWKEQISTEGLVDKVKEGYETLLREEFDVDAIAKQVLSASSKPLDDLENEL  132
                    ++ P+IDW+++++ +  +GLVD++++ YE+ L+ ++ +D+   +++ +    + ++++E+ +
CONSENSUS_2    50   EKPPKIDWAFYRKNVPNPGLVDEFEKKYEA=LKIPYPEDRYTALIDAEEKEAVKEIEEFK  108
Q4PM92/2-161   46   ENPPPIDFAMYRSRLSNPALVDQFEKSYKS=FTVPFPKEHLTPQIDAEERQAKDEVEGFI  104
```

```
ICHmu_dr      129   SFHAAIWLNAYSDYTMFLFELEEYNNPNEYLMHENYDFFKGLEAELEELTETHNYIP  185
CONSENSUS_1   133   SFHAAVWLNAYADYTLFLLELEEYGDPNDYLLHEDYDFFPGLEAELEELTETHNWIP  189
                    +  +A   ++  +    L +L++     + +L + ++ FP L  +++   ++++++P
CONSENSUS_2   109   KESEARIKEYEKE====LEKLKALIPFDEMTLEDFAEAFPELALDPL===NKPTFWP  158
Q4PM92/2-161  105   RESKERIEGFKQE====LLKFQAMIPAAHMTLEDYADYFPEHALN-V===DKPTYWP  153
```

Parameters:
E-value cutoff: 1.000e+00
Maximal number of hits to display: 100
Lambda_ungapped: 3.4578e-01
Expected value of positional scores: -2.8043e-01
Gap opening: 10
Gap extension: 1

COMPASS 3.0
Reference:
Ruslan I. Sadreyev and Nick V. Grishin (2003) COMPASS: a tool for comparison of multiple protein alignments with assessment of statistical significance. J Mol. Biol. 326(1):317-36
Job ID: ciliate_Fo_d

Query = ciliate_Fo_d.aln Input processed by alignment reader (please check!)
length=234; filtered_length=230; Neff=1.691
(threshold of effective gap content in columns: 0.5)

Database: PDB70_iter5
Effective database length used: 3663686

Hits with E-value BETTER than threshold:

Profiles producing significant alignments:	Score	E-value
2CLYB Subcomplex Of The Stator Of Bovine Mitochondrial Atp	89	4.70e-06

Hits with E-value WORSE than threshold (may be not biologically meaningful):

3BHWA Crystal Structure Of An Uncharacterized Protein From	60	8.11e-01

Alignments:

Subject = 2CLYB   Subcomplex Of The Stator Of Bovine Mitochondrial Atp

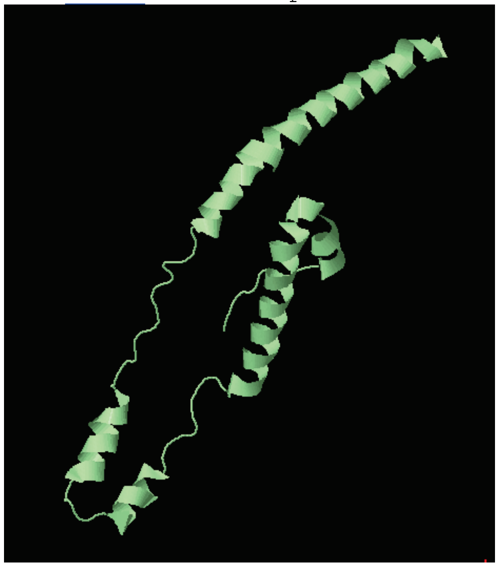

```
length=120  filtered_length=119  Neff=9.815
Smith-Waterman score = 89   Evalue = 4.70e-06

ICHmu_dr     69    EKIPTINWEEWKKQISTKGLVEKVKENYETLIREQYQVDQIAKQVLSQTSKPLDDIEN  126
CONSENSUS_1  73    GAAPEIDWNEWKEQISTEGLVDKVKEGYETLLREEFDVDAIAKQVLSASSKPLDDLEN  130
                   ++ P+IDW+++++++ ++GLVDK+++ YE+ L+  + VD+  +++ +  ++ L+++++
CONSENSUS_2  43    EEPPKIDWAHYRKVLGNPGLVDKFEKAYEA=LKVPYPVDKYTALIDAEEKEALKEAKE  99
2CLYB        43    EKPPAIDWAYYKANVAKAGLVDDFEKKFNA=LKVPIPEDKYTAQVDAEEKEDVKSCAE  99
```

Parameters:
E-value cutoff: 1.000e+00
Maximal number of hits to display: 100
Lambda_ungapped: 3.4578e-01

Expected value of positional scores: -2.8043e-01
Gap opening: 10
Gap extension: 1

COMPASS 3.0
Reference:
Ruslan I. Sadreyev and Nick V. Grishin (2003) COMPASS: a tool for comparison of multiple protein alignments with assessment of statistical significance.
J Mol. Biol. 326(1):317-36
Job ID: ciliate_Fo_d

Query = ciliate_Fo_d.aln Input processed by alignment reader (please check!)
length=234; filtered_length=230; Neff=1.691
(threshold of effective gap content in columns: 0.5)

Database: KOG
Effective database length used: 2777205

Hits with E-value BETTER than threshold:

Profiles producing significant alignments:		Score	E-value
KOG3366	Mitochondrial F1F0-ATP synthase, subunit d/ATP7	78	1.75e-03

Alignments:

Subject = KOG3366 Mitochondrial F1F0-ATP synthase, subunit d/ATP7

length=222 filtered_length=175 Neff=4.669
Smith-Waterman score = 78   Evalue = 1.75e-03

```
ICHmu_dr     69   EKIPTINWEEWKKQISTKGLVEKVKENYETLIREQYQVDQIAKQVLSQTSKPLDDIENEL  128
CONSENSUS 1  73   GAAPEIDWNEWKEQISTEGLVDKVKEGYETLLREEFDVDAIAKQVLSASSKPLDDLENEL  132
                  +++P+IDW+++++ +   GLVDK+++ Y++L   ++ V+ + +++L++     + +  E+
CONSENSUS 2  71   ENPPTIDWAYYRKNLANAGLVDKFEKKYDSL===KIPVPIDLDKYLKEVDAEEKAALKEI  127
```

```
SPBC29A10.13 50   EQATTVDFATYRSVLANKEIVNRIESSMKSF===-KPVKIDLNSQLKAINAFEAKASEGA  105

ICHmu_dr     129  SFHAAIWLNAYSDYTMFLFELEEYNNPNEYLMHENYDFFKGLEAELEELTETHNY  183
CONSENSUS 1   33  SFHAAVWLNAYADYTLFLLELEEYGDPNDYLLHEDYDFFPGLEAELEELTETHNW  187
                  + + +  +    +++  L ++++    +++ + + ++ FP L+ ++++     +W
CONSENSUS 2  128  KEYESLVKKRLQELEKELEKVKSLRPFDELTIEDLCEAFPELKLDFINKPTFGKW  182
SPBC29A10.13 106  KKNVELVKAELQNLSATLKNIEQARPTEEITIEDMKQAVPEIEKIVETMVTKGKW  160
```

Parameters:
E-value cutoff: 1.000e+00
Maximal number of hits to display: 100
Lambda_ungapped: 3.4578e-01
Expected value of positional scores: -2.8043e-01
Gap opening: 10
Gap extension: 1

COMPASS 3.0
Reference:
Ruslan I. Sadreyev and Nick V. Grishin (2003) COMPASS: a tool for comparison of multiple protein alignments with assessment of statistical significance.
J Mol. Biol. 326(1):317-36
Job ID: ciliate_Fo_d

Query = ciliate_Fo_d.aln
length=234; filtered_length=230; Neff=1.691
(threshold of effective gap content in columns: 0.5)

Database: COG
Effective database length used: 1694298

**** No hits found ****
(as expected: subunit **d** is only known in Eukaryotes)

Parameters:
E-value cutoff: 1.000e+00
Maximal number of hits to display: 100
Lambda_ungapped: 3.4578e-01
Expected value of positional scores: -2.8043e-01

Gap opening: 10
Gap extension: 1

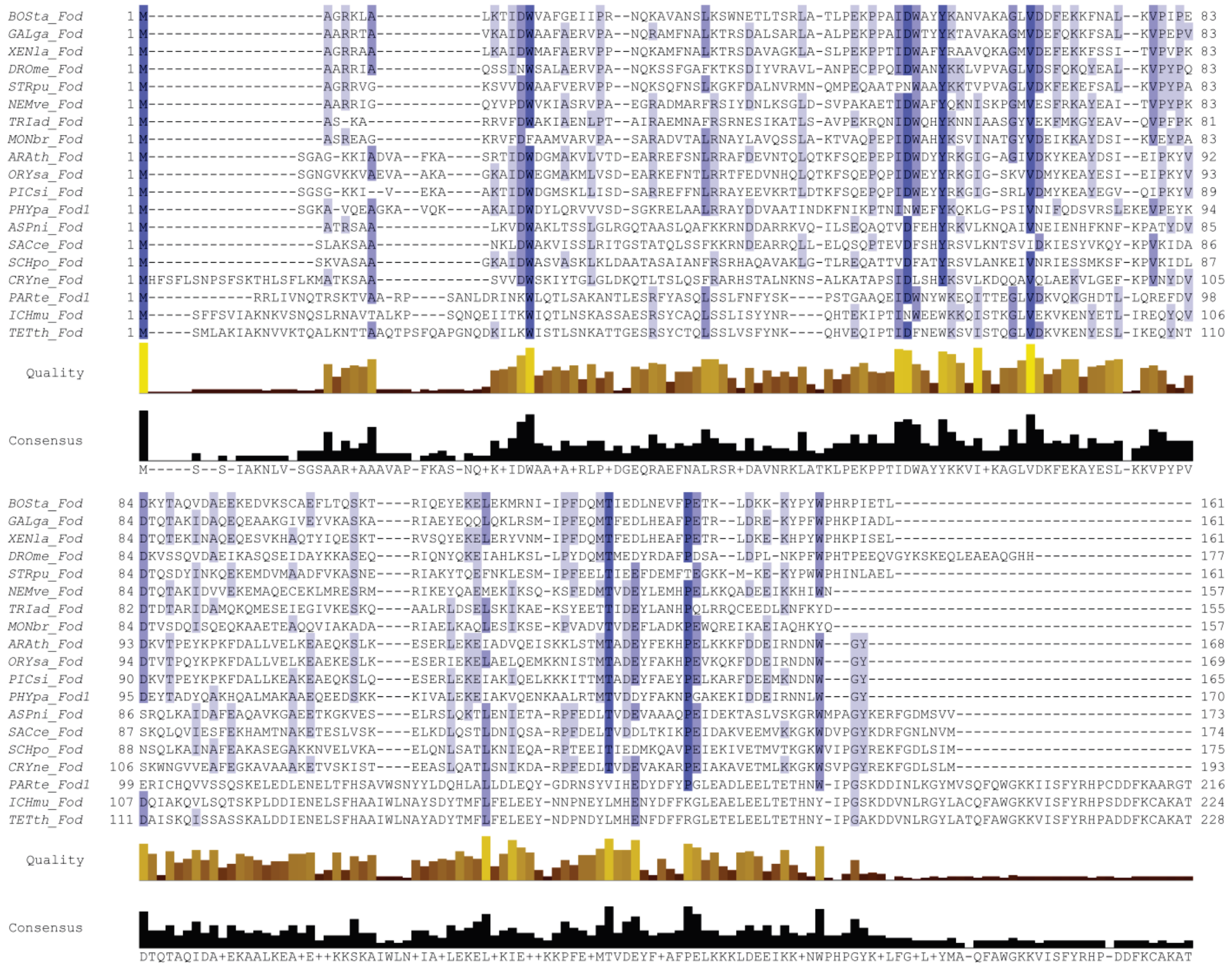

Alignment to broad phylogenetic range of known and putative d subunits.

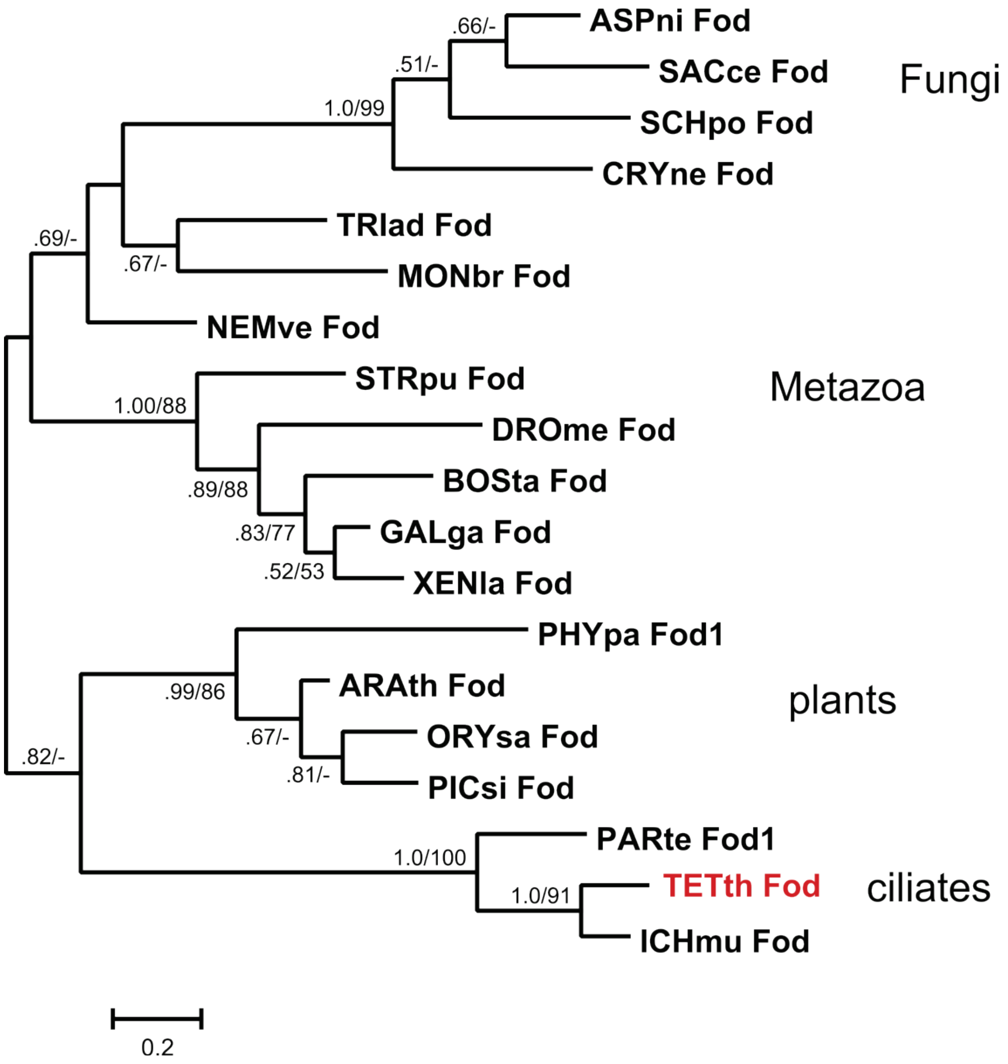

Phylogram resulting from phylogenetic analysis by Bayesian inference. Similar results were obtained by maximum likelihood analysis. Numbers near branch nodes indicate Bayesian posterior probabilities/maximum likelihood bootstrap support; - indicates maximum likelihood support of less than 50%.
